# Climate shapes mammal community trophic structures and humans simplify them

**DOI:** 10.1038/s41467-019-12995-9

**Published:** 2019-11-15

**Authors:** Manuel Mendoza, Miguel B. Araújo

**Affiliations:** 1 Departamento de Biogeografía y Cambio Global, Museo Nacional de Ciencias Naturales, CSIC, c/ Jose Gutierrez Abascal, 2, 28006 Madrid, Spain; 2 Rui Nabeiro Biodiversity Chair, MED Institute, Universidade de Évora, Largo dos Colegiais, 7000 Évora, Portugal; 3 Center for Macroecology, Evolution and Climate, GLOBE Institute, University of Copenhagen, DK-2100 Copenhagen, Denmark

**Keywords:** Biogeography, Food webs, Macroecology

## Abstract

Nature’s complexity is intriguing, but the circumstances determining whether or how order emerges from such complexity remains a matter of extensive research. Using the geographical distributions and food preferences of all terrestrial mammal species with masses >3 kg, we show that large mammals group into feeding guilds (species exploiting similar resources) and that these guilds form trophic structures that vary across biomes globally. We identify five trophic structures closely matching climate variability and named them boreal, temperate, semiarid, seasonal tropical and humid tropical owing to their relative overlap with the distribution of biomes. We also find that human activities simplify trophic structures, generally transitioning them to species-poorer states. Detected transitions include boreal and temperate structures becoming depauperate or seasonal- and humid-tropical becoming semiarid. Whether the observed generalities among trophic structures of large mammals are indicative of patterns across whole food webs is matter for further investigation. The results help refine projections of the effects of environmental change on the trophic structure of large mammals.

## Abstract (web summary)

Broad scale patterns in the distribution of animal community functional properties could be determined by climate and disrupted by human activities. Here the authors show global patterns in large-mammal trophic structure related to climate variation, which human activities simplify in predictable ways.

## Introduction

Before embarking on his expedition to South America, Alexander von Humboldt wrote^1^: “I shall endeavour to find out how nature's forces act upon one another, and in what manner the geographic environment exerts its influence on animals and plants.” His observations that changes in vegetation structure match changes in temperature along elevation gradients became highly influential^2^. They were the first of a long series of observations establishing that the broad outlines of species distributions are generally limited by climate and change in synchrony with changes in climate^3^. Theory and underlying assessments of climate change effects on biodiversity are grounded on such observations^4^. Experiments have challenged the view that climate alone determines species distributions. For example, a microcosm experiment showed that responses of Drosophila species to climate were modified by the presence of parasitoid wasps^5^. Field experiments also revealed that interactions among plants could switch from being predominantly negative to becoming predominantly positive along elevation^6^ and water-stress gradients^7^, demonstrating that interactions among species can both modify the environment and the species responses to it. The degree to which biotic interactions modify the responses of species to climate and, therefore, alter range limits at biogeographic scales is still a matter of ongoing debate^8–11^, but the extent to which climate influences the biotic interactions that can coexist in any particular place is much less explored^12,13^ and is the focus of our research.

The prevailing view is that distributional dynamics at broad scales are best characterized by individualistic responses of species to climate and that biotic interactions play a minor role in this process^14,15^. Reliance on this idea has been supported by the analysis of multiple species response curves along environmental gradients, typically showing that species’ critical environmental limits and optima vary independently of one another^3^. Analyses of the fossil record further support the view that species respond individualistically to their environment by revealing that species associations, at any particular time, are generally not predicting species associations at different times following environmental change^16^. A contrasting view emerges when functional aspects of communities are analysed^17,18^. Trophic structures, in particular, show striking stability over time even when species composition is severely changed^19,20^. The idea of energetic controls on communities dates back to the concept of vegetation biomes proposed by Humboldt^2^, further expanded by authors including Slobodkin^21^, Odum^22^, Lindeman^23^, and Whittaker^24^, proposing that functional properties of local communities are directly related to the physical environment, chiefly climate. Whether similar generalisations can be made for animal communities at biogeographic scales remains a matter of inquiry^8,25,26^. Contemporary studies of local trophic structures, namely food webs in aquatic systems, have found that they share structural features across climatically similar areas, but relationships tend to be weak and multiple variables compete as best predictors^27–29^. It is conceivable that, globally, general patterns in the distribution of trophic structures should emerge while local variation would be overridden by environmental turnover, but empirical evidence for such prediction is lacking. If broad-scale functional properties of communities, such as trophic structures, could be inferred from characteristics of the physical environment, then projections of environmental change effects on these functional properties could be made without detailed knowledge of the myriad direct and indirect biotic interactions that occur in nature. Such a simplification—already common in vegetation science^30^—would represent a major step towards improving the understanding of how communities are distributed on earth and how they will change under future global environmental change scenarios.

We apply a range of clustering and machine learning analyses to data on the distributions and feeding preferences of large mammals around the world to examine patterns in the distribution of community trophic structures (characterised as identifiable clusters of trophic guilds), and their relationships with climate and human development. Starting with the observation that climate determines the amount of energy available for consumption by animals via its effects on vegetation, we predict that different, well-defined, community trophic structures exist around the world and that differences among them are related to variation in climate. We identify five community-trophic structures among large mammal species broadly matching the distribution of biomes (boreal, temperate, semiarid, seasonal tropical, and humid tropical). We also identify a sixth species-poor type (depauperate) matching a variety of climatic and non-climatic limiting factors. Having found support for both predictions, we foresee that human disturbances should reduce the amount of energy available to animal communities, thereby leading to simplification of the mammal trophic structures expected given the climate. The prediction is also supported by the data with areas featuring high human impacts closely matching the distribution of simplified community trophic structures.

## Results

### Identification of community trophic structures

**Fig. 1:**
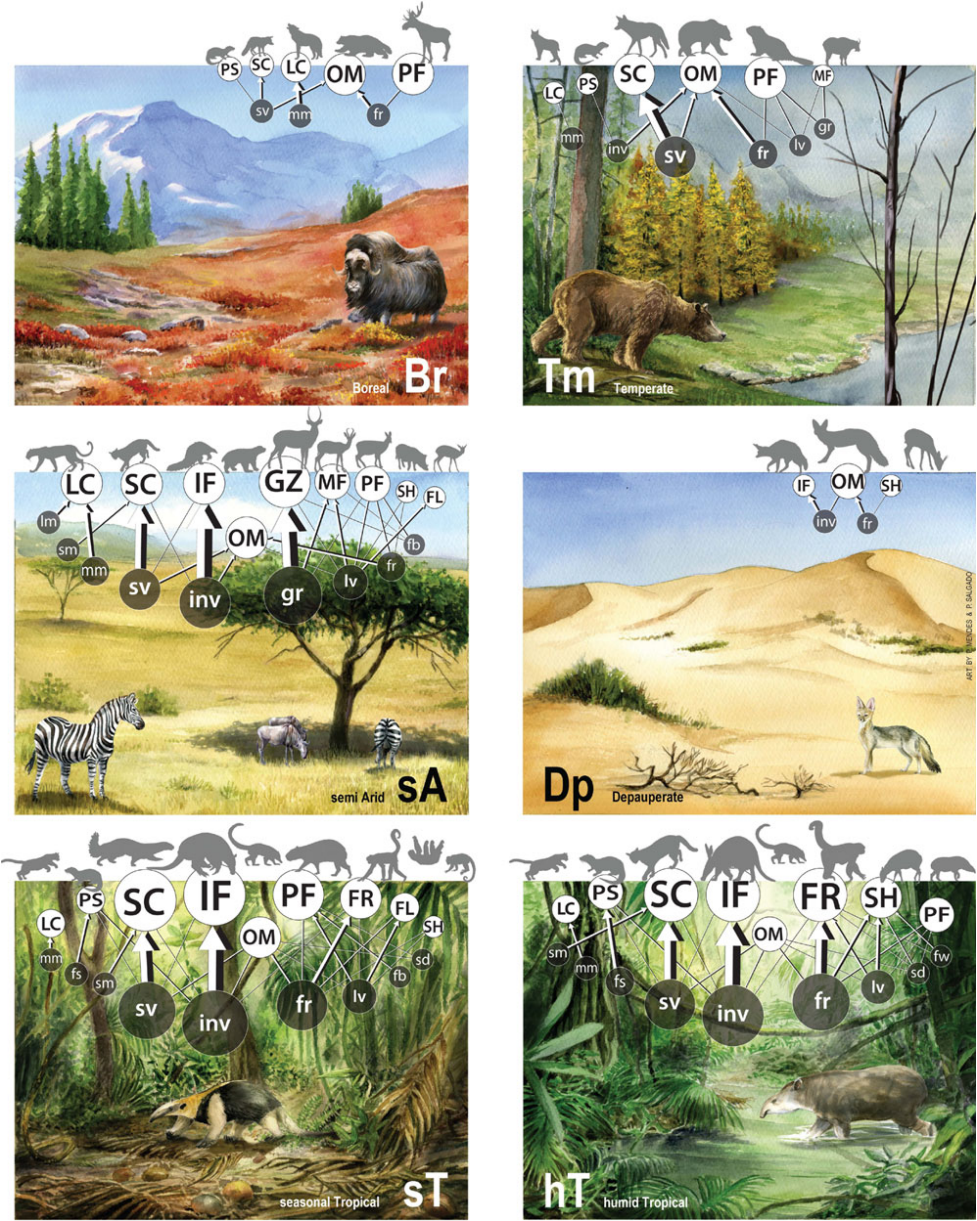
Six types of mammal trophic structures around the world: boreal, temperate, semi-arid, seasonal tropical, humid tropical, and depauperate. For convenience, trophic structures are represented as bipartite networks depicting energy flows between trophic guilds and their main resources. Resources: grass gr, leaves lv, fruits fr, flowers fw, forbes fb, seeds sd, invertebrates inv, fish fs, small vertebrates sv, mammals (1–10 kg) sm, 10–100 kg mm, (>100 kg) lm. Trophic guilds: selective herbivores SH, plant material feeders PF, small carnivores SC, frugivores FR, folivores FL, mixed feeders (being both grazers and browsers), MF, omnivores OM, grazers Gz, piscivores PS, invertebrate feeders IF, large carnivores LC. The size of the nodes for the guilds (white) is proportional to the number of species from the guild in the food web. Arrows represent the fluxes of energy from resources to guilds. The magnitude of the fluxes is represented by the thickness of the arrows and increases with the sum of the estimated percentage of that resource in the diet of all the species in that guild. The size of the nodes for resources (black) is proportional to their total contribution to the web. This contribution is equivalent to the sum of the estimated percentage of that resource in the diet of all species

**Fig. 2:**
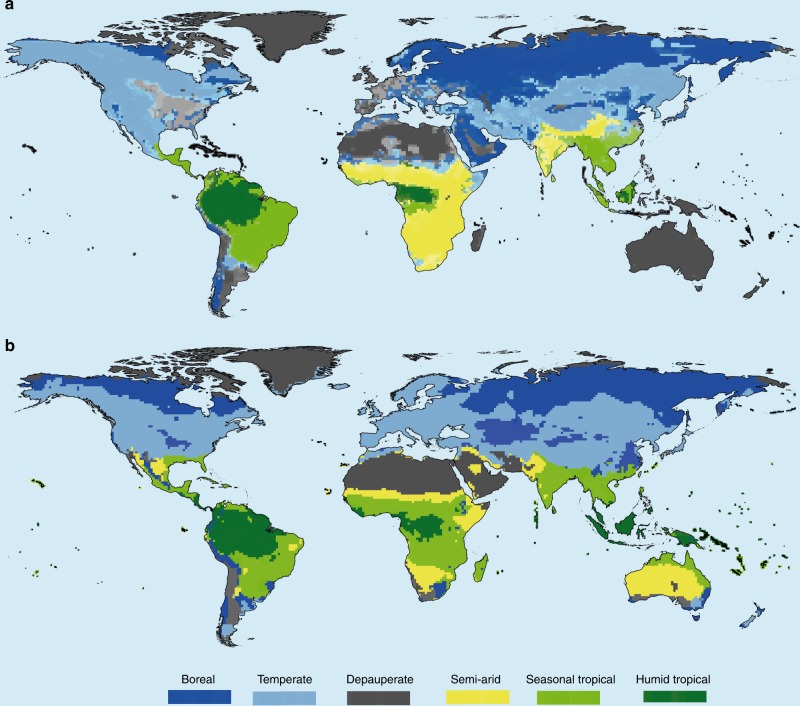
Geography of large-mammal trophic structures around the world. **a** Distribution of observed trophic structures. **b** Distribution of predicted trophic structures. Predictions were obtained by regressing observed trophic structures in low impacted cells against climate variables and then interpolating them to the world given climate data (see “Methods”). Colour intensity within each colour class in **a** is related to the probability of cells belonging to the trophic structure it has been classified into (see “Methods”)

We examined distributions and trophic preferences of all known species of terrestrial mammals with >3 kg (689 species). Based on extensive literature review (Supplementary Data File 1), we identified 19 trophic resources consumed by large mammals. We clustered mammals based on their food preferences, a process leading to identification of 11 consumer strategies or guilds (Supplementary Data File 2). The trophic structure of the mammal assemblages within each terrestrial 1º × 1º grid cell was determined based on the number of species from each guild that occurred therein. We explored the existence of distinct configurations in trophic structures by fuzzy clustering guild assemblages across the terrestrial grid cells of the world. To minimize biases arising from human disturbances, we restricted analysis to cells with lower human impacts. A machine learning approach was used to interpolate the six trophic structures across the remaining cells not used to characterize them (see “Methods”). With this process we uncovered six well-defined trophic structures of large-mammal communities globally (Fig. 1; Supplementary Fig. 2d). They differ in species numbers and proportion of guilds (Supplementary Figs. 3b, 4), having well-defined distributions (Fig. 2a). Five of them have seemingly clear climatic distributions, moderately matching vegetation biomes (Kappa statistic = 0.24)^24^ (Fig. 2a; Supplementary Fig. 6). The most widely distributed trophic structure is found mainly at high latitudes (occupying 2584 lower impact cells; Fig. 2a), hence termed boreal; it has small numbers of species, relatively greater numbers of omnivores, followed by large carnivores, and plant feeders (Fig. 1, Supplementary Fig. 4). The next most widely distributed trophic structure was termed temperate (2151 cells; Fig. 2a), having more species than the boreal type and relatively greater numbers of plant feeder species, followed by mixed feeders, omnivores, large carnivores, and small carnivores (Fig. 1, Supplementary Fig. 4). The humid tropical (491 cells) and seasonal tropical (437 cells) trophic structures are found around the equatorial belt (Fig. 2a) being more species rich than boreal and temperate trophic structures (Fig. 1, Supplementary Figs. 3b, 4). Humid tropical structures have greater numbers of frugivores, followed by small carnivores and invertebrate feeders, whereas small carnivores, plant feeders, and invertebrate feeders, followed by omnivores and frugivores, characterize seasonal tropical trophic structures (Fig. 1, Supplementary Fig. 4). Semi-arid trophic structures (267 cells; Fig. 2a) have the greatest number of large mammal species and are dominated by omnivores, grazers, plant feeders followed by small numbers species from several other guilds (Fig. 1, Supplementary Fig. 4).

Unlike the former five community trophic structures, the sixth type is not always climatically determined. We labelled it depauperate (2132 cells; Fig. 2a; Supplementary Fig. 3a) because it features small numbers of species (Fig. 1, Supplementary Fig. 4). Depauperate trophic structures are found in a variety of regions, including areas with extreme climatic conditions, such as hot and dry deserts, the Arctic, but also in Western Europe and Eastern North America. All oceanic islands (including Australia and Madagascar) have depauperate trophic structures.

### Relationship between community trophic structures and climate

After mapping the six trophic structures, we modelled their relationship with climate. Evolutionary learning of globally optimal classification trees was used to predict the geographical distribution of trophic structures in relation to climate. The predicted structures match vegetation biomes more closely than the observed ones (Kappa increasing from 0.24 to 0.43; Fig. 2b, Supplementary Fig. 6).

**Fig. 3:**
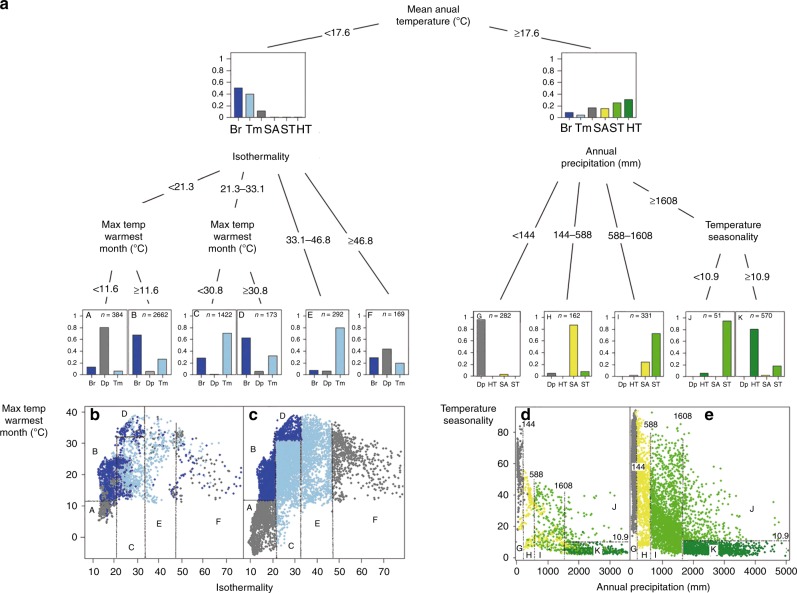
Relationship between climatic predictors and mammal trophic structures. **a** Tree model relating climate predictors and trophic structures and splitting them into a cold (left) and warm (right) branch. **b** Plot of observed trophic structures against the climate space defined by the two variables best explaining the cold branch (Mean annual temperature < 17.6 °C). **c** The same as **b** but using predicted rather than observed trophic structures. **d** The same as b, but in the space defined by the two variables best explaining the warm branch (mean annual temperature ≥ 17.6 °C). **e** The same as **d**, but using predicted rather than observed trophic structures. Dots in panels **b**, **c**, **d**, and **e** are cells on the world map. Capital letters in panels b, c, d, and e correspond to the final groups in panel a. *Br* Boreal, *Tm* Temperate, *SA* Semi-arid, *ST* Seasonal tropical, *HT* Humid tropical, *Dp* Depauperate. Isothermality = Mean diurnal range temperature/temperature annual range, Temperature Seasonality = The amount of temperature variation over a given period based on the standard deviation of monthly temperature averages

This process also enabled the identification of thresholds beyond which certain combinations of climate variables are linked with changes in trophic structures (see “Methods”). The first threshold (annual temperature below or above 17.6 °C) splits trophic structures from cold and warm environments (Fig. 3a). The cold group encompasses depauperate, boreal, and temperate structures (Fig. 3b, c). The warm group encompasses mainly depauperate, semiarid, seasonal-, and humid-tropical structures (Fig. 3d, e). Taking depauperate structures aside, the split between warm and cold trophic structures is largely associated with variations in species richness (see “Methods”). Further splits are largely independent of species richness. Trophic structures of cold environments are discriminated by isothermality (the ratio between mean diurnal range and temperature annual range) and maximum temperature of the warmest month. Cells with >21.3 isothermality and <11.6 °C maximum temperature of the warmest month are generally classified as depauperate (group A, Fig. 3) and found near the Arctic. Above these lower limits, boreal or temperate structures predominate (groups B, C, D, and E, Fig. 3). However, isothermality >46.8 (group F, Fig. 3) does not fully explain differences in the distribution of trophic structures with precipitation of the wettest month and temperature of the wettest quarter explaining additional variation (Supplementary Fig. 5). Differences among warm structures are explained by annual precipitation and temperature seasonality (the amount of temperature variation over a given period based on the variation of monthly temperature averages). With precipitation <144 mm, structures lean towards depauperate state typically found in deserts (group G, Fig. 3). Between 144 and 588 mm, semiarid structures predominate (group H, Fig. 3) and between 588 and 1608 mm seasonal tropical structures emerge even when exposed to lower temperature seasonality (group I, Fig. 3). With annual precipitation >1608 mm humid tropical trophic structures emerge but only when the temperature seasonality is lower than 10.9 (groups J and K, Fig. 3).

### Impact of human activities on community trophic structures

**Fig. 4:**
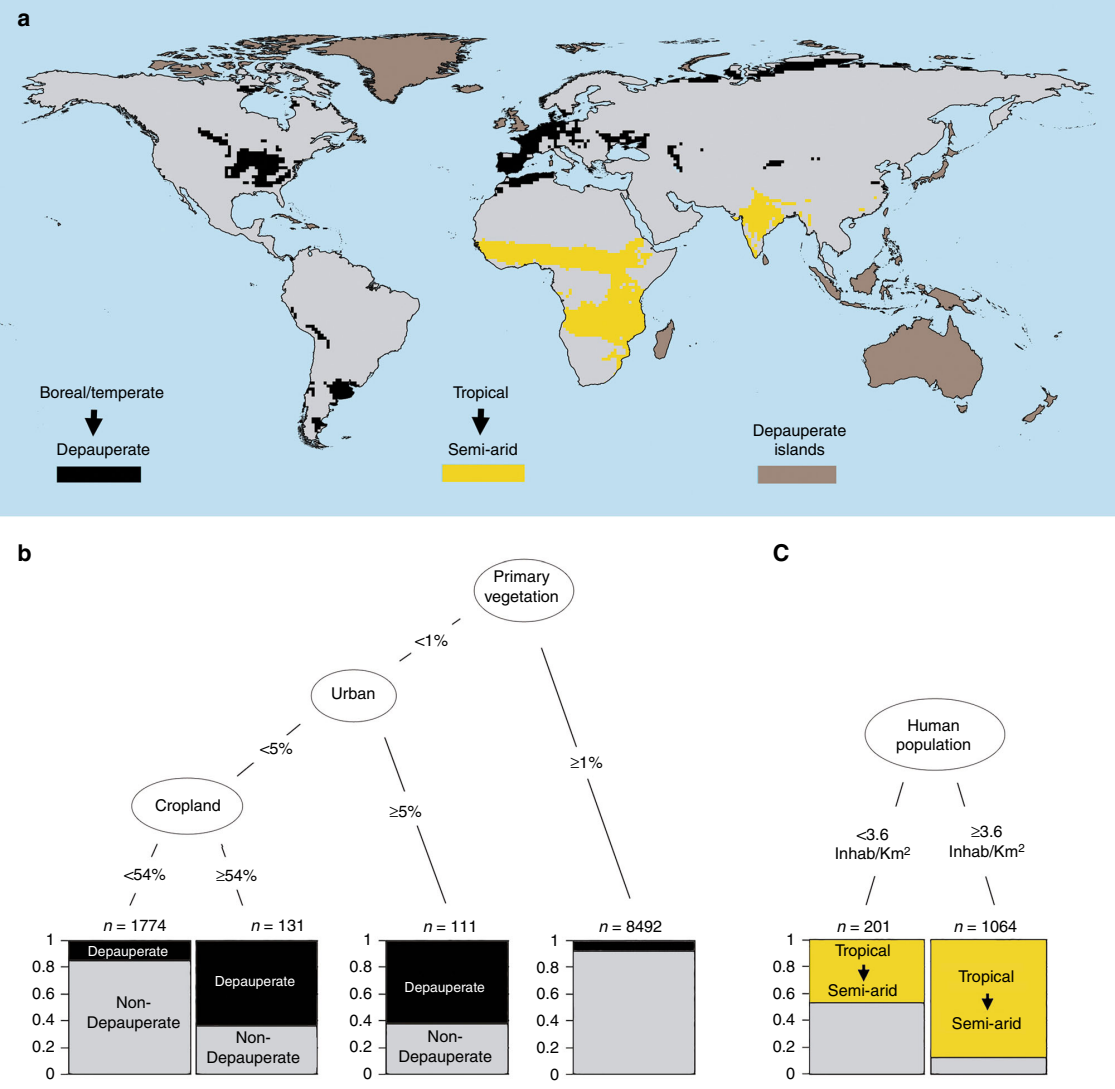
Relationship between transitions in trophic structures and human impacts. **a** World distribution of differences between observed and predicted trophic structures (i.e., transitions). **b** Tree model linking human impacts to trophic structure transitions from boreal or temperate to depauperate. **c** Tree model linking human pressures to trophic structure transitions from tropical to semi-arid. Terminal nodes indicate the number of cells in the category (*n*) and the ratio of cells in each category (indicated in the *y*-axis, see text)

Evolutionary learning of globally optimal classification trees was also used to determine the effect of human disturbances on trophic structures. We first predicted the expected climatic distribution of trophic structures in the absence of human impacts and then compared observed trophic structures (Fig. 2a) with predicted ones (Fig. 2b). Observed structures differing from predicted ones invariably represent depauperate structures or simplified versions (Fig. 4a). Spatial congruence between observed and predicted trophic structures occurs when human impacts are lower. Spatial mismatches occur when impacts are higher (Supplementary Table 1). Predicted depauperate trophic structures are restricted to limited energy regions, such as the arctic and hot and dry deserts. By contrast, trophic structures from Western Europe and Eastern North America are classified depauperate but predicted temperate (Fig. 2b). Human impacts are also higher in several areas across South Asia and Sub-Saharan with observed trophic structures being classified as semi-arid (Fig. 2a) but being predicted as seasonal tropical (Fig. 2b).

To explore the effects of human impacts on trophic structures, we compared areas with mismatches between observed and predicted trophic structures with a range of human development variables including human population density and land cover types (Fig. 4b,c). Results indicate that trophic structures of boreal and temperate environments collapse into depauperate state when land-use pressures are high (Fig. 4b). When primary vegetation covers ≥1% of the cells (~123 km^2^), boreal and temperate trophic structures are found in 80% of the cases. With <1% primary vegetation, predicted trophic structures match observed trophic structures when urban landscapes cover <5% of cells and croplands <54% (Fig. 4b). Shifting tropical trophic structures into semiarid structures are related to human population density, with most shifts occurring with >3.6 inhabitants per km^2^ (Fig. 4c).

All oceanic islands, including Australia, have trophic structures simplified with regard to their climate potential. It could be that mammal colonization of islands was uneven among guilds, thus causing some guilds to be absent^31^. Alternatively, it could be that human-driven extinctions were selectively sparing the more generalist species^32,33^.

## Discussion

Our results suggest that trophic structures converge when communities are exposed to similar environments, a phenomenon analogous to convergent evolution of similar traits and functions across lineages exposed to similar conditions. Our analysis focuses on a subset of regional trophic relationships in large mammals; whether results are general across feeding groups will require analyses with more groups of organisms. But climate, through the combined effects of radiation, temperature, and water, determines the amount of energy available through photosynthesis. On Earth, different combinations of climate variables yield different combinations of resources. The tropics, for example, are rich in fruits, thus offering great opportunities for frugivores to increase biomass and diversify. Savannahs, in contrast, are rich in herbs, bushes, and low trees, thus offering great opportunities for grazers and browsers. It follows that if available energy determines the quantities and types of feeding groups that are likely to co-occur within communities (Fig. 1), changes in the amount of energy should lead to predictable changes in the community trophic structure. Now suppose a species—the human species—was able to consume meaningful quantities of available energy so that the net primary productivity of ecosystems was significantly reduced. The logical prediction is that reductions of available energy by human consumption—perhaps interacting with other human impacts, such as fragmentation—should cause trophic structures to switch into simpler forms. This is what we found. Humans globally harvest ca. 25% of the total net primary production^34^, but the magnitude of the harvesting is unequally distributed. Consistent with our prediction, we found that areas more exposed to human impacts, measured through a variety of indicators (see Fig. 4), have community trophic structures simplified with regard to predicted structures with climate. Ideally, one would measure flows of energy percolating through ecosystems. That is, quantify the energy reaching ecosystems from the sun, made usable by the presence of water, and then subtract the energy appropriated by animals and humans^35^. We cannot yet measure food webs involving all organisms in communities, let alone quantify the full energetic balance of ecosystems. That we could identify climatic and human-impact thresholds, or tipping points, delimiting shifts between seemingly stable trophic structures involving several hundreds of organisms is promising. It opens new opportunities for describing and modelling global environmental change impacts on the functional characteristics of animal communities, thus moving beyond familiar approaches focusing on individual species distributions^36^.

## Methods

### Data

Five different sources of data were used: global distributional ranges of mammal species with >3 kg; species feeding preferences, bioclimatic variables, land-use variables, and human population density. All geographical data were plotted in a world terrestrial 1º × 1º grid system.

The global distribution maps of mammal species were derived from IUCN Global Assessment of native ranges^37^. The distributions of species were matched to the taxonomies provided by Wilson^38^ following Fritz and Purvis^39^. Occurrences in grid cells were used to produce a presence/absence matrix with names of the 689 macro-mammal species as columns and the 18418 1º x 1º grid cells as rows. No records for species occurring in Antarctica were used.

Nineteen trophic resources consumed by mammals were identified based on an extensive literature review (Supplementary Data File 1). The estimated percentage of each type of resource in the diet of the 689 large mammal species was established according to information available in the Animal Diversity Web^40^, the Ultimate Ungulate Page and with the help of the references included in Supplementary Data File 1. The resulting trophic-preferences matrix, with the 19 trophic resources as columns and the names of the species as rows, is also provided as Supplementary Data File 2.

Bioclimatic data for the terrestrial surface of the earth were obtained from WorldClim-Global Climate Data^41^. We used the full set of 19 bioclimatic variables available in WorldClim for exploration of bioclimatic correlates of trophic structures. These variables represent annual trends (e.g., mean annual temperature, annual precipitation), seasonality (e.g., annual range in temperature and precipitation), and extreme or limiting climatic factors (e.g., temperature of the coldest and warmest month, and precipitation of the wet and dry quarters) commonly used to model aspects of biodiversity in relation to climate.

Global land-use data were obtained from the Harmonization of Land-Use Scenarios^42^. The data included the percentage of surface of each cell covered by cropland, pasture, primary vegetation, secondary vegetation, and urban land.

Human Population data were obtained from the Gridded Population of the World (GPWv3) for the year 2000. GPWv3 consists of estimates of human population by 2.5 arc-min grid cells^43^. Human population within each 1º × 1º grid cell was obtained through a zonal operation returning the mean values from 1 km^2^ cells (the first dataset) that fall within each 1º x 1º square/zone of the study area.

### Low human impact cells

Human actions directly or indirectly impact on ecosystem dynamics, hence being expected to affect trophic structures^44,45^. To minimize potential biases arising from human disturbances, we restricted the analysis leading to the identification of trophic structures to 8062 cells with lower human impact (out of the total 18418 grid cells), defined as those with ≥25% of the cell covered with primary vegetation, <0.003% covered with urban surface (lower than the world mean), and with human population <26 hab/km^2^ (third quartile).

### Objective 1: identifying trophic guilds

Trophic guilds are typically defined by the diet species have^46^. When species depend on a single type of resource, the classification is straightforward: a species only eating fish is unequivocally a Piscivore. But when species depend on several resources, with varying degrees of dependence on them, the classification of species into guilds becomes fuzzier. Trophic guilds are commonly based on arbitrary thresholds regarding the percentage of each type of resource in the diet of the species^46^. Here, trophic guilds were identified with c-means clustering^47–49^ on the basis of the Euclidean distance between the 689 species in the 19-dimensional space defined by the estimated percentage of each type of resource in their diet (a vector of dimension 19). Clustering analyses were performed using R statistical software with the package e1071^50^. Eleven trophic guilds were defined and named consistently with diet categories commonly used in the literature (Supplementary Fig. 1).

### Objective 2: detecting similar trophic structures

The trophic structure of mammal communities in every grid cell was characterized based on the number of species from each trophic guild that occurred therein. As a starting point, we assigned each species to its correspondent guild and then counted the number of species of each guild within each cell. The result is a matrix with the 11 trophic guilds as columns, 18,418 1º × 1º grid cells as rows, and values in the table representing the numbers of species. The trophic structure in each cell is thus a point in an 11-dimensional ‘trophic space’ defined by the number of species from each trophic guild (a vector of dimension 11). Should there be alternative configurations in trophic structures^51–57^, one would expect cells not to be homogeneously distributed in the 11-dimensional trophic space and rather display well-defined clusters bringing together cells with a similar pattern in their trophic structure.

To identify these groups of cells with a common pattern in their trophic structure, we used fuzzy clustering. In ‘hard’ clustering, data are divided into distinct clusters whereby each element belongs to one cluster. In fuzzy or ‘soft’ clustering, elements show degrees of membership to each cluster (a value between 0 and 1), which is linked to their squared Euclidean distance to the geometric centre of the cluster. When a clustering analysis is done with a number of clusters equal to the number of groups that actually exist in the data, the expectation is for clusters to be more separated (different) and compact (well defined). Indeed, most measurements of cluster validity quantify the degree of separation and the compactness of the clusters (e.g., Davies-Bouldin or Dunn Indices). In fuzzy clustering, clusters with elements showing a high degree of membership are considered well-defined clusters, whereas clusters with many elements with unclear membership are considered diffuse or fuzzy. Hence, we calculated an index of average membership degree (AMD_index_) of the samples making up the clusters:

1 $${\mathrm{AMD}}_{{\mathrm{index}}} = \frac{1}{n}\mathop {\sum }\limits_{j = 1}^{j = n} {\mathrm{MD}}_j - \frac{1}{c},$$

where MD_*j*_ is the membership degree of sample *j* to the nearest cluster, *n* is the number of samples, and *c* is the number of clusters.

Well-defined groups in our 11-dimensional trophic space (determined by the number of species from each trophic guild) are interpreted as indicating the existence of distinct configurations in trophic structures. Both the existence of well-defined clusters and their numbers can be detected by tracking changes in the AMD_i_ through a series of clustering analysis runs with increasing numbers of user-defined clusters (hereafter referred to as ‘AMD_i_ approach’). Before applying the AMD_i_ approach to our 11-dimensional ‘trophic space’, we tested it with computer-generated samples. The degree of membership of randomly distributed samples is equal to the inverse of the number of clusters (1/c). AMD_i_ is, therefore, always zero when the artificial samples are randomly distributed (Supplementary Fig. 2a). Supplementary Fig. 2b shows the curve resulting from the application of the AMD_i_ approach to 8000 artificial samples (~8062 cells) in a space of 11 dimensions, grouped into six clusters with standard deviation 1. AMD_i_ reaches the highest value when the number of user-defined clusters is equal to the number of artificial clusters (six). This shows that the AMD_i_ approach allows determining the number of clusters that actually exist in a multidimensional space. The curve depicted in Supplementary Fig. 2c was obtained the same way as in Fig. 2b, but using artificial clusters generated with a standard deviation 1.7 instead of 1 (less well-defined). As expected, AMD_i_ also reaches the highest value when the number of user-defined clusters is 6, but the highest value of AMD_i_ decreases from 0.6 to 0.39. Therefore, the AMD_i_ approach not only allows determining the number of clusters that actually exist, but also estimates their degree of definition. Supplementary Fig. 2d shows the curve resulting from applying the AMD_i_ approach to the 11-dimensional trophic space defined by the selected 8062 cells with low human impact. The peak identifies six clusters, reaching AMD_i_ of almost 0.4, equivalent to that obtained in Supplementary Fig. 2c with artificial samples and standard deviation 1.7. The pattern with real samples (Supplementary Fig. 2d) is not exactly like the one obtained with artificial samples (Supplementary Fig. 2c). There is a secondary peak with 2 clusters, which might arise because the 6 groups are not independent like the artificial ones, but part of two larger groups. Because of the many samples and dimensions in our data, there are many possible clustering solutions, so we obtained 200 replicates for each user-defined number of clusters and the solution with highest AMD_i_ was selected.

Once the existence and number of distinct configurations of trophic structures was demonstrated and counted using the AMD_i_ approach, the same 8062 cells with low human impact were classified into one of the six basic structures using the same clustering method. Supplementary Fig. 3a shows their world distribution. Colour intensity is related to the degrees of membership of the cells with regard to its cluster. Fuzzy clustering was performed using R statistical software, with the package “e1071”^50^.

Each of the 10,356 cells not used to identify the trophic structures, owing to their higher human impact, was subsequently assigned one of the six trophic structures identified using Random forests (RFs). RFs^58^ are powerful tools to classify large amounts of data. They are able to deal with unbalanced data, do not expect linear features or even features that interact linearly, and can handle high-dimensional spaces. RFs have few parameters to tune and the default ones were used. Once the 18,418 cells of the world were assigned to one of the six food web architectures, they were mapped (Fig. 2a).

### Relationship between trophic structures and species richness

In order to examine the contribution of species richness to differences among community trophic structures, we fitted a principal components analysis (PCA). We extracted its axes representing the variation in trophic structures and related the axes with species richness. Specifically, the PCA was performed on a matrix with numbers of 11 guilds (as variables) by the 8062 geographical cells used to identify the six trophic structures (Supplementary Fig. 3c).

The first axis of the PCA explains 50% of the total variance in trophic structures. Its scores are inversely related to the total number of species in each cell: low scores coincide with species-rich cells, and high scores coincide with species-poor cells. Species richness explains 98% of the variance in the first axis of PCA (*r*^2^ = 0.98). Two major groups emerge from the first PCA axis: the first group includes three species-poor trophic structures (depauperate, boreal, temperate); the second includes three species-rich trophic structures (semi-arid, seasonal tropical, humid tropical) (Fig. 2a, Supplementary Fig. 3b, 3c, 4). While the differentiation between species-rich and species-poor structures is strongly related to the first axis of PCA, differences in trophic structures within the two groups are only slightly related to species richness (Supplementary Fig. 3c). It is the second axis of the PCA that accounts for such differentiation. PCA axis 2 explains 16% of the total variance. Being orthogonal do PCA 1, it is independent of it and thus independent of species richness. The third component explains 9% but is not related to further differentiation among trophic structures.

### Representing results as energetic networks in Fig. 1

Trophic structures, once identified, were represented as bipartite energetic networks^59^ to visually represent differences in the relative contributions of guilds and resources (Fig. 1). These bipartite energetic networks were obtained using Gephi 0.8.2 beta^60^.

### Objective 3: climatic modelling of trophic structures

The relationship between the six trophic structures and climate was modelled using evolutionary learning of globally optimal classification trees^61^. Models were calibrated using the 6730 mainland cells out of the 8062 cells with relatively low human impact (see above). Islands grid cells were excluded because mapping of the six trophic structures (Fig. 2a) revealed that they do not follow any climatic pattern on islands, being generally depauperate.

In classification trees, the decision threshold is determined by testing all possible thresholds and choosing the one that maximize the chosen measure of homogeneity. In recursive classification trees, these splits are chosen without consideration of the nodes further down the tree, hence yielding only locally optimal trees. Globally optimal classification trees search the full parameter space of trees using evolutionary algorithms^62^, which is a method inspired by the process of natural selection. Analyses were performed using R statistical software with the package “evtree”^63^. The evtree package allows constraining the complexity of the resulting tree by limiting the minimum number of samples in a terminal leave or the maximum depth of the tree. The goal was to find a tree model that, involving the minimum number of variables and terminal groups, allows for bioclimatic characterization of the six trophic structures. Such a parsimonious approach enhances interpretation of inferred relationships while avoiding overfitting. The resulting tree model was applied to the rest of the terrestrial cells of the world in order to obtain the expected trophic structure according to their climate.

### Objective 4: determining human impacts on trophic structures

Comparison of the observed and predicted distribution of trophic structures (Fig. 2a, b) shows consistent disagreements. Our starting hypothesis was that the disagreements were related to human impacts. Fig. 4a shows (I) depauperate structures whose climatic prediction is boreal or temperate, (II) semi-arid structures whose climatic prediction is either seasonal or humid tropical, and (III) islands being classified as depauperate independently of their climatic conditions. To test if disagreements I and II are related to human activities, their relationship with six human impact indicators was analysed. These included the percentage of five land cover types present in each grid cell^42^ (i.e., cropland, pasture, primary vegetation, secondary vegetation, and urban land) and human population density^43^. First, we used a Student's *t* test to compare the mean value of individual human impact indicator in changed and unchanged cells. Differences are significant (<10^−2^) for all the indicators and for both type I and II disagreements (Supplementary Table 1). In all cases but two, differences are important and significant (<10^−15^). The percentage of surface covered by primary vegetation is more than double in unchanged cells (i.e., where observed structures match predicted ones) in both types of disagreement. The percentage of surface covered by pasture is also higher in unchanged cells for both types of disagreement. Secondary vegetation is higher in changed cells for disagreement of type I and unchanged cells for disagreement type II. The percentage of area covered by agricultural and urban land, as well as the human population, is more than double in the changed cells. Human impact indicators are correlated, so classification trees globally optimized with evolutionary algorithms were also used to search for an explanatory model relating the human impact indicators with disagreements I and II (Fig. 4).

### Reporting summary

Further information on research design is available in the Nature Research Reporting Summary linked to this article.

## Supplementary information

Supplementary Information

Reporting Summary

Description of Additional Supplementary Files

Supplementary Data 1

Supplementary Data 2

## Data Availability

The authors declare that all food preferences data supporting the findings of this study are available within the paper and its Supplementary Information files.
