# Supplementary material for: Climate shapes mammal community trophic structures and humans simplify them
Source: Nat Commun. 2019 Nov 15;10:5197. doi: 10.1038/s41467-019-12995-9 (PMC6858300; doi:10.1038/s41467-019-12995-9)
Supplement: Supplementary file 1 — Supplementary Information [file 41467_2019_12995_MOESM1_ESM.pdf]

Supplementary information

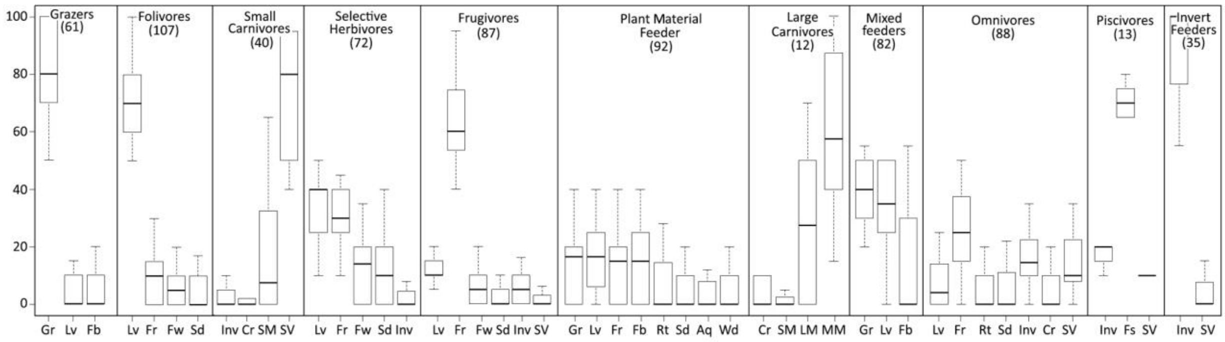

Supplementary Figure 1 | Dispersion of the estimated percentage of each of 19 trophic resources in the diet of the species grouped in each trophic guild. Trophic resources: grass Gr, leaves Lv, fruits Fr, flowers Fw, lichens Lch, forbes Fb, moss Ms, roots Rt, seeds Sd, fungi Fg, aquatic plants Aq, wood and bark Wd, invertebrates Inv, fish Fs, carrion Cr, small vertebrates SV, mammals 1-10kg SM, mammals 10-100 kg MM, mammals >100 kg LM (only those resources which upper whisker is higher than zero are included); trophic guilds (values in parentheses indicate the number of species).

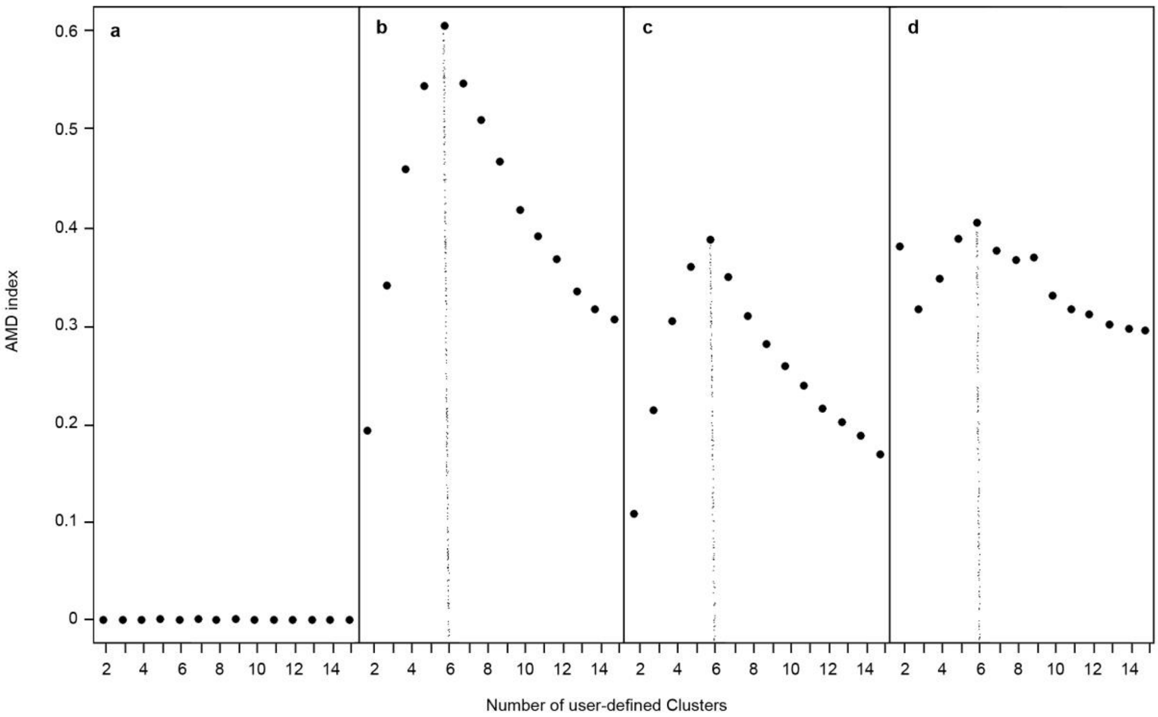

Supplementary Figure 2 | Changes in the AMD index through a series of clustering analysis runs with increasing number of previously-defined clusters. a, with 8000 artificial samples randomly distributed in an 11-dimensional space of side 10; **b**, with 8000 artificial samples grouped in 6 clusters with standard deviation 1; **c**, with standard deviation 1.7; **d**, with the 11-dimensional trophic space defined by the trophic profile of the mammalian assemblages of the 8062 cells with low human impact

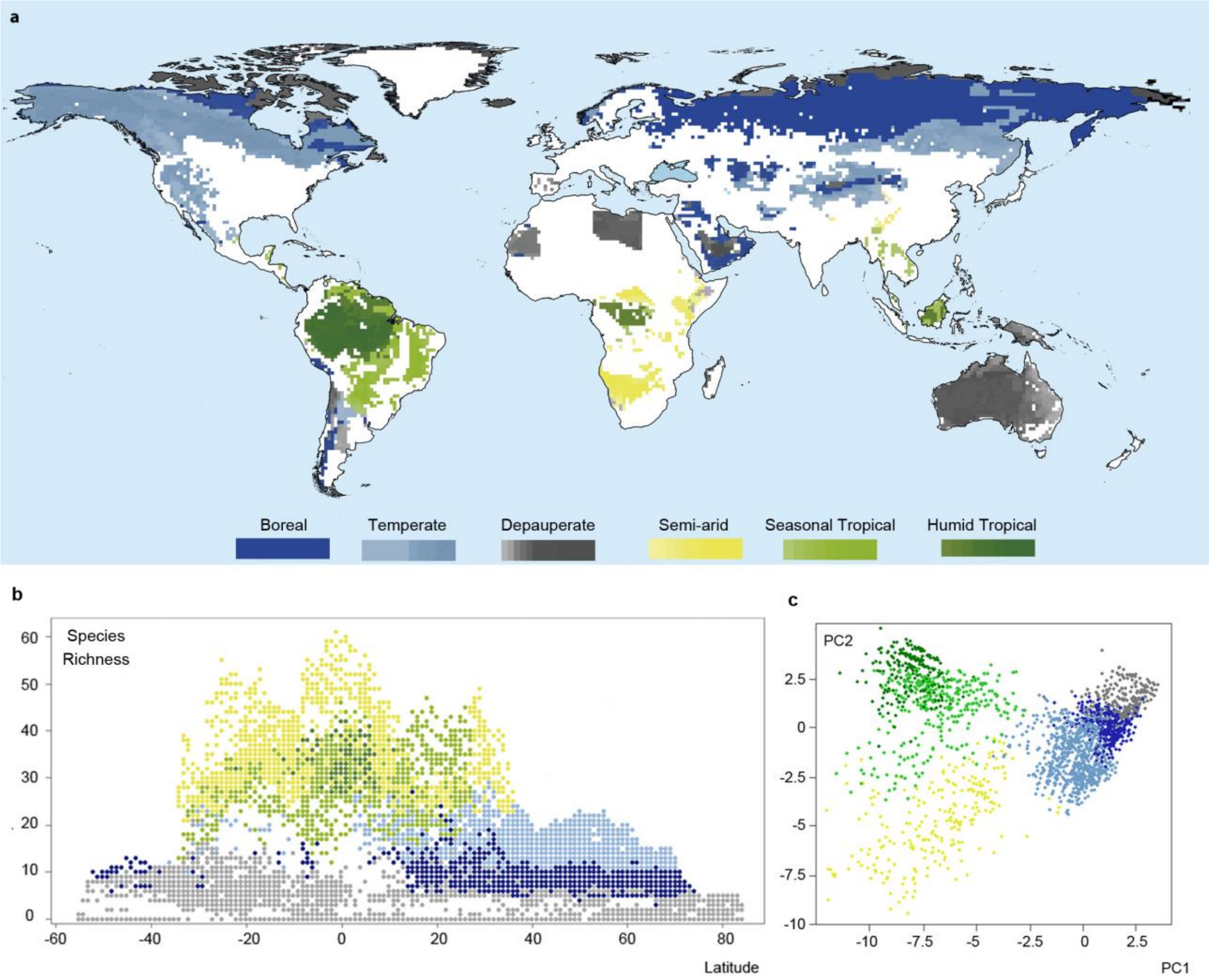

Supplementary Figure 3 | Characteristics of the 8062 lower human impact grid cells used to identify the six emerging trophic structures. a, world distribution (colour intensity is related with the fuzzy degrees of membership of the cell to its cluster, see text). **b**, trophic structures plotted against latitude and species richness. **c**, Projection on the trophic space defined by the two fist principal components.

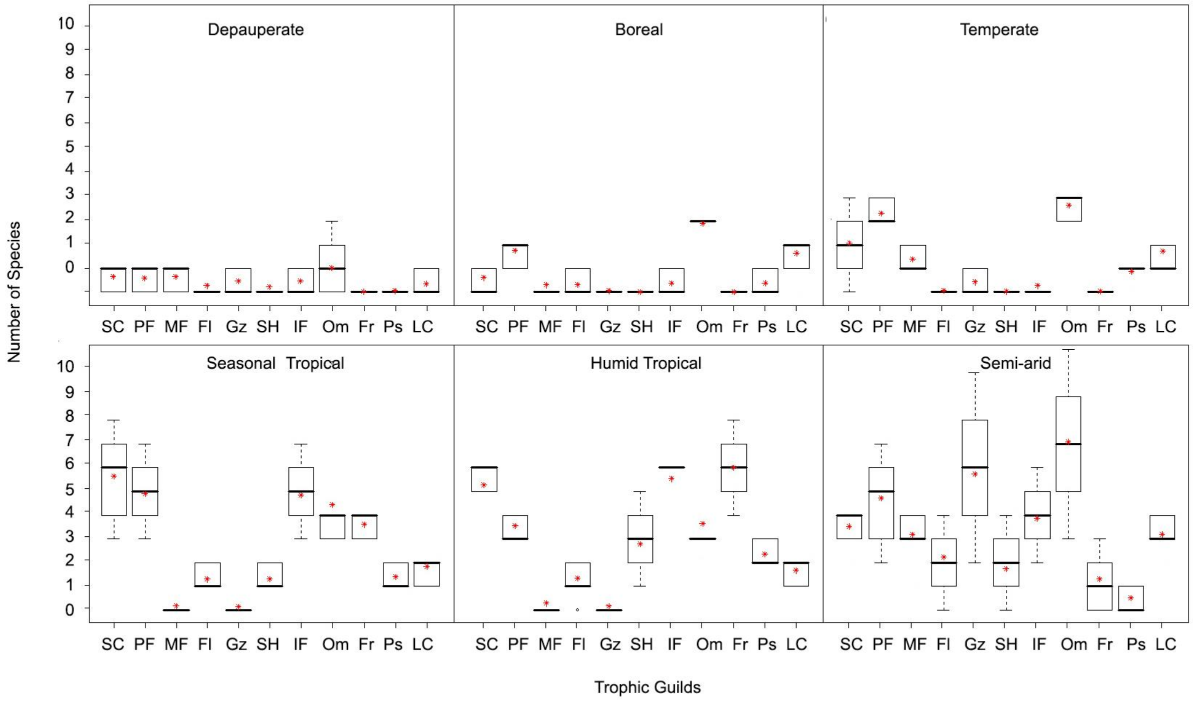

Supplementary Figure 4 | Distribution of the number of species from each trophic guild grouped in each type of trophic structure. Trophic guilds: small carnivores SC, plant material feeders PF, mixed feeders MF, folivores Fl, grazers Gz, selective herbivores SH, invertebrate feeders IF, omnivores Om, frugivores Fv, piscivores Ps, large carnivores LC, Red symbol: mean value.

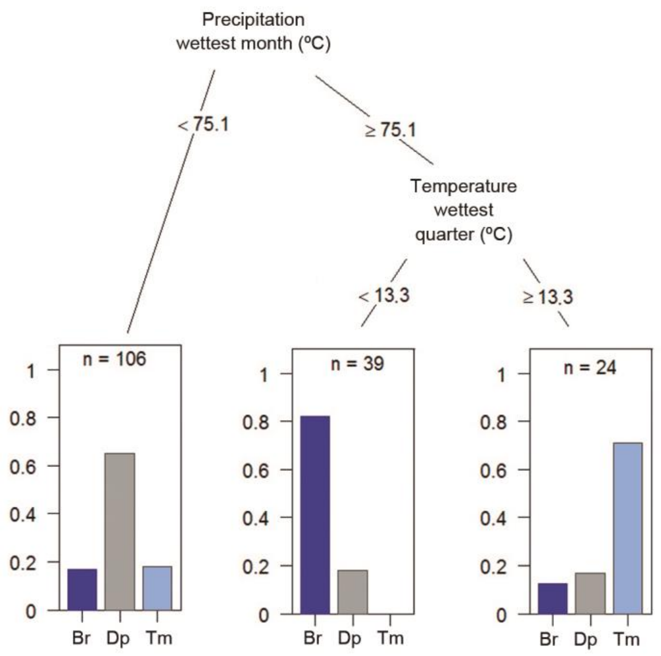

Supplementary Figure 5 | Tree model, extension of tree in figure 3. It relates climate predictors and trophic structures in those cells with annual mean temperature lower than 17.6 mm and isothermality above 46.8 (leaf F, Fig. 3, see text).

.

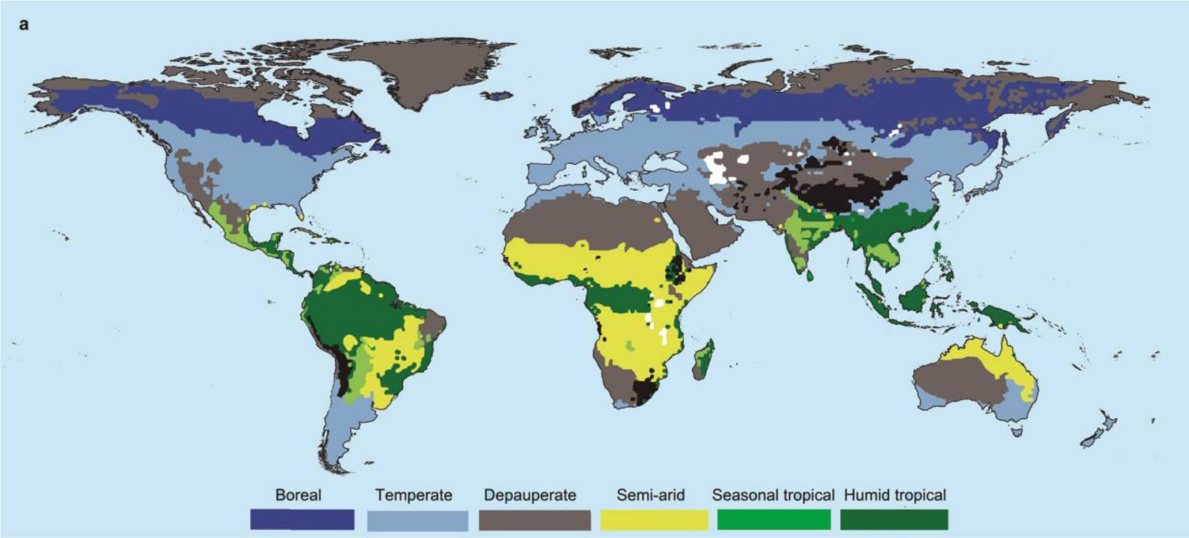

b

Biome (Olson & Dinerstein, 2002)	Biome
Boreal Forests/Taiga	boreal (Br)
Temperate Broadleaf and Mixed Forests	temperate (Tm)
Temperate Conifer Forests	temperate (Tm)
Temperate Grasslands, Savannas and Shrublands	temperate (Tm)
Mediterranean Forests, Woodlands and Scrub	temperate (Tm)
Tropical and Subtropical Coniferous Forests	seasonal tropical (ST)
Tropical and Subtropical Dry Broadleaf Forests	seasonal tropical (ST)
Mangroves	seasonal tropical (ST)
Tropical and Subtropical Moist Broadleaf Forests	Humid tropical (HT)
Tropical and Subtropical Grasslands, Savannas and Shrublands	Semi-arid (SA)
Flooded Grasslands and Savannas	Semi-arid (SA)
Rock and Ice	depauperate (Dp)
Deserts and Xeric Shrublands	depauperate (Dp)
Tundra	depauperate (Dp)

Supplementary Figure 6 | Equivalence between terrestrial biomes and trophic structures. a, world distribution of the terrestrial biomes aggregated according to the table in a. For consistency, the colour scheme follows that of the trophic structures in Figure 2. Black areas correspond to montane habitats that were not
.
individualized by the trophic structure classification and white areas are inland waters. **b**, this table shows the equivalence between the terrestrial biomes as established by Olson & Dinerstein (2002) for WWF / Global 200 and their aggregation into 6 broad categories for comparison with trophic structures.

Supplementary Table 1 | Human impact differences associated to changes in emerging trophic structures.

Impact indicator	Disagreement type I			Disagreement type I		
	Depauperate (1107)	Non-depauperate (9401)	p-value	Tropical to Semi-arid (1020)	Equal (245)	p-value
Primary Vegetation (%)	22	45	$< 2.2e^{-16}$	13	30	$< 2.2e^{-16}$
Secondary Vegetation (%)	14	21	$< 2.2e^{-16}$	37	11	$< 2.2e^{-16}$
Pasture (%)	14	16	$9.3e^{-4}$	28	46	$< 2.2e^{-16}$
Cropland (%)	21	7	$< 2.2e^{-16}$	18	8	$7.39e^{-15}$
Urban (%)	1.3	0.2	$< 2.2e^{-16}$	0.17	0.09	$5.7e^{-3}$
Human Pop (hab/Km^2)	83	30	$< 2.2e^{-16}$	89	26	$< 2.2e^{-16}$

Disagreement type I, Depauperate = mean values for those cells with boreal or temperate trophic structures as bioclimatic projection that actually have depauperate structure; Non-depauperate = mean values for those cells which observed trophic structure and bioclimatic projection structure is either temperate or boreal.

Disagreement type II Tropical to Semiarid = mean values for those cells that actually have semiarid trophic structure and its climatic potential is humid or seasonal tropical; Equal = mean values for those cells which current and potential trophic structure is semiarid
