# Supplementary material for: Climate shapes mammal community trophic structures and humans simplify them
Source: Nat Commun. 2019 Nov 15;10:5197. doi: 10.1038/s41467-019-12995-9 (PMC6858300; doi:10.1038/s41467-019-12995-9)
Supplement: Supplementary file 3 — Description of Additional Supplementary Files [file 41467_2019_12995_MOESM3_ESM.pdf]

Description of Additional Supplementary Files

File Name: Supplementary Data 1
Description: Supplementary References

File Name: Supplementary Data 2
Description: Estimates of feeding preferences of large mammals based on analysis of supplementary references
