# Supplementary material for: Climate shapes mammal community trophic structures and humans simplify them
Source: Nat Commun. 2019 Nov 15;10:5197. doi: 10.1038/s41467-019-12995-9 (PMC6858300; doi:10.1038/s41467-019-12995-9)
Supplement: Supplementary file 4 — Supplementary Data 1 [file 41467_2019_12995_MOESM4_ESM.pdf]

Supplementary Data File 1: References from which the feeding habits of the large mammals were obtained

1. Aranda, M., & Sánchez-Cordero, V. (1996). Prey spectra of jaguar (*Panthera onca*) and puma (Puma concolor) in tropical forests of Mexico. *Studies on Neotropical Fauna and Environment*, *31*(2), 65-67.
2. Asquith, N., J. Terbogh, A. Arnold, C. Riveros. 1999. The fruits the agouti ate: Hymenaea courbaril seed fate when its disperser is absent.. *Journal of Tropical Ecology*, 15: 229-235.
3. Atkinson, R. P. D., Macdonald, D. W., & Kamizola, R. (2002). Dietary opportunism in side-striped jackals *Canis adustus Sundevall*. *Journal of Zoology*, *257*(1), 129-139.
4. Austin, D., P. Urness, 1995 *Wild Ungulate Depredation on Winter Wheat: Effects on Wheat Yield.* (On-line). Accessed December 10, 2013 at http://digitalcommons.unl.edu/cgi/viewcontent.cgi?article=1421&context=gpwdcwp.
5. Bagchi, S., Goyal, S. P., & Sankar, K. (2003). Prey abundance and prey selection by tigers (*Panthera tigris*) in a semi-arid, dry deciduous forest in western India.*Journal of Zoology*, *260*(3), 285-290.
6. Barreto, G. R., Hernandez, O. E., & Ojasti, J. (1997). Diet of peccaries (*Tayassu tajacu* and *T. pecari*) in a dry forest of Venezuela. *Journal of Zoology*, *241*(2), 279-284.
7. Beck-King, H., O. von Helversen, R. Beck-King. 1999. Home Range, Population Density, and Food Resources of *Agouti paca* (Rodentia: Agoutidae) in Costa Rica: A Study Using Alternative Methods. *Biotropica*, 31/4: 675-685.
8. Benefit, B. R. (2000). Old World monkey origins and diversification: an evolutionary study of diet and dentition. *Old World Monkeys*, 133.
9. Bhat, S.D., and Rawat, G.S. (1999). Some food plants of chital (*Axis axis*) in Rajaji National Park, India. *Journal of the Bombay Natural History Society* **96**(3): 467-468.
10. Bhowmik, M.K., Chakraborty, T., and Raha, A.K. (1999). The habitat and food habits of hog deer (*Axis porcinus*) in protected areas of sub-Himalayan West Bengal. *Tiger Paper* **26**(2): 25-27.
11. Bisbal E, F. J. (1986). Food habits of some neotropical carnivores in Venezuela (Mammalia, Carnivora). *Mammalia*, *50*(3), 329-339.
12. Bruno, E., & Riccardi, C. (1995). The diet of the Crested porcupine *Hystrix cristata* L., 1758 in a Mediterranean rural area. *Zeitschrift für Säugetierkunde*, *60*(4), 226-236.
13. Call, J. 2004. Inferences about the location of food in the Great Apes (*Pan paniscus*, *Pan trogodytes*, *Gorilla gorilla*, and *Pongo pygmaeus*). *Journal of Comparative Psychology*, 118: 232-241. Accessed August 15, 2013 at http://www.cs.arizona.edu/projects/wonac/papers/Call2004JCP.pdf.
14. Canadian Museum of Nature, 2003. "African Wild Dog" (On-line). Natural History Notebooks. Accessed 05/24/13 at http://nature.ca/notebooks/english/afwildog.htm
15. Capitani, C., Bertelli, I., Varuzza, P., Scandura, M., & Apollonio, M. (2004). A comparative analysis of wolf (*Canis lupus*) diet in three different Italian ecosystems. *Mammalian Biology-Zeitschrift für Säugetierkunde*, *69*(1), 1-10.
16. Carbyn, L., T. Trotter. 1988. Descriptions of Wolf Attacks on Bison Calves in Wood Buffalo National Park. *Arctic*, 41: 297-302.
17. Carter, T., U. Mochi. 1971. *Hoofed Mammals of the World*. New York: Charles Scribner's Sons.
18. Cavallini, P., & Volpi, T. (1996). Variation in the diet of the red fox in a Mediterranean area. *Revue d'écologie*, *51*(2), 173-189.
19. Cerling, T. E., Harris, J. M., & Passey, B. H. (2003). Diets of East African Bovidae based on stable isotope analysis. *Journal of Mammalogy*, *84*(2), 456-470.

20. Cerling, T.E., and Viehl, K. (2004). Seasonal diet changes of the forest hog (*Hylochoerus meinertzhageni* Thomas) based on the carbon isotopic composition of hair. *African Journal of Ecology* **42**(2): 88-92.

21. Chapman, C. A., Chapman, L. J., & Gillespie, T. R. (2002). Scale issues in the study of primate foraging: red colobus of Kibale National Park. *American Journal of Physical Anthropology*, *117*(4), 349-363.

22. Chapple, R.S. (1989). *The biology and behaviour of chital deer (*Axis axis*) in captivity*. PhD Thesis, University of Sydney, NSW, Australia.

23. Chemes, S. B., Giraudo, A. R., & Gil, G. (2010). Dieta de *Lontra Longicaudis* (Carnivora, Mustelidae) en el Parque Nacional El Rey (Salta, Argentina) y su comparación con otras poblaciones de la cuenca del Paraná. *Mastozoología neotropical*, *17*(1), 19-29.

24. Chiarello, A. G. (1998). Diet of the Atlantic forest maned sloth *Bradypus torquatus* (Xenarthra: Bradypodidae). *Journal of Zoology*, *246*(1), 11-19.

25. Chillo, V., D. Rodriguez, R. Ojeda. 2010. Niche partitioning and coexistence between two mammalian herbivores in the Dry Chaco of Argentina. *Acta Oecologica*, 36/6: 611-616.

26. Clevenger, A. P., Purroy, F. J., & Pelton, M. R. (1992). Food habits on brown bears (*Ursus arctos*) in the Cantabrian Mountain, Spain. *Journal of Mammalogy*, *73*(2), 415-421.

27. Copley, P. B., & Robinson, A. C. (1983). Studies on the Yellow-Footed Rock-Wallaby, *Petrogale Xanthopus* Gray (Marsupialia: Macropodidae). 2. Diet. *Wildlife Research*, *10*(1), 63-76.

28. Codron, D., Codron, J., Lee-Thorp, J. A., Sponheimer, M., De Ruiter, D., Sealy, J., & Fourie, N. (2007). Diets of savanna ungulates from stable carbon isotope composition of faeces. *Journal of Zoology*, *273*(1), 21-29.

29. Coombes, K. E. (2005). *The ecology and utilisation of Lumholtz's tree kangaroos Dendrolagus lumholtzi (Marsupialia: Macropodidae), on the Atherton Tablelands, far north Queensland* (Doctoral dissertation, James Cook University).

30. Cristóbal-Azkarate, J., & Arroyo-Rodríguez, V. (2007). Diet and activity pattern of howler monkeys (Alouatta palliata) in Los Tuxtlas, Mexico: effects of habitat fragmentation and implications for conservation. *American Journal of Primatology*,*69*(9), 1013-1029.

31. Dasilva, G. L. (1994). Diet ofColobus polykomos on Tiwai Island: Selection of food in relation to its seasonal abundance and nutritional quality. *International Journal of Primatology*, *15*(5), 655-680.

32. de Garine-Wichatitsky, M., Duncan, P., Labbe, A., Suprin, B., Chardonnet, P., & Maillard, D. (2003). A review of the diet of Rusa Deer Cervus timorensis russa in New Caledonia: Are the endemic plants defenceless against this introduced, eruptive ruminant?. *Pacific Conservation Biology*, *9*(2), 136.

33. de Garine-Wichatitsky, M., Soubeyran, Y., Maillard, D., & Duncan, P. (2005). The diets of introduced rusa deer (Cervus timorensis russa) in a native sclerophyll forest and a native rainforest of New Caledonia. *New Zealand Journal of Zoology*, *32*(2), 117-126.

34. Delany, M., D. Happold. 1979. *Ecology of African Mammals*. New York: Longman Group Limited.

35. Dhungel, S.K., and O'Gara, B.W. (1991). The ecology of the hog deer in Royal Chitwan National Park, Nepal. *Wildlife Monographs* (119): 1-40.

36. Diller, H., T. Haltenorth. 1980. *The Collins Field Guide to the Mammals of Africa including Madagascar*. Lexington, Massachusetts: The Stephen Greene Press.

37. Dirks, W. (2003). Effect of diet on dental development in four species of catarrhine primates. American Journal of Primatology, 61(1), 29-40.

38. Donadio, E., & Buskirk, S. W. (2006). Diet, morphology, and interspecific killing in Carnivora. *The American Naturalist*, *167*(4), 524-536.

39. Dorst, J. 1969. *A Field Guide to The Larger Mammals of Africa*. Great Britain: Houghton Mifflin Company Boston.

40. Downer, C. C. (2001). Observations on the diet and habitat of the mountain tapir (Tapirus pinchaque). *Journal of Zoology*, *254*(3), 279-291.

41. Dubost, G. 1984. Comparison of the diets of frugivorous forest mammals of Gabon. Journal of Mammalogy; 65(2): 298-316.

42. Dunston, N., M. Gorman. 1998. *Behavior and Ecology of Riparian Mammals*. Cambridge: Cambridge University Press.

43. Edwards, G. P., Dawson, T. J., & Croft, D. B. (1995). The dietary overlap between red kangaroos (Macropus rufus) and sheep (Ovis aries) in the arid rangelands of Australia. *Australian journal of ecology*, *20*(2), 324-334.

44. Eisenberg, J. F., and K. H. Redford. Mammals of the Neotropics. Chicago: The University of Chicago Press, 1999.

45. Elder, A. A. (2009). Hylobatid diets revisited: The importance of body mass, fruit availability, and interspecific competition. In *The Gibbons* (pp. 133-159). Springer New York.

46. Elliott, H.W. III, and Barrett, R.H. (1986). Dietary overlap among axis *Axis axis axis*, fallow *Dama dama dama*, and black-tailed deer *Odocoileus hemionus columbianus*, and cattle *Bos taurus*. *Journal of Range Management* **38**(6): 546-550.

47. Ellis, B. A., Dawson, T. J., & Tierney, P. J. (1992). The diet of the bridled nailtail wallaby (*Onychogalea fraenata*), 1. Site and seasonal influences and dietary overlap with the black-striped wallaby (Macropus dorsalis) and domestic cattle [central Queensland]. *Wildlife Research*, *19*.

48. Eltringham, S. 1979. The Ecology and Conservation of Large African Mammals. New York: The Macmillan Press Limited.

49. Emmons, L. H. (1987). Comparative feeding ecology of felids in a neotropical rainforest. *Behavioral Ecology and Sociobiology*, *20*(4), 271-283.

50. Engqvist, A., & Richard, A. (1991). Diet as a possible determinant of cathemeral activity patterns in primates. *Folia Primatologica*, *57*(3), 169-172.

51. Estes, R. 1991. The Behavior Guide to African Mammals. Los Angeles: The University of California Press.

52. Estrada, A., & Coates-Estrada, R. (1984). Fruit eating and seed dispersal by howling monkeys (Alouatta palliata) in the tropical rain forest of Los Tuxtlas, Mexico.*American Journal of Primatology*, *6*(2), 77-91.

53. Everitt, J. H., Gonzalez, C. L., Alaniz, M. A., & Latigo, G. V. (1981). Food habits of the collared peccary on south Texas rangelands. *Journal of Range Management*, 141-144.

54. Farrell, L. E., Roman, J., & Sunquist, M. E. (2000). Dietary separation of sympatric carnivores identified by molecular analysis of scats. *Molecular Ecology*, *9*(10), 1583-1590.

55. Feer, F. (1989). Comparaison des régimes alimentaires de *Cephalophus callipygus* et *C. dorsalis*, Bovidés sympatriques de la forêt sempervirente africaine. *Mammalia* **53**(4): 563-620.

56. Feldhamer, G., D. Drickamer, S. Vessey, J. Merritt, C. Krajewski. 2007. *Mammology: Adaptation, Diversity, and Ecology Third Edition*. Baltimore, MD: Johns Hopkins University Press.

57. Fortin, J. K., Farley, S. D., Rode, K. D., & Robbins, C. T. (2007). Dietary and spatial overlap between sympatric ursids relative to salmon use. *Ursus*, *18*(1), 19-29.

58. Fox, M.W. ed. 1975. "The Wild Canids: Their Systematics, Behavioral Ecology and Evolution". Van Nostrand Reinhold Co. New York, NY.

59. Fiorenza, P. 1983. *Encyclopedia of Big Game Animals of Africa*. New York City, New York, USA: Larousse and Co. Inc..

60. Flueck, W. T. 2003. Consideraciones acerca de la calidad nutritiva de hábitat, hábitat óptimo, y evaluación de hábitat para huemul. *In* 4ta reunión Chileno-Argentina sobre estrategias de conservación del huemul. *Edited by* G. Acosta-Jamett. CONAF and CODEFF, Las Trancas, Chile. pp. 30-34.

61. Gade-Jrrgensen, I., & Stagegaard, R. (2000). Diet composition of wolves Canis lupus in east-central Finland. *Zeszyty Problemowe Postepow Nauk Rolniczych*,*45*(4), 537-547.

62. Gagnon, M., & Chew, A. E. (2000). Dietary preferences in extant African Bovidae.*Journal of Mammalogy*, *81*(2), 490-511.

63. Gautier-Hion, A., L. H. Emmons, and G.Dubost. 1980. A comparison of the diets of three major groups of primary consumers of Gabon (primates, squirrels and ruminants). Oecologia (Berlin); 45: 182-189.

64. Gayot, M. 2004. Comparative diet of the two forest cervids of the genus Mazama in French Guiana. *Journal of Tropical Ecology*, 20: 31-43.

65. Gebert, C., & Verheyden-Tixier, H. (2001). Variations of diet composition of red deer (*Cervus elaphus L*.) in Europe. *Mammal Review*, *31*(3‐4), 189-201.

66. Geelen, L. J. (1999). *A preliminary study of the black-footed rock-wallaby (Petrogale lateralis MacDonnell Ranges race) in the Anangu Pitjantjatjara lands, South Australia* (Doctoral dissertation, University of Adelaide).

67. Giannatos, G., Karypidou, A., Legakis, A., & Polymeni, R. (2010). Golden jackal (< i> Canis aureus</i> L.) diet in Southern Greece. *Mammalian Biology-Zeitschrift für Säugetierkunde*, *75*(3), 227-232.

68. Godfrey, L. R., Semprebon, G. M., Jungers, W. L., Sutherland, M. R., Simons, E. L., & Solounias, N. (2004). Dental use wear in extinct lemurs: evidence of diet and niche differentiation. *Journal of Human Evolution*, *47*(3), 145-169.

69. Grajales-Tam, K. M., Rodríguez-Estrella, R., & Cancino-Hernández, J. (2003). Dieta estacional del coyote Canis latrans durante el periodo 1996-1997 en el desierto de vizcaíno, Baja California sur, México. *Acta Zoológica Mexicana*, *89*, 17-28.

70. Grassman, L. I., Tewes, M. E., Silvy, N. J., & Kreetiyutanont, K. (2005). Spatial ecology and diet of the dhole Cuon alpinus (Canidae, Carnivora) in north central Thailand. *Mammalia mamm*, *69*(1), 11-20.

71. Green, M. J. B. 1987. Some ecological aspects of a Himalayan population of musk deer. *In* Biology and Management of the Cervidae. *Edited by* C. M. Wemmer. Washington, D. C.: Smithsonian Institution Press. pp. 307-319.

72. Hackmann, T. (2008). *Studies of ruminant digestion, ecology, and evolution*(Doctoral dissertation, University of Missouri--Columbia).

73. Halford, D. A., Bell, D. T., & Loneragan, W. A. (1984). Diet of the western grey kangaroo (Macropus fuliginosus Desm.) in a mixed pasture-woodland habitat of Western Australia. *Journal of the Royal Society of Western Australia*.

74. Happold, D.C.D. (1987). *The Mammals of Nigeria*. Clarendon Press, Oxford.

75. Harmsen, B., R. Foster, S. Silver, L. Ostro, C. Doncaster. 2011. Jaguar and puma activity patterns in relation to their main prey. *Mammalian Biology*, 76: 320-324.

76. Hart, J. A. (1992). Forage selection, forest availability, and use of space by Okapi (*Okapia johnstoni*) a rainforest giraffe in Zaire. Ongules/Ungulates 91: 217-221.

77. Hayward, M. W., & Kerley, G. I. (2005). Prey preferences of the lion (Panthera leo).*Journal of Zoology*, *267*(3), 309-322.

78. Heise-Pavlov, S. R., Jackrel, S. L., & Meeks, S. (2011). Conservation of a rare arboreal mammal: habitat preferences of the Lumholtz's tree-kangaroo, Dendrolagus lumholtzi. *Australian Mammalogy*, *33*(1), 5-12.

79. Henry, O., Feer, F., & Sabatier, D. (2000). Diet of the Lowland Tapir (Tapirus terrestris L.) in French Guiana1. *Biotropica*, *32*(2), 364-368.

80. Herrera, E., D. Macdonald. 1989. Resource Utilization and Territoriality in Group-Living Capybaras (Hydrochoerus hydrochaeris). *Journal of Animal Ecology*, 58:2: 667-679.

81. Hill, D. A. (1997). Seasonal variation in the feeding behavior and diet of Japanese macaques (Macaca fuscata yakui) in lowland forest of Yakushima. *American Journal of Primatology*, *43*(4), 305-320.

82. Hladik, C. M. (1979). Diet and ecology of prosimians. *The study of prosimian behavior*, 307-357.

83. Homolka, M., & Heroldová, M. (1992). Similarity of the results of stomach and faecal contents analyses in studies of the ungulate diet. *Folia Zoologica*, *41*(3), 193-208.

84. Hutchins M., D Kleiman, V Geist, M McDade, eds. *Grzimek's Animal Life Encylopedia*, Vol. 16, 2nd Edition. Farmington Hills, MI: Gale Group.

85. Hylander, W. L. (1975). Incisor size and diet in anthropoids with special reference to Cercopithecidae. *Science*, *189*(4208), 1095-1098.

86. Irby, L. 1977. Food habits of Chanler's mountain reedbuck in a rift valley ranch. *East African Wildlife Journal*, 15/4: 289-294.

87. Irlbeck, N. A., & Hume, I. D. (2003). The role of Acacia in the diets of Australian marsupials? A review. *Australian Mammalogy*, *25*(2), 121-134.

88. Jackson, J. E. 1987. Ozotoceros bezoarticus. Mammalian Species 295:1-5.

89. Jaeger, M. M., Haque, E., Sultana, P., & Bruggers, R. L. (2007). Daytime cover, diet and space-use of golden jackals (Canis aureus) in agro-ecosystems of Bangladesh.*Mammalia*, *71*(1/2), 1-10.

90. Jarman, M. 1979. Impala Social Behaviour: Territory, Hierarchy, Mating,and the Use of Space. Berlin: Verlag Paul Parey.

91. Jarman, P. J. (1994). The eating of seedheads by species of Macropodidae.*Australian Mammalogy*, *17*, 51-63.

92. Johnsingh, A.J.T., and Sankar, K. (1991). Food plants of chital, sambar, and cattle on Mundanthurai Plateau, Tamil Nadu, South India. *Mammalia* **55**(1): 57-66.

93. Julliot, C., & Sabatier, D. (1993). Diet of the red howler monkey (Alouatta seniculus) in French Guiana. *International Journal of Primatology*, *14*(4), 527-550.

94. Kassim, H., and Baharin, K. (1979). Grazing behaviour of the swamp buffalo (*Bubalus bubalis*). *Pertanika* **2**(2): 125-127.

95. Khan, J.A. (1994). Food habits of ungulates in dry tropical forests of Gir Lion Sanctuary, Gujarat, India. *Acta Theriologica* **39**(2): 185-193.

96. Kielland, K., J. Bryant. 1998. Moose herbivory in taiga: effects on biogeochemistry and vegetation dynamics in primary succession. *Oikos*, 82: 377-383.

97. King C., ed. *The Handbook of New Zealand Mammals*. Auckland: Oxford University Press.

98. Kotwal, P.C., and Mishra, R.P. (2004). Ecobiology of Indian Wild Buffalo *Bubalus arnee* L. in Udanti Wildlife Sanctuary, Chhattisgarh, India. *Journal of the Bombay Natural History Society* **101**(2): 252-254.

99. Kotze, D. C., & Zacharias, P. J. K. (1993). Utilization of woody browse and habitat by the black rhino (Diceros bicornis) in western Itala Game Reserve. *African Journal of Range & Forage Science*, *10*(1), 36-40.

100. Krishnamani, R. (1994). Diet composition of the bonnet macaque (Macaca radiata) in a tropical dry evergreen forest of southern India. *Tropical Biodiversity*, *2*(2), 285-302.

101. Krishnan, M. (1972). An ecological survey of the larger mammals of peninsular India.*Journal of the Bombay Natural History Society* **69**(3): 469-501.

102. Lad, S. (2012). Diet and Incisor Surface Curvature in Cercopithecids.

103. Landa, A., Strand, O., Swenson, J. E., & Skogland, T. (1997). Wolverines and their prey in southern Norway. *Canadian Journal of Zoology*, *75*(8), 1292-1299.

104. Lanszki, J., Kormendi, S., Hancz, C., & Zalewski, A. (1999). Feeding habits and trophic niche overlap in a Carnivora community of Hungary. *Acta Theriologica*,*44*(4), 429-442.

105. Lapidge, S. J., & Henshall, S. (2001). Diet Of Foxes And Cats, With Evidence Of Predation On Yellow-Footed Rock-Wallabies (Petrogale Xanthopus Celeris) By Foxes In Southwsetern Queensland. *Australian Mammalogy*, *23*(1), 47-52.

106. Laska, M., J. Luna Baltazar, E. Rodriguez Luna. 2003. Food preferences and nutrient composition in captive pacas, Agouti Paca (Rodentia, Dasyproctidae). *Mammal Biology*, 68: 31-41.

107. Leighton, M., and Leighton, D.R. (1983). Vertebrate responses to fruiting seasonality within a Bornean rain forest. Pp. 181-196 in: Sutton, S.L., Whitmore, T.C. and Chadwick, A.C. (eds.). *Tropical Rain Forest: Ecology and Management*. Blackwell, Oxford.

108. Lindenmayer, D. B. (1997). Differences in the biology and ecology of arboreal marsupials in forests of southeastern Australia. *Journal of Mammalogy*, 1117-1127.

109. Lucherini, M., & Crema, G. (1994). Seasonal variation in diet and trophic niche of the red fox in an Alpine habitat. *Zeitschrift für Säugetierkunde*, *59*(1), 1-8.

110. Kingdon, J. 1997. *The Kingdon Field Guide to African Mammals*. San Diego, CA: Academic Press.

111. López-Soto, J. H., Henández, R. E. G., & Badii, M. H. Dieta invernal del Coyote (*Canis latrans*) en un rancho del noreste de México.

112. McArthur, C., & Sanson, G. D. (1988). Tooth wear in eastern grey kangaroos (*Macropus giganteus*) and western grey kangaroos (*Macropus fuliginosus*), and its potential influence on diet selection, digestion and population parameters. *Journal of Zoology*, *215*(3), 491-504.

113. Macdonald, D. 1984. *The Encyclopedia of Mammals*. New York NY: Facts on File Publications.

114. Mahaney, W. C. 1987. Behaviour of the African buffalo on Mount Kenya. African Journal of Ecology 25: 199-202.

115. Malcolm, J. 1997. The diet of the Ethiopian wolf (*Canis simensis Ruppell)* from a grassland area of the Bale Mountains, Ethiopia. African Journal of Ecology 35: 162-164.

116. Marassi, M., & Biancardi, C. (2002). Diet of the Eurasian badger (*Meles meles*) in an area of the Italian Prealps. *Hystrix, the Italian Journal of Mammalogy*, *13*(1-2).

117. Meagher, M. (16 June 1986). "*Bison bison*." Mammalian Species. The American Society of Mammalogists, 266.

118. Medel, R., F. Jaksic. 1988. Ecología de los cánidos sudamericanos: una revisión. *Revista Chilena de Historia Natural*, 61: 67-79.

119. Mendoza, M., Janis, C. M., & Palmqvist, P. (2002). Characterizing complex craniodental patterns related to feeding behaviour in ungulates: a multivariate approach. *Journal of Zoology*, *258*(2), 223-246.

120. Mendoza, M., & Palmqvist, P. (2005). Characterizing adaptive morphological patterns related to diet in Bovidae (Mammalia: Artiodactyla). *Dong wu xue bao.[Acta zoologica Sinica]*, *52*(6), 988-1008.

121. Mendoza, M., & Palmqvist, P. (2008). Hypsodonty in ungulates: an adaptation for grass consumption or for foraging in open habitat?. *Journal of Zoology*, *274*(2), 134-142.

122. Mloszewski, M. 1983. The Behaviour and Ecology of the African Buffalo. Cambridge University Press. U.S.A.

123. Moreno, R. (2008). Información preliminar sobre la dieta de jaguares y pumas en Cana, Parque Nacional Darién, Panamá.

124. Morse, P. E., Daegling, D. J., McGraw, W. S., & Pampush, J. D. (2013). Dental wear among cercopithecid monkeys of the Taï forest, Côte d'Ivoire. *American journal of physical anthropology*, *150*(4), 655-665.

125. Mudappa, D., Kumar, A., & Chellam, R. Diet and fruit choice of the brown palm civet Paradoxurus jerdoni, a viverrid endemic to the Western Ghats rainforest, India [282-300].

126. Mukherjee, S., Goyal, S. P., Johnsingh, A. J. T., & Pitman, M. R. P. (2004). The importance of rodents in the diet of jungle cat (Felis chaus), caracal (Caracal caracal) and golden jackal (Canis aureus) in Sariska Tiger Reserve, Rajasthan, India. *Journal of Zoology*, *262*(4), 405-411.

127. Muñoz, A. H. (1991). Dieta de *Felis concolor* (Carnivora: Felidae) en áreas silvestres protegidas del sur de Chile. *Revista Chilena de Historia Natural*, *64*, 139-144.

128. Muya, S. M., & Oguge, N. O. (2000). Effects of browse availability and quality on black rhino (Diceros bicornis michaeliGroves 1967) diet in Nairobi National Park, Kenya. *African Journal of Ecology*, *38*(1), 62-71.

129. Nowak, R. M. (2005). *Walker's Marsupials of the World*. JHU Press.

130. Nowak, R. 1999. *Walker's Mammals of the World (Sixth Edition)*. Baltimore and London: Johns Hopkins University Press.

131. Oates, J. F. (1978). Water-plant and soil consumption by guereza monkeys (Colobus guereza): a relationship with minerals and toxins in the diet?. *Biotropica*, 241-253.

132. O'Brien, T. G., & Kinnaird, M. F. (1997). Behavior, diet, and movements of the Sulawesi crested black macaque (Macaca nigra). *International Journal of Primatology*, *18*(3), 321-351.

133. O'Gara, B. 1978. Antilocapra americana. *Mammalian Species*, 90: 1-7.

134. Oli, M. K., Taylor, I. R., & Rogers, D. M. (1993). Diet of the snow leopard (Panthera uncia) in the Annapurna Conservation Area, Nepal. *Journal of Zoology*, *231*(3), 365-370.

135. Oliver, W.L.R. (1979). Observations of the biology of the pigmy hog (with a footnote on the hispid hare): pigmy hog survey report, part II. *Journal of the Bombay Natural History Society* **76**(2): 115-142.

136. Ott, T., Kerley, G. I., & Boshoff, A. F. (2007). Preliminary observations on the diet of leopards (Panthera pardus) from a conservation area and adjacent rangelands in the Baviaanskloof region, South Africa. *African Zoology*, *42*(1), 31-37.

137. Owen-Smith, N. 1997. Distinctive Features of the Nutritional Ecology of Browsing Versus Grazing Ruminants. *Zeitschrift fuer Saeugetierkunde*, 62: 176-191.

138. Palombit, R. A. (1997). Inter-and intraspecific variation in the diets of sympatric siamang (Hylobates syndactylus) and lar gibbons (Hylobates lar). *Folia Primatologica*, *68*(6), 321-337.

139. Parker S., ed. Grzimek's Encyclopedia of Mammals, Vol. 5, 1 Edition. New York: McGraw-Hill Publishing Company.

140. Pavelka, M. S., & Knopff, K. H. (2004). Diet and activity in black howler monkeys (Alouatta pigra) in southern Belize: does degree of frugivory influence activity level?.*Primates*, *45*(2), 105-111.

141. Pedó, E., Tomazzoni, A. C., Hartz, S. M., & Christoff, A. U. (2006). Diet of crab-eating fox, Cerdocyon thous (Linnaeus)(Carnivora, Canidae), in a suburban area of southern Brazil. *Revista Brasileira de Zoologia*, *23*(3), 637-641.

142. Pigozzi, G., & Patterson, I. J. (1990). Movements and diet of crested porcupines in the Maremma natural-park, central Italy. *Acta Theriologica*, *35*(3-4), 173-180.

143. Pombo, A. R., Waltert, M., Mansjoer, S. S., Mardiastuti, A., & Mühlenberg, M. (2004). Home range, diet and behaviour of the Tonkean macaque (Macaca tonkeana) in Lore Lindu National Park, Sulawesi. In *Land use, nature conservation and the stability of rainforest margins in southeast asia* (pp. 313-325). Springer Berlin Heidelberg.

144. Poulle, M. L., Carles, L., & Lequette, B. (1997). Significance of ungulates in the diet of recently settled wolves in the Mercantour mountains (southeastern France).*Revue d'écologie*, *52*(4), 357-368.

145. Prins, H. 1996. *Ecology and Behaviour of the African Buffalo*. Great Britain: Chapman and Hall.

146. Puig, S., M. Cona, F. Videla, E. Mendez. 2010. Diet of the mara (Dolichotis patagonum), food availability and effects of an extended drought in Northern Patagonia (Mendoza, Argentina). *Mammalian Biology*, 75/5: 389-398.

147. Quadros, J., & Monteiro-Filho, E. L. (2001). Diet of the neotropical otter, Lontra longicaudis, in an Atlantic forest area, Santa Catarina State, southern Brazil.*Studies on Neotropical fauna and Environment*, *36*(1), 15-21.

148. Rau, J. R., & Jiménez, J. E. (2002). Diet of puma (Puma concolor, Carnivora: Felidae) in coastal and Andean ranges of southern Chile. *Studies on Neotropical Fauna and Environment*, *37*(3), 201-205.

149. Richardson, P. R. K. (1987). Food consumption and seasonal variation in the diet of the aardwolf Proteles cristatus in southern Africa. *Zeitschrift für Säugetierkunde*,*52*(5), 307-325.

150. Riley, E. P. (2007). Flexibility in diet and activity patterns of Macaca tonkeana in response to anthropogenic habitat alteration. *International Journal of Primatology*,*28*(1), 107-133.

151. Ryan, A. S. (1981). Anterior dental microwear and its relationship to diet and feeding behavior in three african primates (Pan troglodytes troglodytes, Gorilla gorilla gorilla andPapio hamadryas). *Primates*, *22*(4), 533-550.

152. Rice, C. G. 1988. Notes on the food habits of Nilgiri tahr. Journal of the Bombay Natural History Society; 85: 188-189.

153. Robinson, M. 2005. The Arabian tahr: A review of its biology and conservation. Caprinae (Newsletter of the IUCN/SSC Caprinae Specialist Group; October 2005: 2-4.

154. Rodriguez, D., M. Ana Dacar. 2008. Diet composition of the mara (Dolichotis patagonum) in the southeast of the Monte desert of La Pampa province, Argentina.. Mastozoologia Neotropical, 15/2: 215-220.

155. Roper, T. J., & Lüps, P. (1995). Diet of badgers (Meles meles) in central Switzerland: an analysis of stomach contents. *Zeitschrift für Säugetierkunde*, *60*(1), 9-19.

156. Salas, L. A., & Fuller, T. K. (1996). Diet of the lowland tapir (Tapirus terrestris L.) in the Tabaro River valley, southern Venezuela. *Canadian Journal of Zoology*, *74*(8), 1444-1451.

157. Sánchez, F., Gómez-Valencia, B., Álvarez, S. J., & Gómez-Laverde, M. (2008). Primeros datos sobre los hábitos alimentarios del tigrillo, Leopardus pardalis, en un bosque andino de Colombia. *Revista UDCA Actualidad & Divulgación Científica*,*11*(2), 101-107.

158. Saragih, W; E. V. I., Justina Sadsoeitoeboen, M & Pattiselanno, F. (2010). The diet of spotted cuscus (*Spilocuscus maculatus*) in natural and captivity habitat. *Nusantara Bioscience*, *2*(2).

159. Short, J. (1989). The diet of the brush-tailed rock-wallaby in New-South-Wales.*Wildlife Research*, *16*(1), 11-18.

160. Schaller, G. May 1996. Realm of the Snow Antelope. New York: American Museum of Natural History.

161. Schaller, G. 1998. Wildlife of the Tibetan Steppe. Chicago: University of Chicago Press.

162. Semiadil, G., Barry, T. N., Muir, P. D., & Hodgson, J. (1995). Dietary preferences of sambar (Cervus unicolor) and red deer (Cervus elaphus) offered browse, forage legume and grass species. *The Journal of Agricultural Science*, *125*(01), 99-107.

163. Shepherdson, D. J., Roper, T. J., & Lüps, P. (1990). Diet, food availability and foraging behaviour of badgers (Meles meles L.) in southern England. *Zeitschrift für Säugetierkunde*, *55*(2), 81-93.

164. Sillero-Zubiri, C., & Gottelli, D. (1995). Diet and feeding behavior of Ethiopian wolves (Canis simensis). *Journal of Mammalogy*, *76*(2), 531-541.

165. Simmen, B., Bayart, F., Marez, A., & Hladik, A. (2007). Diet, nutritional ecology, and birth season of Eulemur macaco in an anthropogenic forest in Madagascar.*International Journal of Primatology*, *28*(6), 1253-1266.

166. Smith, S. 1985. *The Atlas of Africa's Principle Mammals*. Sandton: Natural History Books.

167. Shukla, R., and Khare, P.K. (1998). Food habits of wild ungulates and their competition with livestock in Pench Wildlife Reserve, central India. *Journal of the Bombay Natural History Society* **95**(3): 418-421.

168. Sombra, M., A. Mangione. 2005. Obsessed with grasses? The case of mara Dolichotis patagonum (Caviidae : Rodentia). Revista Chilena de Historia Natural, 78/3: 401-408.

169. Sowls, L. 1997. *Javelinas and other Peccaries: Their Biology, Management, and Use: Second Edition*. Arizona: Texas A & M University Press.

170. Spencer, L. M. (1995). Morphological correlates of dietary resource partitioning in the African Bovidae. *Journal of Mammalogy*, 448-471.

171. Spinage, C. 1986. The Natural History of Antelopes. New York, NY, USA: Facts On File Publications.

172. Sponheimer, M., Reed, K. E., & Lee-Thorp, J. A. (1999). Combining isotopic and ecomorphological data to refine bovid paleodietary reconstruction: a case study from the Makapansgat Limeworks hominin locality. *Journal of Human Evolution*,*36*(6), 705-718.

173. Sprent, J. A., & McArthur, C. (2002). Diet and diet selection of two species in the macropodid browser–grazer continuum—do they eat what they'should'?. *Australian Journal of Zoology*, *50*(2), 183-192.

174. Stafford, K. J. (1997). The diet and trace element status of sambar deer (Cervus unicoloi) in Manawatu district, New Zealand. *New Zealand Journal of Zoology*,*24*(4), 267-271.

175. Stoen, O.G., and Wegge, P. (1996). Prey selection and prey removal by tiger (*Panthera tigris*) during the dry season in lowland Nepal. *Mammalia* **60**(3): 363-373.

176. Stuart, C. and T. Stuart. 1995. *Stuart's Field Guide to the Mammals of Southern Africa*. Cape Town: Struik.

177. Stewart, K. M., Bowyer, R. T., Kie, J. G., Dick, B. L., & Ben-David, M. (2003). Niche partitioning among mule deer, elk, and cattle: Do stable isotopes reflect dietary niche?. *Ecoscience*, *10*(3), 297-302.

178. Taber, A. B., Novaro, A. J., Neris, N., & Colman, F. H. (1997). The food habits of sympatric jaguar and puma in the Paraguayan Chaco. *Biotropica*, *29*(2), 204-213.

179. Tan, C. L. (1999). Group composition, home range size, and diet of three sympatric bamboo lemur species (genus Hapalemur) in Ranomafana National Park, Madagascar. *International Journal of Primatology*, *20*(4), 547-566.

180. Taylor, R. J. (1983). The diet of the eastern grey kangaroo and wallaroo in areas of improved and native pasture in the New England Tablelands. *Wildlife Research*,*10*(2), 203-211.

181. Taylor, M. E., & Hannam, A. G. (1987). Tooth microwear and diet in the African Viverridae. *Canadian Journal of Zoology*, *65*(7), 1696-1702.

182. Teaford, M. F., & Robinson, J. G. (1989). Seasonal or ecological differences in diet and molar microwear in Cebus nigrivittatus. *American Journal of Physical Anthropology*, *80*(3), 391-401.

183. Texera, W. A. 1974. Some aspects of the biology of the guemal *Hippocamelus bisulcus*Mammalia Artiodactyla Cervidae in captivity. Part 1: Observations on the behavior of the guemal and calculation of some physiological aspects. Anales del Instituto de la Patagonia; 5(1-2): 155-166.

184. Theodor, J. M. (2002). Artiodactyla (Even-Toed Ungulates Including Sheep and Camels). *eLS*.

185. Tobler, M. W. (2002). Habitat Use and Diet of Baird's Tapirs (*Tapirus bairdii*) in a Montane Cloud Forest of the Cordillera de Talamanca, Costa Rica1. *Biotropica*,*34*(3), 468-474.

186. Tomazzoni, A., E. Pedo, S. Hartz. 2005. Feeding associations between capybaras and birds in the Lami Biological Reserve. *Revista Brasileira de Zoologia*, 22:3: 712-716.

187. Turkalo A., J Fay, eds. *Forest Elephant Behavior and Ecology*. New Haven and London: Yale University Press.

188. Urbani, B., & Bosque, C. (2007). Feeding ecology and postural behaviour of the three-toed sloth (< i> Bradypus variegatus flaccidus</i>) in northern Venezuela.*Mammalian Biology-Zeitschrift für Säugetierkunde*, *72*(6), 321-329.

189. Van Dijk, J., Hauge, K., Landa, A., Andersen, R., & May, R. (2007). Evaluating scat analysis methods to assess wolverine Gulo gulo diet. *Wildlife Biology*, *13*(sp2), 62-67.

190. Van Rooyen, A. F. 1992. Diets of impala and nyala in two game reserves in Natal, South Africa. South African Journal of Wildlife Research 22(4): 98-101.

191. Vernes, K. (1995). The diet of the red-legged pademelon Thylogale stigmatica (Gould)(Marsupialia: Macropodidae) in fragmented tropical rainforest, north Queensland, Australia. *Mammalia*, *59*(4), 517-526.

192. Wachter, B., Schabel, M., & Noë, R. (1997). Diet overlap and polyspecific associations of red colobus and Diana monkeys in the Taï National Park, Ivory Coast. *Ethology*, *103*(6), 514-526.

193. Watanabe, K. (1989). Fish: a new addition to the diet of Japanese macaques on Koshima Island. *Folia Primatologica*, *52*(3-4), 124-131.

194. Wegge, P., Shrestha, A.K., and Moe, S.R. (2006). Dry season diets of sympatric ungulates in lowland Nepal: competition and facilitation in alluvial tall grasslands. *Ecological Research* **21**(5): 698-706.

195. Vieira, E. M., & De Moraes, D. A. (2003). Carnivory and insectivory in Neotropical marsupials. *Predators with pouches: the biology of carnivorous marsupials*, 267-280.

196. Whitehead, K. G. 1972. *Deer of the world*. New York, USA: The Viking Press, Inc..

197. Whitehead, K. G. 1993. The Whitehead Encyclopedia of Deer. Stillwater, MN: Voyageur Press, Inc.

198. Wildlife Africa CC, 2002. "Wildlife Africa - Wild Dog Behavior" (On-line). WildlifeAfrica. Accessed September 24, 2013 at http://www.wildlifeafrica.co.za/wildogbehavior.html.

199. Wilson, D., D. Reeder, eds. 1993. Mammal Species of the World. Washington, DC: Smithsonian Institution Press.

200. Woolnough, A. P. (1998). *The feeding ecology of the northern hairy-nosed wombat, Lasiorhinus krefftii (Marsupialia: Vombatidae)* (Doctoral dissertation, James Cook University).

201. Woolnough, A. P., & Johnson, C. N. (2000). Assessment of the potential for competition between two sympatric herbivores-the northern hairy-nosed wombat, Lasiorhinus krefftii, and the eastern grey kangaroo, Macropus giganteus. *Wildlife Research*, *27*(3), 301-308.

202. Zucarotto, R., R. Carrara, S. Franco, B. Karina. 2010. Diet of paca (Cuniculus paca) using indirect methods in an agricultural area in the Brazilian Atlantic Forest. *Biotemas*, 23/1: 235-239.
