# Supplementary material for: Climate shapes mammal community trophic structures and humans simplify them
Source: Nat Commun. 2019 Nov 15;10:5197. doi: 10.1038/s41467-019-12995-9 (PMC6858300; doi:10.1038/s41467-019-12995-9)
Supplement: Supplementary file 5 — Supplementary Data 2 [file 41467_2019_12995_MOESM5_ESM.pdf]

**Supplementary Data file 2: References from which the feeding habits of the large mammals were obtained**

| Species | Gr | Lv | Fr | Fw | Lc | Fb | Ms | Rt | Sd | Aq | Wd | Fg | In | Fs | Cr | SV | SM | MM | LM | TG |
|---|---|---|---|---|---|---|---|---|---|---|---|---|---|---|---|---|---|---|---|---|
| *Antilocapra Americana* | 20 | 20 | 0 | 0 | 0 | 0 | 0 | 30 | 0 | 0 | 30 | 0 | 0 | 0 | 0 | 0 | 0 | 0 | 0 | GH |
| *Addax nasomaculatus* | 20 | 20 | 0 | 0 | 0 | 40 | 0 | 0 | 20 | 0 | 0 | 0 | 0 | 0 | 0 | 0 | 0 | 0 | 0 | GH |
| *Aepyceros melampus* | 50 | 20 | 10 | 0 | 0 | 0 | 0 | 0 | 0 | 0 | 20 | 0 | 0 | 0 | 0 | 0 | 0 | 0 | 0 | MF |
| *Alcelaphus buselaphus* | 90 | 10 | 0 | 0 | 0 | 0 | 0 | 0 | 0 | 0 | 0 | 0 | 0 | 0 | 0 | 0 | 0 | 0 | 0 | Gr |
| *Alcelaphus caama* | 90 | 10 | 0 | 0 | 0 | 0 | 0 | 0 | 0 | 0 | 0 | 0 | 0 | 0 | 0 | 0 | 0 | 0 | 0 | Gr |
| *Alcelaphus lichtensteinii* | 100 | 0 | 0 | 0 | 0 | 0 | 0 | 0 | 0 | 0 | 0 | 0 | 0 | 0 | 0 | 0 | 0 | 0 | 0 | Gr |
| *Ammodorcas clarkei* | 0 | 100 | 0 | 0 | 0 | 0 | 0 | 0 | 0 | 0 | 0 | 0 | 0 | 0 | 0 | 0 | 0 | 0 | 0 | Fl |
| *Ammotragus lervia* | 30 | 40 | 0 | 0 | 30 | 0 | 0 | 0 | 0 | 0 | 0 | 0 | 0 | 0 | 0 | 0 | 0 | 0 | 0 | MF |
| *Antidorcas marsupialis* | 50 | 50 | 0 | 0 | 0 | 0 | 0 | 0 | 0 | 0 | 0 | 0 | 0 | 0 | 0 | 0 | 0 | 0 | 0 | MF |
| *Antilope cervicapra* | 60 | 10 | 10 | 10 | 0 | 0 | 0 | 0 | 10 | 0 | 0 | 0 | 0 | 0 | 0 | 0 | 0 | 0 | 0 | Gr |
| *Beatragus hunteri* | 100 | 0 | 0 | 0 | 0 | 0 | 0 | 0 | 0 | 0 | 0 | 0 | 0 | 0 | 0 | 0 | 0 | 0 | 0 | Gr |
| *Bison bison* | 60 | 0 | 0 | 0 | 20 | 0 | 20 | 0 | 0 | 0 | 0 | 0 | 0 | 0 | 0 | 0 | 0 | 0 | 0 | Gr |
| *Bison bonasus* | 60 | 10 | 0 | 0 | 10 | 0 | 10 | 0 | 0 | 0 | 10 | 0 | 0 | 0 | 0 | 0 | 0 | 0 | 0 | Gr |
| *Bos grunniens* | 30 | 10 | 0 | 0 | 10 | 10 | 10 | 0 | 0 | 30 | 0 | 0 | 0 | 0 | 0 | 0 | 0 | 0 | 0 | MF |
| *Bos javanicus* | 20 | 30 | 20 | 0 | 0 | 30 | 0 | 0 | 0 | 0 | 0 | 0 | 0 | 0 | 0 | 0 | 0 | 0 | 0 | GH |
| *Bos sauveli* | 30 | 30 | 0 | 0 | 0 | 0 | 0 | 40 | 0 | 0 | 0 | 0 | 0 | 0 | 0 | 0 | 0 | 0 | 0 | MF |
| *Boselaphus tragocamelus* | 20 | 20 | 20 | 20 | 0 | 0 | 0 | 0 | 20 | 0 | 0 | 0 | 0 | 0 | 0 | 0 | 0 | 0 | 0 | SF |
| *Bubalus depressicornis* | 20 | 30 | 20 | 0 | 0 | 0 | 0 | 0 | 0 | 30 | 0 | 0 | 0 | 0 | 0 | 0 | 0 | 0 | 0 | GH |
| *Bubalus mindorensis* | 100 | 0 | 0 | 0 | 0 | 0 | 0 | 0 | 0 | 0 | 0 | 0 | 0 | 0 | 0 | 0 | 0 | 0 | 0 | Gr |
| *Bubalus quarlesi* | 50 | 50 | 0 | 0 | 0 | 0 | 0 | 0 | 0 | 0 | 0 | 0 | 0 | 0 | 0 | 0 | 0 | 0 | 0 | MF |
| *Budorcas taxicolor* | 50 | 50 | 0 | 0 | 0 | 0 | 0 | 0 | 0 | 0 | 0 | 0 | 0 | 0 | 0 | 0 | 0 | 0 | 0 | MF |
| *Capra caucasica* | 50 | 50 | 0 | 0 | 0 | 0 | 0 | 0 | 0 | 0 | 0 | 0 | 0 | 0 | 0 | 0 | 0 | 0 | 0 | MF |
| *Capra falconeri* | 50 | 50 | 0 | 0 | 0 | 0 | 0 | 0 | 0 | 0 | 0 | 0 | 0 | 0 | 0 | 0 | 0 | 0 | 0 | MF |
| *Capra hircus* | 50 | 50 | 0 | 0 | 0 | 0 | 0 | 0 | 0 | 0 | 0 | 0 | 0 | 0 | 0 | 0 | 0 | 0 | 0 | MF |
| *Capra ibex* | 50 | 50 | 0 | 0 | 0 | 0 | 0 | 0 | 0 | 0 | 0 | 0 | 0 | 0 | 0 | 0 | 0 | 0 | 0 | MF |
| *Capra nubiana* | 50 | 50 | 0 | 0 | 0 | 0 | 0 | 0 | 0 | 0 | 0 | 0 | 0 | 0 | 0 | 0 | 0 | 0 | 0 | MF |
| *Capra pyrenaica* | 20 | 70 | 0 | 0 | 0 | 10 | 0 | 0 | 0 | 0 | 0 | 0 | 0 | 0 | 0 | 0 | 0 | 0 | 0 | Fl |
| *Capra sibirica* | 50 | 50 | 0 | 0 | 0 | 0 | 0 | 0 | 0 | 0 | 0 | 0 | 0 | 0 | 0 | 0 | 0 | 0 | 0 | MF |

| | | | | | | | | | | | | | | | | | | | | |
|---|---|---|---|---|---|---|---|---|---|---|---|---|---|---|---|---|---|---|---|---|
| *Capra walie* | 40 | 40 | 0 | 0 | 20 | 0 | 0 | 0 | 0 | 0 | 0 | 0 | 0 | 0 | 0 | 0 | 0 | 0 | 0 | MF |
| *Capricornis crispus* | 0 | 60 | 0 | 0 | 0 | 0 | 0 | 0 | 40 | 0 | 0 | 0 | 0 | 0 | 0 | 0 | 0 | 0 | 0 | Fl |
| *Capricornis milneedwardsii* | 0 | 100 | 0 | 0 | 0 | 0 | 0 | 0 | 0 | 0 | 0 | 0 | 0 | 0 | 0 | 0 | 0 | 0 | 0 | Fl |
| *Capricornis rubidus* | 50 | 50 | 0 | 0 | 0 | 0 | 0 | 0 | 0 | 0 | 0 | 0 | 0 | 0 | 0 | 0 | 0 | 0 | 0 | MF |
| *Capricornis sumatraensis* | 0 | 100 | 0 | 0 | 0 | 0 | 0 | 0 | 0 | 0 | 0 | 0 | 0 | 0 | 0 | 0 | 0 | 0 | 0 | Fl |
| *Capricornis swinhoei* | 50 | 50 | 0 | 0 | 0 | 0 | 0 | 0 | 0 | 0 | 0 | 0 | 0 | 0 | 0 | 0 | 0 | 0 | 0 | MF |
| *Capricornis thar* | 50 | 50 | 0 | 0 | 0 | 0 | 0 | 0 | 0 | 0 | 0 | 0 | 0 | 0 | 0 | 0 | 0 | 0 | 0 | MF |
| *Cephalophus adersi* | 0 | 50 | 50 | 0 | 0 | 0 | 0 | 0 | 0 | 0 | 0 | 0 | 0 | 0 | 0 | 0 | 0 | 0 | 0 | Fr |
| *Cephalophus brookei* | 0 | 20 | 30 | 20 | 0 | 0 | 0 | 0 | 30 | 0 | 0 | 0 | 0 | 0 | 0 | 0 | 0 | 0 | 0 | SF |
| *Cephalophus callipygus* | 0 | 16 | 80 | 2 | 0 | 0 | 0 | 0 | 0 | 0 | 0 | 2 | 0 | 0 | 0 | 0 | 0 | 0 | 0 | Fr |
| *Cephalophus dorsalis* | 0 | 5 | 40 | 0 | 0 | 0 | 0 | 0 | 40 | 0 | 0 | 5 | 5 | 0 | 0 | 5 | 0 | 0 | 0 | Om |
| *Cephalophus jentinki* | 0 | 80 | 20 | 0 | 0 | 0 | 0 | 0 | 0 | 0 | 0 | 0 | 0 | 0 | 0 | 0 | 0 | 0 | 0 | Fl |
| *Cephalophus leucogaster* | 0 | 40 | 40 | 20 | 0 | 0 | 0 | 0 | 0 | 0 | 0 | 0 | 0 | 0 | 0 | 0 | 0 | 0 | 0 | SF |
| *Cephalophus natalensis* | 0 | 40 | 40 | 20 | 0 | 0 | 0 | 0 | 0 | 0 | 0 | 0 | 0 | 0 | 0 | 0 | 0 | 0 | 0 | SF |
| *Cephalophus niger* | 0 | 40 | 40 | 20 | 0 | 0 | 0 | 0 | 0 | 0 | 0 | 0 | 0 | 0 | 0 | 0 | 0 | 0 | 0 | SF |
| *Cephalophus nigrifrons* | 0 | 20 | 80 | 0 | 0 | 0 | 0 | 0 | 0 | 0 | 0 | 0 | 0 | 0 | 0 | 0 | 0 | 0 | 0 | Fr |
| *Cephalophus ogilbyi* | 0 | 20 | 30 | 20 | 0 | 0 | 0 | 0 | 30 | 0 | 0 | 0 | 0 | 0 | 0 | 0 | 0 | 0 | 0 | SF |
| *Cephalophus rufilatus* | 0 | 70 | 15 | 15 | 0 | 0 | 0 | 0 | 0 | 0 | 0 | 0 | 0 | 0 | 0 | 0 | 0 | 0 | 0 | Fl |
| *Cephalophus silvicultor* | 20 | 20 | 20 | 0 | 0 | 0 | 0 | 0 | 20 | 0 | 10 | 10 | 0 | 0 | 0 | 0 | 0 | 0 | 0 | GH |
| *Cephalophus spadix* | 0 | 10 | 30 | 20 | 0 | 20 | 20 | 0 | 0 | 0 | 0 | 0 | 0 | 0 | 0 | 0 | 0 | 0 | 0 | Om |
| *Cephalophus weynsi* | 0 | 40 | 40 | 20 | 0 | 0 | 0 | 0 | 0 | 0 | 0 | 0 | 0 | 0 | 0 | 0 | 0 | 0 | 0 | SF |
| *Cephalophus zebra* | 0 | 35 | 35 | 0 | 0 | 30 | 0 | 0 | 0 | 0 | 0 | 0 | 0 | 0 | 0 | 0 | 0 | 0 | 0 | SF |
| *Connochaetes gnou* | 100 | 0 | 0 | 0 | 0 | 0 | 0 | 0 | 0 | 0 | 0 | 0 | 0 | 0 | 0 | 0 | 0 | 0 | 0 | Gr |
| *Connochaetes taurinus* | 100 | 0 | 0 | 0 | 0 | 0 | 0 | 0 | 0 | 0 | 0 | 0 | 0 | 0 | 0 | 0 | 0 | 0 | 0 | Gr |
| *Damaliscus korrigum* | 100 | 0 | 0 | 0 | 0 | 0 | 0 | 0 | 0 | 0 | 0 | 0 | 0 | 0 | 0 | 0 | 0 | 0 | 0 | Gr |
| *Damaliscus lunatus* | 90 | 10 | 0 | 0 | 0 | 0 | 0 | 0 | 0 | 0 | 0 | 0 | 0 | 0 | 0 | 0 | 0 | 0 | 0 | Gr |
| *Damaliscus pygargus* | 100 | 0 | 0 | 0 | 0 | 0 | 0 | 0 | 0 | 0 | 0 | 0 | 0 | 0 | 0 | 0 | 0 | 0 | 0 | Gr |
| *Dorcatragus megalotis* | 0 | 50 | 0 | 0 | 0 | 50 | 0 | 0 | 0 | 0 | 0 | 0 | 0 | 0 | 0 | 0 | 0 | 0 | 0 | Fl |
| *Eudorcas rufifrons* | 70 | 30 | 0 | 0 | 0 | 0 | 0 | 0 | 0 | 0 | 0 | 0 | 0 | 0 | 0 | 0 | 0 | 0 | 0 | Gr |
| *Eudorcas thomsonii* | 35 | 35 | 0 | 0 | 0 | 0 | 0 | 0 | 30 | 0 | 0 | 0 | 0 | 0 | 0 | 0 | 0 | 0 | 0 | MF |
| *Gazella bennettii* | 40 | 10 | 10 | 0 | 0 | 40 | 0 | 0 | 0 | 0 | 0 | 0 | 0 | 0 | 0 | 0 | 0 | 0 | 0 | MF |

| | | | | | | | | | | | | | | | | | | | | |
|---|---|---|---|---|---|---|---|---|---|---|---|---|---|---|---|---|---|---|---|---|
| *Gazella cuvieri* | 35 | 35 | 0 | 0 | 0 | 30 | 0 | 0 | 0 | 0 | 0 | 0 | 0 | 0 | 0 | 0 | 0 | 0 | 0 | MF |
| *Gazella dorcas* | 0 | 40 | 20 | 20 | 0 | 0 | 0 | 0 | 20 | 0 | 0 | 0 | 0 | 0 | 0 | 0 | 0 | 0 | 0 | SF |
| *Gazella gazella* | 35 | 35 | 0 | 0 | 0 | 30 | 0 | 0 | 0 | 0 | 0 | 0 | 0 | 0 | 0 | 0 | 0 | 0 | 0 | MF |
| *Gazella leptoceros* | 35 | 35 | 0 | 0 | 0 | 30 | 0 | 0 | 0 | 0 | 0 | 0 | 0 | 0 | 0 | 0 | 0 | 0 | 0 | MF |
| *Gazella spekei* | 35 | 35 | 0 | 0 | 0 | 30 | 0 | 0 | 0 | 0 | 0 | 0 | 0 | 0 | 0 | 0 | 0 | 0 | 0 | MF |
| *Gazella subgutturosa* | 35 | 35 | 0 | 0 | 0 | 30 | 0 | 0 | 0 | 0 | 0 | 0 | 0 | 0 | 0 | 0 | 0 | 0 | 0 | MF |
| *Hemitragus hylocrius* | 50 | 0 | 0 | 0 | 0 | 50 | 0 | 0 | 0 | 0 | 0 | 0 | 0 | 0 | 0 | 0 | 0 | 0 | 0 | MF |
| *Hemitragus jayakari* | 0 | 40 | 30 | 0 | 0 | 0 | 0 | 0 | 20 | 0 | 10 | 0 | 0 | 0 | 0 | 0 | 0 | 0 | 0 | SF |
| *Hemitragus jemlahicus* | 45 | 45 | 10 | 0 | 0 | 0 | 0 | 0 | 0 | 0 | 0 | 0 | 0 | 0 | 0 | 0 | 0 | 0 | 0 | MF |
| *Hippotragus equinus* | 90 | 10 | 0 | 0 | 0 | 0 | 0 | 0 | 0 | 0 | 0 | 0 | 0 | 0 | 0 | 0 | 0 | 0 | 0 | Gr |
| *Hippotragus niger* | 90 | 10 | 0 | 0 | 0 | 0 | 0 | 0 | 0 | 0 | 0 | 0 | 0 | 0 | 0 | 0 | 0 | 0 | 0 | Gr |
| *Kobus ellipsiprymnus* | 80 | 10 | 0 | 0 | 0 | 0 | 0 | 0 | 0 | 10 | 0 | 0 | 0 | 0 | 0 | 0 | 0 | 0 | 0 | Gr |
| *Kobus kob* | 90 | 10 | 0 | 0 | 0 | 0 | 0 | 0 | 0 | 0 | 0 | 0 | 0 | 0 | 0 | 0 | 0 | 0 | 0 | Gr |
| *Kobus leche* | 90 | 5 | 0 | 0 | 0 | 0 | 0 | 0 | 0 | 5 | 0 | 0 | 0 | 0 | 0 | 0 | 0 | 0 | 0 | Gr |
| *Kobus megaceros* | 70 | 0 | 0 | 0 | 0 | 0 | 0 | 0 | 0 | 30 | 0 | 0 | 0 | 0 | 0 | 0 | 0 | 0 | 0 | Gr |
| *Kobus vardonii* | 100 | 0 | 0 | 0 | 0 | 0 | 0 | 0 | 0 | 0 | 0 | 0 | 0 | 0 | 0 | 0 | 0 | 0 | 0 | Gr |
| *Litocranius walleri* | 2 | 50 | 16 | 16 | 0 | 16 | 0 | 0 | 0 | 0 | 0 | 0 | 0 | 0 | 0 | 0 | 0 | 0 | 0 | Fl |
| *Madoqua guentheri* | 0 | 25 | 25 | 20 | 0 | 20 | 0 | 0 | 10 | 0 | 0 | 0 | 0 | 0 | 0 | 0 | 0 | 0 | 0 | SF |
| *Madoqua kirkii* | 20 | 80 | 0 | 0 | 0 | 0 | 0 | 0 | 0 | 0 | 0 | 0 | 0 | 0 | 0 | 0 | 0 | 0 | 0 | Fl |
| *Madoqua piacentinii* | 0 | 50 | 0 | 0 | 0 | 50 | 0 | 0 | 0 | 0 | 0 | 0 | 0 | 0 | 0 | 0 | 0 | 0 | 0 | Fl |
| *Madoqua saltiana* | 0 | 40 | 20 | 20 | 0 | 20 | 0 | 0 | 0 | 0 | 0 | 0 | 0 | 0 | 0 | 0 | 0 | 0 | 0 | SF |
| *Naemorhedus baileyi* | 30 | 40 | 0 | 0 | 30 | 0 | 0 | 0 | 0 | 0 | 0 | 0 | 0 | 0 | 0 | 0 | 0 | 0 | 0 | MF |
| *Naemorhedus caudatus* | 30 | 40 | 0 | 0 | 30 | 0 | 0 | 0 | 0 | 0 | 0 | 0 | 0 | 0 | 0 | 0 | 0 | 0 | 0 | MF |
| *Naemorhedus goral* | 30 | 20 | 0 | 0 | 10 | 10 | 0 | 10 | 10 | 0 | 0 | 10 | 0 | 0 | 0 | 0 | 0 | 0 | 0 | MF |
| *Naemorhedus griseus* | 0 | 30 | 30 | 10 | 0 | 30 | 0 | 0 | 0 | 0 | 0 | 0 | 0 | 0 | 0 | 0 | 0 | 0 | 0 | SF |
| *Nanger dama* | 30 | 40 | 0 | 0 | 0 | 30 | 0 | 0 | 0 | 0 | 0 | 0 | 0 | 0 | 0 | 0 | 0 | 0 | 0 | MF |
| *Nanger granti* | 0 | 70 | 0 | 0 | 0 | 30 | 0 | 0 | 0 | 0 | 0 | 0 | 0 | 0 | 0 | 0 | 0 | 0 | 0 | Fl |
| *Nanger soemmerringii* | 30 | 40 | 0 | 0 | 0 | 30 | 0 | 0 | 0 | 0 | 0 | 0 | 0 | 0 | 0 | 0 | 0 | 0 | 0 | MF |
| *Neotragus batesi* | 10 | 50 | 0 | 0 | 0 | 10 | 0 | 0 | 0 | 0 | 0 | 30 | 0 | 0 | 0 | 0 | 0 | 0 | 0 | Fl |
| *Neotragus moschatus* | 0 | 30 | 30 | 20 | 0 | 0 | 0 | 0 | 0 | 0 | 0 | 20 | 0 | 0 | 0 | 0 | 0 | 0 | 0 | SF |
| *Neotragus pygmaeus* | 0 | 30 | 30 | 20 | 0 | 0 | 0 | 0 | 0 | 0 | 0 | 20 | 0 | 0 | 0 | 0 | 0 | 0 | 0 | SF |

| | | | | | | | | | | | | | | | | | | | | |
|---|---|---|---|---|---|---|---|---|---|---|---|---|---|---|---|---|---|---|---|---|
| *Oreamnos americanus* | 50 | 50 | 0 | 0 | 0 | 0 | 0 | 0 | 0 | 0 | 0 | 0 | 0 | 0 | 0 | 0 | 0 | 0 | 0 | MF |
| *Oreotragus oreotragus* | 0 | 0 | 50 | 50 | 0 | 0 | 0 | 0 | 0 | 0 | 0 | 0 | 0 | 0 | 0 | 0 | 0 | 0 | 0 | Fr |
| *Oryx beisa* | 30 | 40 | 30 | 0 | 0 | 0 | 0 | 0 | 0 | 0 | 0 | 0 | 0 | 0 | 0 | 0 | 0 | 0 | 0 | MF |
| *Oryx dammah* | 25 | 15 | 15 | 0 | 0 | 30 | 0 | 15 | 0 | 0 | 0 | 0 | 0 | 0 | 0 | 0 | 0 | 0 | 0 | GH |
| *Oryx gazella* | 70 | 30 | 0 | 0 | 0 | 0 | 0 | 0 | 0 | 0 | 0 | 0 | 0 | 0 | 0 | 0 | 0 | 0 | 0 | Gr |
| *Oryx leucoryx* | 50 | 50 | 0 | 0 | 0 | 0 | 0 | 0 | 0 | 0 | 0 | 0 | 0 | 0 | 0 | 0 | 0 | 0 | 0 | MF |
| *Ourebia ourebi* | 50 | 50 | 0 | 0 | 0 | 0 | 0 | 0 | 0 | 0 | 0 | 0 | 0 | 0 | 0 | 0 | 0 | 0 | 0 | MF |
| *Ovibos moschatus* | 50 | 15 | 0 | 0 | 0 | 15 | 10 | 0 | 0 | 10 | 0 | 0 | 0 | 0 | 0 | 0 | 0 | 0 | 0 | MF |
| *Ovis ammon* | 40 | 0 | 0 | 0 | 0 | 30 | 0 | 0 | 0 | 30 | 0 | 0 | 0 | 0 | 0 | 0 | 0 | 0 | 0 | MF |
| *Ovis canadensis* | 70 | 5 | 0 | 0 | 0 | 15 | 0 | 0 | 0 | 10 | 0 | 0 | 0 | 0 | 0 | 0 | 0 | 0 | 0 | Gr |
| *Ovis dalli* | 60 | 0 | 0 | 0 | 0 | 0 | 0 | 0 | 0 | 40 | 0 | 0 | 0 | 0 | 0 | 0 | 0 | 0 | 0 | Gr |
| *Ovis nivicola* | 20 | 0 | 0 | 0 | 20 | 20 | 0 | 0 | 20 | 20 | 0 | 0 | 0 | 0 | 0 | 0 | 0 | 0 | 0 | GH |
| *Ovis aries* | 40 | 40 | 0 | 0 | 0 | 0 | 0 | 0 | 20 | 0 | 0 | 0 | 0 | 0 | 0 | 0 | 0 | 0 | 0 | MF |
| *Pantholops hodgsonii* | 30 | 10 | 0 | 0 | 0 | 30 | 0 | 0 | 0 | 30 | 0 | 0 | 0 | 0 | 0 | 0 | 0 | 0 | 0 | MF |
| *Pelea capreolus* | 10 | 90 | 0 | 0 | 0 | 0 | 0 | 0 | 0 | 0 | 0 | 0 | 0 | 0 | 0 | 0 | 0 | 0 | 0 | Fl |
| *Philantomba maxwellii* | 0 | 30 | 10 | 10 | 0 | 30 | 0 | 14 | 0 | 0 | 0 | 6 | 0 | 0 | 0 | 0 | 0 | 0 | 0 | GH |
| *Philantomba monticola* | 0 | 0 | 40 | 20 | 0 | 0 | 0 | 0 | 0 | 20 | 0 | 15 | 5 | 0 | 0 | 0 | 0 | 0 | 0 | Fr |
| *Procapra gutturosa* | 50 | 0 | 0 | 0 | 0 | 50 | 0 | 0 | 0 | 0 | 0 | 0 | 0 | 0 | 0 | 0 | 0 | 0 | 0 | MF |
| *Procapra picticaudata* | 10 | 0 | 0 | 0 | 0 | 80 | 0 | 0 | 0 | 10 | 0 | 0 | 0 | 0 | 0 | 0 | 0 | 0 | 0 | GH |
| *Procapra przewalskii* | 40 | 10 | 0 | 0 | 0 | 10 | 0 | 0 | 0 | 40 | 0 | 0 | 0 | 0 | 0 | 0 | 0 | 0 | 0 | MF |
| *Pseudois nayaur* | 70 | 15 | 0 | 0 | 0 | 15 | 0 | 0 | 0 | 0 | 0 | 0 | 0 | 0 | 0 | 0 | 0 | 0 | 0 | Gr |
| *Pseudois schaeferi* | 80 | 10 | 0 | 0 | 0 | 0 | 10 | 0 | 0 | 0 | 0 | 0 | 0 | 0 | 0 | 0 | 0 | 0 | 0 | Gr |
| *Pseudoryx nghetinhensis* | 0 | 50 | 0 | 10 | 0 | 40 | 0 | 0 | 0 | 0 | 0 | 0 | 0 | 0 | 0 | 0 | 0 | 0 | 0 | Fl |
| *Raphicerus campestris* | 20 | 40 | 0 | 0 | 0 | 0 | 0 | 40 | 0 | 0 | 0 | 0 | 0 | 0 | 0 | 0 | 0 | 0 | 0 | MF |
| *Raphicerus melanotis* | 20 | 60 | 20 | 0 | 0 | 0 | 0 | 0 | 0 | 0 | 0 | 0 | 0 | 0 | 0 | 0 | 0 | 0 | 0 | Fl |
| *Raphicerus sharpei* | 0 | 70 | 10 | 0 | 0 | 0 | 0 | 20 | 0 | 0 | 0 | 0 | 0 | 0 | 0 | 0 | 0 | 0 | 0 | Fl |
| *Redunca arundinum* | 35 | 5 | 30 | 0 | 0 | 0 | 0 | 0 | 0 | 30 | 0 | 0 | 0 | 0 | 0 | 0 | 0 | 0 | 0 | GH |
| *Redunca fulvorufula* | 100 | 0 | 0 | 0 | 0 | 0 | 0 | 0 | 0 | 0 | 0 | 0 | 0 | 0 | 0 | 0 | 0 | 0 | 0 | Gr |
| *Redunca redunca* | 0 | 10 | 0 | 0 | 0 | 40 | 0 | 0 | 10 | 40 | 0 | 0 | 0 | 0 | 0 | 0 | 0 | 0 | 0 | GH |
| *Rupicapra pyrenaica* | 0 | 20 | 0 | 30 | 10 | 30 | 10 | 0 | 0 | 0 | 0 | 0 | 0 | 0 | 0 | 0 | 0 | 0 | 0 | GH |
| *Rupicapra rupicapra* | 25 | 50 | 0 | 0 | 0 | 0 | 0 | 0 | 0 | 0 | 0 | 25 | 0 | 0 | 0 | 0 | 0 | 0 | 0 | MF |

| | | | | | | | | | | | | | | | | | | | | |
|---|---|---|---|---|---|---|---|---|---|---|---|---|---|---|---|---|---|---|---|---|
| *Saiga tatarica* | 70 | 10 | 0 | 0 | 10 | 10 | 0 | 0 | 0 | 0 | 0 | 0 | 0 | 0 | 0 | 0 | 0 | 0 | 0 | Gr |
| *Sylvicapra grimmia* | 0 | 50 | 10 | 10 | 0 | 0 | 0 | 20 | 0 | 0 | 4 | 0 | 4 | 0 | 0 | 2 | 0 | 0 | 0 | Fl |
| *Syncerus caffer* | 100 | 0 | 0 | 0 | 0 | 0 | 0 | 0 | 0 | 0 | 0 | 0 | 0 | 0 | 0 | 0 | 0 | 0 | 0 | Gr |
| *Taurotragus derbianus* | 20 | 30 | 30 | 0 | 0 | 20 | 0 | 0 | 0 | 0 | 0 | 0 | 0 | 0 | 0 | 0 | 0 | 0 | 0 | GH |
| *Taurotragus oryx* | 20 | 40 | 20 | 0 | 0 | 20 | 0 | 0 | 0 | 0 | 0 | 0 | 0 | 0 | 0 | 0 | 0 | 0 | 0 | GH |
| *Tetracerus quadricornis* | 70 | 15 | 15 | 0 | 0 | 0 | 0 | 0 | 0 | 0 | 0 | 0 | 0 | 0 | 0 | 0 | 0 | 0 | 0 | Gr |
| *Tragelaphus angasii* | 40 | 20 | 20 | 20 | 0 | 0 | 0 | 0 | 0 | 0 | 0 | 0 | 0 | 0 | 0 | 0 | 0 | 0 | 0 | MF |
| *Tragelaphus buxtoni* | 30 | 35 | 0 | 0 | 0 | 35 | 0 | 0 | 0 | 0 | 0 | 0 | 0 | 0 | 0 | 0 | 0 | 0 | 0 | MF |
| *Tragelaphus eurycerus* | 25 | 30 | 0 | 0 | 0 | 25 | 0 | 0 | 20 | 0 | 0 | 0 | 0 | 0 | 0 | 0 | 0 | 0 | 0 | MF |
| *Tragelaphus imberbis* | 20 | 40 | 20 | 0 | 0 | 20 | 0 | 0 | 0 | 0 | 0 | 0 | 0 | 0 | 0 | 0 | 0 | 0 | 0 | GH |
| *Tragelaphus scriptus* | 5 | 35 | 0 | 20 | 0 | 40 | 0 | 0 | 0 | 0 | 0 | 0 | 0 | 0 | 0 | 0 | 0 | 0 | 0 | GH |
| *Tragelaphus spekii* | 50 | 50 | 0 | 0 | 0 | 0 | 0 | 0 | 0 | 0 | 0 | 0 | 0 | 0 | 0 | 0 | 0 | 0 | 0 | MF |
| *Tragelaphus strepsiceros* | 10 | 25 | 20 | 20 | 0 | 25 | 0 | 0 | 0 | 0 | 0 | 0 | 0 | 0 | 0 | 0 | 0 | 0 | 0 | GH |
| *Lama glama* | 35 | 35 | 0 | 0 | 30 | 0 | 0 | 0 | 0 | 0 | 0 | 0 | 0 | 0 | 0 | 0 | 0 | 0 | 0 | MF |
| *Vicugna vicugna* | 100 | 0 | 0 | 0 | 0 | 0 | 0 | 0 | 0 | 0 | 0 | 0 | 0 | 0 | 0 | 0 | 0 | 0 | 0 | Gr |
| *Alces alces* | 70 | 0 | 0 | 0 | 0 | 0 | 0 | 0 | 0 | 30 | 0 | 0 | 0 | 0 | 0 | 0 | 0 | 0 | 0 | Gr |
| *Alces americanus* | 0 | 40 | 0 | 0 | 0 | 0 | 0 | 15 | 0 | 30 | 15 | 0 | 0 | 0 | 0 | 0 | 0 | 0 | 0 | GH |
| *Axis axis* | 20 | 2 | 20 | 19 | 0 | 0 | 0 | 0 | 0 | 19 | 0 | 20 | 0 | 0 | 0 | 0 | 0 | 0 | 0 | GH |
| *Axis calamianensis* | 0 | 100 | 0 | 0 | 0 | 0 | 0 | 0 | 0 | 0 | 0 | 0 | 0 | 0 | 0 | 0 | 0 | 0 | 0 | Fl |
| *Axis kuhlii* | 50 | 50 | 0 | 0 | 0 | 0 | 0 | 0 | 0 | 0 | 0 | 0 | 0 | 0 | 0 | 0 | 0 | 0 | 0 | MF |
| *Axis porcinus* | 55 | 35 | 10 | 0 | 0 | 0 | 0 | 0 | 0 | 0 | 0 | 0 | 0 | 0 | 0 | 0 | 0 | 0 | 0 | MF |
| *Blastocerus dichotomus* | 40 | 0 | 0 | 0 | 0 | 30 | 0 | 0 | 0 | 30 | 0 | 0 | 0 | 0 | 0 | 0 | 0 | 0 | 0 | MF |
| *Capreolus capreolus* | 20 | 25 | 0 | 0 | 0 | 55 | 0 | 0 | 0 | 0 | 0 | 0 | 0 | 0 | 0 | 0 | 0 | 0 | 0 | MF |
| *Capreolus pygargus* | 17 | 23 | 0 | 0 | 0 | 60 | 0 | 0 | 0 | 0 | 0 | 0 | 0 | 0 | 0 | 0 | 0 | 0 | 0 | GH |
| *Cervus elaphus* | 50 | 15 | 0 | 0 | 0 | 15 | 0 | 0 | 0 | 15 | 0 | 5 | 0 | 0 | 0 | 0 | 0 | 0 | 0 | MF |
| *Cervus nippon* | 20 | 30 | 0 | 0 | 0 | 20 | 0 | 0 | 10 | 10 | 0 | 10 | 0 | 0 | 0 | 0 | 0 | 0 | 0 | MF |
| *Dama dama* | 30 | 40 | 0 | 0 | 0 | 20 | 0 | 0 | 0 | 0 | 10 | 0 | 0 | 0 | 0 | 0 | 0 | 0 | 0 | MF |
| *Elaphodus cephalophus* | 35 | 35 | 30 | 0 | 0 | 0 | 0 | 0 | 0 | 0 | 0 | 0 | 0 | 0 | 0 | 0 | 0 | 0 | 0 | MF |
| *Elaphurus davidianus* | 85 | 0 | 0 | 0 | 0 | 0 | 0 | 0 | 0 | 15 | 0 | 0 | 0 | 0 | 0 | 0 | 0 | 0 | 0 | Gr |
| *Hippocamelus antisensis* | 60 | 0 | 0 | 0 | 0 | 0 | 0 | 0 | 0 | 40 | 0 | 0 | 0 | 0 | 0 | 0 | 0 | 0 | 0 | Gr |
| *Hippocamelus bisulcus* | 5 | 95 | 0 | 0 | 0 | 0 | 0 | 0 | 0 | 0 | 0 | 0 | 0 | 0 | 0 | 0 | 0 | 0 | 0 | Fl |

| | | | | | | | | | | | | | | | | | | | | |
|---|---|---|---|---|---|---|---|---|---|---|---|---|---|---|---|---|---|---|---|---|
| *Hydropotes inermis* | 60 | 0 | 0 | 0 | 0 | 0 | 0 | 0 | 0 | 40 | 0 | 0 | 0 | 0 | 0 | 0 | 0 | 0 | 0 | Gr |
| *Mazama americana* | 0 | 20 | 50 | 3 | 0 | 0 | 0 | 6 | 6 | 0 | 5 | 10 | 0 | 0 | 0 | 0 | 0 | 0 | 0 | Fr |
| *Mazama bororo* | 0 | 50 | 50 | 0 | 0 | 0 | 0 | 0 | 0 | 0 | 0 | 0 | 0 | 0 | 0 | 0 | 0 | 0 | 0 | Fr |
| *Mazama bricenii* | 0 | 50 | 50 | 0 | 0 | 0 | 0 | 0 | 0 | 0 | 0 | 0 | 0 | 0 | 0 | 0 | 0 | 0 | 0 | Fr |
| *Mazama chunyi* | 0 | 50 | 50 | 0 | 0 | 0 | 0 | 0 | 0 | 0 | 0 | 0 | 0 | 0 | 0 | 0 | 0 | 0 | 0 | Fr |
| *Mazama gouazoubira* | 5 | 20 | 50 | 10 | 0 | 0 | 0 | 10 | 0 | 0 | 5 | 0 | 0 | 0 | 0 | 0 | 0 | 0 | 0 | Fr |
| *Mazama nana* | 0 | 50 | 50 | 0 | 0 | 0 | 0 | 0 | 0 | 0 | 0 | 0 | 0 | 0 | 0 | 0 | 0 | 0 | 0 | Fr |
| *Mazama pandora* | 0 | 25 | 25 | 15 | 0 | 20 | 0 | 0 | 0 | 0 | 0 | 15 | 0 | 0 | 0 | 0 | 0 | 0 | 0 | SF |
| *Mazama rufina* | 0 | 50 | 50 | 0 | 0 | 0 | 0 | 0 | 0 | 0 | 0 | 0 | 0 | 0 | 0 | 0 | 0 | 0 | 0 | Fr |
| *Mazama temama* | 0 | 50 | 50 | 0 | 0 | 0 | 0 | 0 | 0 | 0 | 0 | 0 | 0 | 0 | 0 | 0 | 0 | 0 | 0 | Fr |
| *Muntiacus atherodes* | 20 | 20 | 20 | 0 | 0 | 20 | 0 | 0 | 20 | 0 | 0 | 0 | 0 | 0 | 0 | 0 | 0 | 0 | 0 | GH |
| *Muntiacus crinifrons* | 20 | 40 | 20 | 0 | 0 | 20 | 0 | 0 | 0 | 0 | 0 | 0 | 0 | 0 | 0 | 0 | 0 | 0 | 0 | GH |
| *Muntiacus feae* | 20 | 40 | 40 | 0 | 0 | 0 | 0 | 0 | 0 | 0 | 0 | 0 | 0 | 0 | 0 | 0 | 0 | 0 | 0 | SF |
| *Muntiacus gongshanensis* | 15 | 20 | 20 | 0 | 0 | 15 | 0 | 0 | 10 | 0 | 10 | 0 | 0 | 0 | 0 | 10 | 0 | 0 | 0 | GH |
| *Muntiacus muntjak* | 15 | 20 | 20 | 0 | 0 | 15 | 0 | 0 | 10 | 0 | 10 | 0 | 0 | 0 | 0 | 10 | 0 | 0 | 0 | GH |
| *Muntiacus puhoatensis* | 15 | 25 | 20 | 0 | 0 | 15 | 0 | 0 | 10 | 0 | 10 | 0 | 0 | 0 | 0 | 5 | 0 | 0 | 0 | GH |
| *Muntiacus putaoensis* | 0 | 25 | 40 | 10 | 0 | 10 | 0 | 0 | 5 | 0 | 0 | 0 | 0 | 0 | 0 | 10 | 0 | 0 | 0 | SF |
| *Muntiacus reevesi* | 0 | 20 | 20 | 0 | 0 | 15 | 0 | 0 | 10 | 0 | 10 | 0 | 0 | 0 | 10 | 15 | 0 | 0 | 0 | Om |
| *Muntiacus rooseveltorum* | 20 | 40 | 40 | 0 | 0 | 0 | 0 | 0 | 0 | 0 | 0 | 0 | 0 | 0 | 0 | 0 | 0 | 0 | 0 | SF |
| *Muntiacus truongsonensis* | 15 | 25 | 20 | 0 | 0 | 15 | 0 | 0 | 10 | 0 | 10 | 0 | 0 | 0 | 0 | 5 | 0 | 0 | 0 | GH |
| *Muntiacus vuquangensis* | 15 | 20 | 20 | 0 | 0 | 15 | 0 | 0 | 10 | 0 | 10 | 0 | 0 | 0 | 0 | 10 | 0 | 0 | 0 | GH |
| *Odocoileus hemionus* | 10 | 40 | 10 | 0 | 0 | 30 | 0 | 0 | 10 | 0 | 0 | 0 | 0 | 0 | 0 | 0 | 0 | 0 | 0 | GH |
| *Odocoileus virginianus* | 8 | 23 | 8 | 0 | 0 | 23 | 0 | 10 | 14 | 0 | 0 | 8 | 0 | 0 | 0 | 6 | 0 | 0 | 0 | GH |
| *Ozotoceros bezoarticus* | 10 | 20 | 10 | 0 | 0 | 40 | 0 | 0 | 10 | 10 | 0 | 0 | 0 | 0 | 0 | 0 | 0 | 0 | 0 | GH |
| *Przewalskium albirostris* | 80 | 10 | 0 | 0 | 0 | 10 | 0 | 0 | 0 | 0 | 0 | 0 | 0 | 0 | 0 | 0 | 0 | 0 | 0 | Gr |
| *Pudu mephistophiles* | 0 | 50 | 15 | 0 | 0 | 10 | 0 | 0 | 15 | 0 | 10 | 0 | 0 | 0 | 0 | 0 | 0 | 0 | 0 | Fl |
| *Pudu puda* | 0 | 50 | 15 | 0 | 0 | 10 | 0 | 0 | 15 | 0 | 10 | 0 | 0 | 0 | 0 | 0 | 0 | 0 | 0 | Fl |
| *Rangifer tarandus* | 25 | 15 | 0 | 0 | 25 | 5 | 0 | 10 | 0 | 5 | 10 | 5 | 0 | 0 | 0 | 0 | 0 | 0 | 0 | MF |
| *Rucervus duvaucelii* | 95 | 5 | 0 | 0 | 0 | 0 | 0 | 0 | 0 | 0 | 0 | 0 | 0 | 0 | 0 | 0 | 0 | 0 | 0 | Gr |
| *Rucervus eldii* | 40 | 30 | 30 | 0 | 0 | 0 | 0 | 0 | 0 | 0 | 0 | 0 | 0 | 0 | 0 | 0 | 0 | 0 | 0 | MF |
| *Rusa alfredi* | 25 | 25 | 25 | 0 | 0 | 25 | 0 | 0 | 0 | 0 | 0 | 0 | 0 | 0 | 0 | 0 | 0 | 0 | 0 | GH |

| | | | | | | | | | | | | | | | | | | | | |
|---|---|---|---|---|---|---|---|---|---|---|---|---|---|---|---|---|---|---|---|---|
| *Rusa marianna* | 25 | 25 | 25 | 0 | 0 | 25 | 0 | 0 | 0 | 0 | 0 | 0 | 0 | 0 | 0 | 0 | 0 | 0 | 0 | GH |
| *Rusa timorensis* | 40 | 40 | 0 | 0 | 0 | 0 | 0 | 0 | 0 | 0 | 20 | 0 | 0 | 0 | 0 | 0 | 0 | 0 | 0 | MF |
| *Rusa unicolor* | 20 | 20 | 20 | 0 | 0 | 20 | 0 | 0 | 0 | 0 | 20 | 0 | 0 | 0 | 0 | 0 | 0 | 0 | 0 | GH |
| *Giraffa camelopardalis* | 0 | 25 | 25 | 25 | 0 | 0 | 0 | 0 | 25 | 0 | 0 | 0 | 0 | 0 | 0 | 0 | 0 | 0 | 0 | SF |
| *Okapia johnstoni* | 10 | 70 | 10 | 0 | 0 | 0 | 0 | 0 | 0 | 0 | 0 | 10 | 0 | 0 | 0 | 0 | 0 | 0 | 0 | Fl |
| *Hexaprotodon liberiensis* | 20 | 0 | 15 | 0 | 0 | 15 | 0 | 0 | 0 | 35 | 0 | 15 | 0 | 0 | 0 | 0 | 0 | 0 | 0 | GH |
| *Hippopotamus amphibius* | 100 | 0 | 0 | 0 | 0 | 0 | 0 | 0 | 0 | 0 | 0 | 0 | 0 | 0 | 0 | 0 | 0 | 0 | 0 | Gr |
| *Moschus anhuiensis* | 0 | 15 | 0 | 0 | 50 | 15 | 0 | 0 | 10 | 0 | 10 | 0 | 0 | 0 | 0 | 0 | 0 | 0 | 0 | GH |
| *Moschus berezovskii* | 0 | 15 | 0 | 0 | 50 | 15 | 0 | 0 | 10 | 0 | 10 | 0 | 0 | 0 | 0 | 0 | 0 | 0 | 0 | GH |
| *Moschus chrysogaster* | 30 | 30 | 0 | 0 | 20 | 0 | 20 | 0 | 0 | 0 | 0 | 0 | 0 | 0 | 0 | 0 | 0 | 0 | 0 | MF |
| *Moschus cupreus* | 30 | 30 | 0 | 0 | 20 | 0 | 20 | 0 | 0 | 0 | 0 | 0 | 0 | 0 | 0 | 0 | 0 | 0 | 0 | MF |
| *Moschus fuscus* | 30 | 10 | 0 | 0 | 30 | 30 | 0 | 0 | 0 | 0 | 0 | 0 | 0 | 0 | 0 | 0 | 0 | 0 | 0 | MF |
| *Moschus leucogaster* | 20 | 20 | 0 | 0 | 20 | 20 | 20 | 0 | 0 | 0 | 0 | 0 | 0 | 0 | 0 | 0 | 0 | 0 | 0 | GH |
| *Moschus moschiferus* | 0 | 15 | 0 | 0 | 50 | 15 | 0 | 0 | 10 | 0 | 10 | 0 | 0 | 0 | 0 | 0 | 0 | 0 | 0 | GH |
| *Babyrousa babyrussa* | 0 | 25 | 25 | 0 | 0 | 0 | 0 | 10 | 0 | 0 | 0 | 20 | 10 | 0 | 0 | 10 | 0 | 0 | 0 | Om |
| *Babyrousa celebensis* | 0 | 0 | 30 | 0 | 0 | 0 | 0 | 30 | 10 | 0 | 0 | 10 | 10 | 0 | 0 | 10 | 0 | 0 | 0 | Om |
| *Babyrousa togeanensis* | 0 | 20 | 40 | 0 | 0 | 0 | 0 | 40 | 0 | 0 | 0 | 0 | 0 | 0 | 0 | 0 | 0 | 0 | 0 | Om |
| *Hylochoerus meinertzhageni* | 40 | 15 | 10 | 0 | 0 | 10 | 0 | 0 | 0 | 10 | 0 | 0 | 0 | 0 | 5 | 10 | 0 | 0 | 0 | MF |
| *Phacochoerus aethiopicus* | 45 | 0 | 5 | 0 | 0 | 0 | 0 | 30 | 0 | 0 | 0 | 0 | 5 | 0 | 2 | 13 | 0 | 0 | 0 | MF |
| *Phacochoerus africanus* | 50 | 0 | 15 | 0 | 0 | 0 | 0 | 15 | 0 | 0 | 15 | 0 | 0 | 0 | 5 | 0 | 0 | 0 | 0 | Gr |
| *Potamochoerus larvatus* | 0 | 0 | 15 | 0 | 0 | 0 | 0 | 30 | 0 | 15 | 0 | 0 | 15 | 0 | 10 | 15 | 0 | 0 | 0 | Om |
| *Potamochoerus porcus* | 9 | 0 | 9 | 0 | 0 | 0 | 0 | 19 | 18 | 9 | 0 | 9 | 9 | 0 | 9 | 9 | 0 | 0 | 0 | Om |
| *Sus ahoenobarbus* | 0 | 10 | 40 | 0 | 0 | 10 | 0 | 10 | 10 | 0 | 0 | 0 | 10 | 0 | 10 | 0 | 0 | 0 | 0 | Om |
| *Sus barbatus* | 0 | 10 | 40 | 0 | 0 | 10 | 0 | 10 | 10 | 0 | 0 | 0 | 10 | 0 | 10 | 0 | 0 | 0 | 0 | Om |
| *Sus bucculentus* | 4 | 16 | 8 | 0 | 0 | 8 | 0 | 12 | 16 | 8 | 8 | 4 | 8 | 0 | 4 | 4 | 0 | 0 | 0 | GH |
| *Sus cebifrons* | 0 | 0 | 40 | 40 | 0 | 0 | 0 | 0 | 0 | 0 | 0 | 0 | 20 | 0 | 0 | 0 | 0 | 0 | 0 | Fr |
| *Sus celebensis* | 0 | 20 | 20 | 0 | 0 | 0 | 0 | 20 | 0 | 0 | 0 | 0 | 20 | 10 | 10 | 0 | 0 | 0 | 0 | Om |
| *Sus oliveri* | 0 | 0 | 20 | 0 | 0 | 0 | 0 | 20 | 0 | 0 | 0 | 0 | 20 | 0 | 20 | 20 | 0 | 0 | 0 | Om |
| *Sus philippensis* | 0 | 0 | 20 | 0 | 0 | 0 | 0 | 20 | 0 | 0 | 0 | 0 | 20 | 0 | 20 | 20 | 0 | 0 | 0 | Om |
| *Sus salvanius* | 0 | 20 | 20 | 0 | 0 | 0 | 0 | 40 | 0 | 0 | 0 | 0 | 10 | 0 | 0 | 10 | 0 | 0 | 0 | Om |
| *Sus scrofa* | 4 | 8 | 8 | 4 | 0 | 8 | 0 | 12 | 16 | 12 | 8 | 4 | 8 | 0 | 4 | 4 | 0 | 0 | 0 | GH |

| | | | | | | | | | | | | | | | | | | | | |
|---|---|---|---|---|---|---|---|---|---|---|---|---|---|---|---|---|---|---|---|---|
| *Sus verrucosus* | 0 | 10 | 15 | 10 | 0 | 15 | 0 | 25 | 0 | 0 | 0 | 0 | 0 | 0 | 10 | 15 | 0 | 0 | 0 | Om |
| *Catagonus wagneri* | 0 | 40 | 0 | 20 | 0 | 0 | 0 | 20 | 20 | 0 | 0 | 0 | 0 | 0 | 0 | 0 | 0 | 0 | 0 | SF |
| *Pecari tajacu* | 13 | 12 | 13 | 0 | 0 | 13 | 0 | 28 | 0 | 0 | 0 | 13 | 0 | 0 | 4 | 4 | 0 | 0 | 0 | GH |
| *Tayassu pecari* | 0 | 15 | 15 | 0 | 0 | 0 | 0 | 15 | 15 | 0 | 0 | 15 | 15 | 0 | 5 | 5 | 0 | 0 | 0 | Om |
| *Hyemoschus aquaticus* | 0 | 30 | 55 | 0 | 0 | 0 | 0 | 0 | 0 | 0 | 0 | 0 | 10 | 0 | 0 | 5 | 0 | 0 | 0 | Fr |
| *Moschiola meminna* | 0 | 30 | 40 | 0 | 0 | 30 | 0 | 0 | 0 | 0 | 0 | 0 | 0 | 0 | 0 | 0 | 0 | 0 | 0 | SF |
| *Tragulus javanicus* | 0 | 30 | 35 | 0 | 0 | 30 | 0 | 0 | 0 | 0 | 0 | 0 | 5 | 0 | 0 | 0 | 0 | 0 | 0 | SF |
| *Tragulus kanchil* | 15 | 15 | 35 | 35 | 0 | 0 | 0 | 0 | 0 | 0 | 0 | 0 | 0 | 0 | 0 | 0 | 0 | 0 | 0 | SF |
| *Tragulus napu* | 20 | 25 | 25 | 0 | 0 | 0 | 0 | 0 | 0 | 25 | 0 | 0 | 0 | 0 | 0 | 5 | 0 | 0 | 0 | GH |
| *Tragulus nigricans* | 20 | 25 | 25 | 0 | 0 | 0 | 0 | 0 | 0 | 25 | 0 | 0 | 0 | 0 | 0 | 5 | 0 | 0 | 0 | GH |
| *Tragulus versicolor* | 20 | 25 | 25 | 0 | 0 | 0 | 0 | 0 | 0 | 25 | 0 | 0 | 0 | 0 | 0 | 5 | 0 | 0 | 0 | GH |
| *Tragulus williamsoni* | 20 | 25 | 25 | 0 | 0 | 0 | 0 | 0 | 0 | 25 | 0 | 0 | 0 | 0 | 0 | 5 | 0 | 0 | 0 | GH |
| *Ailurus fulgens* | 5 | 65 | 5 | 5 | 5 | 0 | 0 | 5 | 0 | 0 | 0 | 5 | 0 | 0 | 0 | 5 | 0 | 0 | 0 | Fl |
| *Atelocynus microtis* | 0 | 0 | 10 | 0 | 0 | 0 | 0 | 0 | 0 | 0 | 0 | 0 | 30 | 30 | 0 | 30 | 0 | 0 | 0 | Ps |
| *Canis adustus* | 0 | 0 | 30 | 0 | 0 | 0 | 0 | 0 | 0 | 0 | 0 | 0 | 30 | 0 | 20 | 20 | 0 | 0 | 0 | Om |
| *Canis aureus* | 0 | 0 | 45 | 0 | 0 | 0 | 0 | 0 | 0 | 0 | 0 | 0 | 20 | 5 | 5 | 15 | 10 | 0 | 0 | Om |
| *Canis latrans* | 0 | 0 | 0 | 0 | 0 | 0 | 0 | 0 | 0 | 0 | 0 | 0 | 5 | 0 | 5 | 90 | 0 | 0 | 0 | SC |
| *Canis lupus* | 0 | 0 | 0 | 0 | 0 | 0 | 0 | 0 | 0 | 0 | 0 | 0 | 0 | 0 | 10 | 0 | 0 | 90 | 0 | LC |
| *Canis mesomelas* | 0 | 0 | 30 | 0 | 0 | 0 | 0 | 0 | 0 | 0 | 0 | 0 | 50 | 0 | 5 | 10 | 5 | 0 | 0 | Om |
| *Canis simensis* | 0 | 0 | 0 | 0 | 0 | 0 | 0 | 0 | 0 | 0 | 0 | 0 | 0 | 0 | 0 | 100 | 0 | 0 | 0 | SC |
| *Cerdocyon thous* | 0 | 0 | 0 | 0 | 0 | 0 | 0 | 0 | 0 | 0 | 0 | 0 | 50 | 0 | 0 | 50 | 0 | 0 | 0 | SC |
| *Chrysocyon brachyurus* | 0 | 0 | 30 | 0 | 0 | 0 | 0 | 0 | 0 | 0 | 0 | 0 | 0 | 0 | 0 | 30 | 40 | 0 | 0 | Om |
| *Cuon alpinus* | 0 | 0 | 5 | 0 | 0 | 0 | 0 | 0 | 0 | 0 | 0 | 0 | 0 | 0 | 0 | 5 | 0 | 45 | 45 | LC |
| *Lycalopex culpaeus* | 0 | 0 | 10 | 0 | 0 | 0 | 0 | 0 | 0 | 0 | 0 | 0 | 20 | 0 | 10 | 30 | 30 | 0 | 0 | Om |
| *Lycalopex fulvipes* | 0 | 0 | 5 | 0 | 0 | 0 | 0 | 0 | 10 | 0 | 0 | 0 | 30 | 0 | 5 | 50 | 0 | 0 | 0 | SC |
| *Lycalopex griseus* | 0 | 0 | 15 | 0 | 0 | 0 | 0 | 0 | 10 | 0 | 0 | 0 | 10 | 0 | 25 | 30 | 10 | 0 | 0 | Om |
| *Lycalopex gymnocercus* | 0 | 5 | 10 | 0 | 0 | 0 | 0 | 0 | 0 | 0 | 0 | 0 | 0 | 0 | 0 | 60 | 25 | 0 | 0 | SC |
| *Lycalopex sechurae* | 0 | 0 | 20 | 0 | 0 | 0 | 0 | 0 | 20 | 0 | 0 | 0 | 20 | 0 | 20 | 20 | 0 | 0 | 0 | Om |
| *Lycalopex vetulus* | 0 | 0 | 5 | 0 | 0 | 0 | 0 | 0 | 0 | 0 | 0 | 0 | 90 | 0 | 0 | 5 | 0 | 0 | 0 | IF |
| *Lycaon pictus* | 0 | 0 | 0 | 0 | 0 | 0 | 0 | 0 | 0 | 0 | 0 | 0 | 0 | 0 | 0 | 0 | 0 | 50 | 50 | LC |
| *Nyctereutes procyonoides* | 0 | 0 | 0 | 0 | 0 | 0 | 0 | 0 | 0 | 0 | 0 | 0 | 40 | 20 | 20 | 20 | 0 | 0 | 0 | Ps |

| | | | | | | | | | | | | | | | | | | | | |
|---|---|---|---|---|---|---|---|---|---|---|---|---|---|---|---|---|---|---|---|---|
| *Otocyon megalotis* | 0 | 0 | 5 | 0 | 0 | 0 | 0 | 0 | 0 | 0 | 0 | 0 | 90 | 0 | 0 | 5 | 0 | 0 | 0 | IF |
| *Speothos venaticus* | 0 | 0 | 0 | 0 | 0 | 0 | 0 | 0 | 0 | 0 | 0 | 0 | 0 | 0 | 0 | 0 | 65 | 35 | 0 | SC |
| *Urocyon cinereoargenteus* | 0 | 0 | 20 | 0 | 0 | 15 | 0 | 0 | 0 | 0 | 0 | 0 | 10 | 0 | 10 | 40 | 5 | 0 | 0 | Om |
| *Urocyon littoralis* | 0 | 0 | 45 | 0 | 0 | 0 | 0 | 0 | 0 | 0 | 0 | 0 | 45 | 0 | 2 | 8 | 0 | 0 | 0 | Fr |
| *Vulpes bengalensis* | 0 | 0 | 0 | 25 | 0 | 0 | 0 | 0 | 0 | 0 | 0 | 0 | 50 | 0 | 0 | 25 | 0 | 0 | 0 | IF |
| *Vulpes cana* | 0 | 0 | 50 | 0 | 0 | 0 | 0 | 0 | 0 | 0 | 0 | 0 | 45 | 0 | 0 | 5 | 0 | 0 | 0 | Fr |
| *Vulpes chama* | 0 | 0 | 15 | 0 | 0 | 5 | 0 | 0 | 0 | 0 | 0 | 0 | 35 | 0 | 5 | 35 | 5 | 0 | 0 | Om |
| *Vulpes corsac* | 0 | 0 | 5 | 0 | 0 | 5 | 0 | 0 | 0 | 0 | 0 | 0 | 25 | 0 | 25 | 40 | 0 | 0 | 0 | Om |
| *Vulpes ferrilata* | 0 | 0 | 0 | 0 | 0 | 0 | 0 | 0 | 0 | 0 | 0 | 0 | 0 | 0 | 10 | 55 | 35 | 0 | 0 | SC |
| *Vulpes lagopus* | 0 | 0 | 15 | 0 | 0 | 0 | 0 | 0 | 0 | 0 | 0 | 0 | 15 | 5 | 20 | 45 | 0 | 0 | 0 | SC |
| *Vulpes macrotis* | 0 | 0 | 15 | 0 | 0 | 0 | 0 | 0 | 0 | 0 | 0 | 0 | 0 | 0 | 15 | 35 | 35 | 0 | 0 | Om |
| *Vulpes pallida* | 0 | 0 | 20 | 0 | 0 | 15 | 0 | 0 | 0 | 0 | 0 | 0 | 25 | 0 | 0 | 40 | 0 | 0 | 0 | Om |
| *Vulpes rueppellii* | 0 | 0 | 0 | 0 | 0 | 0 | 0 | 20 | 0 | 0 | 0 | 0 | 55 | 0 | 0 | 25 | 0 | 0 | 0 | IF |
| *Vulpes velox* | 20 | 0 | 20 | 0 | 0 | 0 | 0 | 0 | 0 | 0 | 0 | 0 | 20 | 0 | 20 | 20 | 0 | 0 | 0 | Om |
| *Vulpes vulpes* | 0 | 0 | 20 | 0 | 0 | 0 | 0 | 0 | 0 | 0 | 0 | 0 | 15 | 0 | 15 | 30 | 20 | 0 | 0 | Om |
| *Vulpes zerda* | 0 | 20 | 20 | 0 | 0 | 0 | 0 | 20 | 0 | 0 | 0 | 0 | 20 | 0 | 0 | 20 | 0 | 0 | 0 | Om |
| *Cryptoprocta ferox* | 0 | 0 | 0 | 0 | 0 | 0 | 0 | 0 | 0 | 0 | 0 | 0 | 20 | 0 | 0 | 40 | 40 | 0 | 0 | SC |
| *Acinonyx jubatus* | 0 | 0 | 0 | 0 | 0 | 0 | 0 | 0 | 0 | 0 | 0 | 0 | 0 | 0 | 0 | 0 | 80 | 20 | 0 | SC |
| *Caracal caracal* | 0 | 0 | 0 | 0 | 0 | 0 | 0 | 0 | 0 | 0 | 0 | 0 | 0 | 0 | 0 | 60 | 40 | 0 | 0 | SC |
| *Catopuma badia* | 0 | 0 | 0 | 0 | 0 | 0 | 0 | 0 | 0 | 0 | 0 | 0 | 0 | 0 | 20 | 50 | 30 | 0 | 0 | SC |
| *Catopuma temminckii* | 0 | 0 | 0 | 0 | 0 | 0 | 0 | 0 | 0 | 0 | 0 | 0 | 0 | 0 | 0 | 50 | 50 | 0 | 0 | SC |
| *Felis bieti* | 0 | 0 | 0 | 0 | 0 | 0 | 0 | 0 | 0 | 0 | 0 | 0 | 0 | 0 | 0 | 100 | 0 | 0 | 0 | SC |
| *Felis chaus* | 0 | 0 | 10 | 0 | 0 | 0 | 0 | 0 | 0 | 0 | 0 | 0 | 13 | 2 | 0 | 55 | 15 | 5 | 0 | SC |
| *Felis manul* | 0 | 0 | 0 | 0 | 0 | 0 | 0 | 0 | 0 | 0 | 0 | 0 | 5 | 0 | 5 | 90 | 0 | 0 | 0 | SC |
| *Felis margarita* | 0 | 0 | 0 | 0 | 0 | 0 | 0 | 0 | 0 | 0 | 0 | 0 | 10 | 0 | 0 | 90 | 0 | 0 | 0 | SC |
| *Felis nigripes* | 0 | 0 | 0 | 0 | 0 | 0 | 0 | 0 | 0 | 0 | 0 | 0 | 0 | 0 | 0 | 100 | 0 | 0 | 0 | SC |
| *Felis silvestris* | 0 | 0 | 0 | 0 | 0 | 0 | 0 | 0 | 0 | 0 | 0 | 0 | 15 | 0 | 15 | 40 | 30 | 0 | 0 | SC |
| *Leopardus braccatus* | 0 | 0 | 0 | 0 | 0 | 0 | 0 | 0 | 0 | 0 | 0 | 0 | 0 | 0 | 0 | 70 | 30 | 0 | 0 | SC |
| *Leopardus colocolo* | 0 | 0 | 0 | 0 | 0 | 0 | 0 | 0 | 0 | 0 | 0 | 0 | 0 | 0 | 0 | 100 | 0 | 0 | 0 | SC |
| *Leopardus geoffroyi* | 0 | 0 | 0 | 0 | 0 | 0 | 0 | 0 | 0 | 0 | 0 | 0 | 0 | 2 | 0 | 98 | 0 | 0 | 0 | SC |
| *Leopardus guigna* | 0 | 0 | 0 | 0 | 0 | 0 | 0 | 0 | 0 | 0 | 0 | 0 | 15 | 0 | 0 | 85 | 0 | 0 | 0 | SC |

| | | | | | | | | | | | | | | | | | | | | |
|---|---|---|---|---|---|---|---|---|---|---|---|---|---|---|---|---|---|---|---|---|
| *Leopardus jacobitus* | 0 | 0 | 0 | 0 | 0 | 0 | 0 | 0 | 0 | 0 | 0 | 0 | 0 | 0 | 0 | 80 | 20 | 0 | 0 | SC |
| *Leopardus pajeros* | 0 | 0 | 0 | 0 | 0 | 0 | 0 | 0 | 0 | 0 | 0 | 0 | 0 | 0 | 0 | 80 | 20 | 0 | 0 | SC |
| *Leopardus pardalis* | 0 | 0 | 0 | 0 | 0 | 0 | 0 | 0 | 0 | 0 | 0 | 0 | 2 | 2 | 0 | 86 | 10 | 0 | 0 | SC |
| *Leopardus tigrinus* | 5 | 0 | 0 | 0 | 0 | 0 | 0 | 0 | 0 | 0 | 0 | 0 | 0 | 0 | 35 | 60 | 0 | 0 | 0 | SC |
| *Leopardus wiedii* | 0 | 0 | 0 | 0 | 0 | 0 | 0 | 0 | 0 | 0 | 0 | 0 | 0 | 0 | 0 | 95 | 5 | 0 | 0 | SC |
| *Leptailurus serval* | 0 | 0 | 0 | 0 | 0 | 0 | 0 | 0 | 0 | 0 | 0 | 0 | 2 | 0 | 2 | 96 | 0 | 0 | 0 | SC |
| *Lynx canadensis* | 0 | 0 | 0 | 0 | 0 | 0 | 0 | 0 | 0 | 0 | 0 | 0 | 0 | 0 | 2 | 50 | 45 | 3 | 0 | SC |
| *Lynx lynx* | 0 | 0 | 0 | 0 | 0 | 0 | 0 | 0 | 0 | 0 | 0 | 0 | 0 | 0 | 0 | 0 | 0 | 100 | 0 | LC |
| *Lynx pardinus* | 0 | 0 | 0 | 0 | 0 | 0 | 0 | 0 | 0 | 0 | 0 | 0 | 0 | 0 | 0 | 50 | 50 | 0 | 0 | SC |
| *Lynx rufus* | 0 | 0 | 0 | 0 | 0 | 0 | 0 | 0 | 0 | 0 | 0 | 0 | 0 | 0 | 0 | 80 | 20 | 0 | 0 | SC |
| *Neofelis nebulosa* | 0 | 0 | 0 | 0 | 0 | 0 | 0 | 0 | 0 | 0 | 0 | 0 | 0 | 0 | 0 | 0 | 0 | 85 | 15 | LC |
| *Panthera leo* | 0 | 0 | 0 | 0 | 0 | 0 | 0 | 0 | 0 | 0 | 0 | 0 | 0 | 0 | 10 | 0 | 0 | 20 | 70 | LC |
| *Panthera onca* | 0 | 0 | 0 | 0 | 0 | 0 | 0 | 0 | 0 | 0 | 0 | 0 | 0 | 0 | 0 | 0 | 0 | 50 | 50 | LC |
| *Panthera pardus* | 0 | 0 | 0 | 0 | 0 | 0 | 0 | 0 | 0 | 0 | 0 | 0 | 0 | 0 | 0 | 0 | 0 | 100 | 0 | LC |
| *Panthera tigris* | 0 | 0 | 0 | 0 | 0 | 0 | 0 | 0 | 0 | 0 | 0 | 0 | 0 | 0 | 10 | 0 | 5 | 15 | 70 | LC |
| *Pardofelis marmorata* | 0 | 0 | 0 | 0 | 0 | 0 | 0 | 0 | 0 | 0 | 0 | 0 | 0 | 0 | 0 | 100 | 0 | 0 | 0 | SC |
| *Prionailurus bengalensis* | 0 | 0 | 0 | 0 | 0 | 0 | 0 | 0 | 0 | 0 | 0 | 0 | 5 | 0 | 0 | 95 | 0 | 0 | 0 | SC |
| *Prionailurus iriomotensis* | 0 | 0 | 0 | 0 | 0 | 0 | 0 | 0 | 0 | 0 | 0 | 0 | 2 | 2 | 0 | 96 | 0 | 0 | 0 | SC |
| *Prionailurus planiceps* | 0 | 0 | 0 | 0 | 0 | 0 | 0 | 0 | 0 | 0 | 0 | 0 | 10 | 80 | 0 | 10 | 0 | 0 | 0 | Ps |
| *Prionailurus rubiginosus* | 0 | 0 | 0 | 0 | 0 | 0 | 0 | 0 | 0 | 0 | 0 | 0 | 5 | 0 | 0 | 95 | 0 | 0 | 0 | SC |
| *Prionailurus viverrinus* | 5 | 0 | 0 | 0 | 0 | 0 | 0 | 0 | 0 | 0 | 0 | 0 | 15 | 70 | 0 | 10 | 0 | 0 | 0 | Ps |
| *Profelis aurata* | 0 | 0 | 0 | 0 | 0 | 0 | 0 | 0 | 0 | 0 | 0 | 0 | 0 | 2 | 0 | 83 | 15 | 0 | 0 | SC |
| *Puma concolor* | 0 | 0 | 0 | 0 | 0 | 0 | 0 | 0 | 0 | 0 | 0 | 0 | 0 | 0 | 0 | 0 | 10 | 70 | 20 | LC |
| *Puma yagouaroundi* | 0 | 5 | 0 | 0 | 0 | 0 | 0 | 0 | 0 | 0 | 0 | 0 | 5 | 5 | 0 | 85 | 0 | 0 | 0 | SC |
| *Uncia uncia* | 0 | 0 | 0 | 0 | 0 | 0 | 0 | 0 | 0 | 0 | 0 | 0 | 0 | 0 | 10 | 10 | 15 | 65 | 0 | LC |
| *Crocuta crocuta* | 0 | 0 | 0 | 0 | 0 | 0 | 0 | 0 | 0 | 0 | 0 | 0 | 0 | 0 | 30 | 0 | 0 | 35 | 35 | LC |
| *Hyaena brunnea* | 0 | 0 | 10 | 0 | 0 | 0 | 0 | 0 | 0 | 0 | 0 | 0 | 10 | 0 | 70 | 10 | 0 | 0 | 0 | Om |
| *Hyaena hyaena* | 0 | 0 | 10 | 0 | 0 | 0 | 0 | 0 | 0 | 0 | 0 | 0 | 10 | 0 | 75 | 5 | 0 | 0 | 0 | Om |
| *Proteles cristata* | 0 | 0 | 0 | 0 | 0 | 0 | 0 | 0 | 0 | 0 | 0 | 0 | 100 | 0 | 0 | 0 | 0 | 0 | 0 | IF |
| *Aonyx capensis* | 0 | 0 | 0 | 0 | 0 | 0 | 0 | 0 | 0 | 0 | 0 | 0 | 70 | 15 | 0 | 15 | 0 | 0 | 0 | IF |
| *Aonyx cinerea* | 0 | 0 | 0 | 0 | 0 | 0 | 0 | 0 | 0 | 0 | 0 | 0 | 70 | 15 | 0 | 15 | 0 | 0 | 0 | IF |

| | | | | | | | | | | | | | | | | | | | | |
|---|---|---|---|---|---|---|---|---|---|---|---|---|---|---|---|---|---|---|---|---|
| *Arctonyx collaris* | 0 | 0 | 30 | 0 | 0 | 0 | 0 | 10 | 0 | 0 | 0 | 0 | 30 | 0 | 0 | 30 | 0 | 0 | 0 | Om |
| *Eira barbara* | 0 | 0 | 30 | 0 | 0 | 0 | 0 | 0 | 0 | 0 | 0 | 0 | 30 | 0 | 0 | 40 | 0 | 0 | 0 | Om |
| *Galictis vittata* | 0 | 0 | 0 | 0 | 0 | 0 | 0 | 0 | 0 | 0 | 0 | 0 | 0 | 0 | 0 | 40 | 60 | 0 | 0 | SC |
| *Gulo gulo* | 0 | 0 | 5 | 0 | 0 | 0 | 0 | 5 | 5 | 0 | 0 | 0 | 0 | 0 | 50 | 10 | 15 | 10 | 0 | Om |
| *Hydrictis maculicollis* | 0 | 0 | 0 | 0 | 0 | 0 | 0 | 0 | 0 | 0 | 0 | 0 | 20 | 70 | 0 | 10 | 0 | 0 | 0 | Ps |
| *Lontra canadensis* | 0 | 0 | 5 | 0 | 0 | 0 | 0 | 0 | 0 | 0 | 0 | 0 | 20 | 65 | 0 | 10 | 0 | 0 | 0 | Ps |
| *Lontra longicaudis* | 0 | 0 | 0 | 0 | 0 | 0 | 0 | 0 | 0 | 0 | 0 | 0 | 20 | 70 | 0 | 10 | 0 | 0 | 0 | Ps |
| *Lontra provocax* | 0 | 0 | 0 | 0 | 0 | 0 | 0 | 0 | 0 | 0 | 0 | 0 | 15 | 75 | 0 | 10 | 0 | 0 | 0 | Ps |
| *Lutra lutra* | 0 | 5 | 0 | 0 | 0 | 0 | 0 | 0 | 0 | 0 | 0 | 0 | 20 | 65 | 0 | 10 | 0 | 0 | 0 | Ps |
| *Lutra sumatrana* | 0 | 0 | 0 | 0 | 0 | 0 | 0 | 0 | 0 | 0 | 0 | 0 | 20 | 80 | 0 | 0 | 0 | 0 | 0 | Ps |
| *Lutrogale perspicillata* | 0 | 0 | 0 | 0 | 0 | 0 | 0 | 0 | 0 | 0 | 0 | 0 | 20 | 70 | 0 | 10 | 0 | 0 | 0 | Ps |
| *Martes pennanti* | 0 | 0 | 10 | 0 | 0 | 0 | 0 | 0 | 10 | 0 | 0 | 10 | 10 | 0 | 0 | 10 | 50 | 0 | 0 | Om |
| *Meles anakuma* | 0 | 0 | 40 | 0 | 0 | 0 | 0 | 0 | 0 | 0 | 0 | 0 | 40 | 0 | 20 | 0 | 0 | 0 | 0 | Fr |
| *Meles leucurus* | 0 | 5 | 10 | 0 | 0 | 5 | 0 | 0 | 10 | 0 | 0 | 0 | 60 | 0 | 0 | 10 | 0 | 0 | 0 | IF |
| *Meles meles* | 0 | 0 | 5 | 0 | 0 | 0 | 0 | 5 | 5 | 0 | 0 | 5 | 50 | 5 | 5 | 20 | 0 | 0 | 0 | IF |
| *Mellivora capensis* | 0 | 10 | 10 | 0 | 0 | 0 | 0 | 5 | 0 | 0 | 0 | 0 | 25 | 0 | 25 | 25 | 0 | 0 | 0 | Om |
| *Melogale everetti* | 0 | 0 | 10 | 0 | 0 | 0 | 0 | 0 | 0 | 0 | 0 | 0 | 70 | 0 | 10 | 10 | 0 | 0 | 0 | IF |
| *Pteronura brasiliensis* | 0 | 0 | 0 | 0 | 0 | 0 | 0 | 0 | 0 | 0 | 0 | 0 | 0 | 100 | 0 | 0 | 0 | 0 | 0 | Ps |
| *Taxidea taxus* | 0 | 0 | 0 | 0 | 0 | 0 | 0 | 0 | 5 | 0 | 0 | 0 | 5 | 5 | 5 | 40 | 40 | 0 | 0 | SC |
| *Nasua narica* | 0 | 0 | 25 | 0 | 0 | 0 | 0 | 0 | 0 | 0 | 0 | 0 | 25 | 0 | 25 | 25 | 0 | 0 | 0 | Om |
| *Nasua nasua* | 0 | 10 | 25 | 0 | 0 | 0 | 0 | 0 | 0 | 0 | 0 | 0 | 30 | 0 | 10 | 25 | 0 | 0 | 0 | Om |
| *Procyon cancrivorus* | 0 | 8 | 52 | 0 | 0 | 0 | 0 | 0 | 8 | 0 | 0 | 0 | 16 | 8 | 0 | 8 | 0 | 0 | 0 | Fr |
| *Procyon lotor* | 0 | 10 | 25 | 0 | 0 | 10 | 0 | 0 | 15 | 0 | 0 | 0 | 20 | 5 | 5 | 10 | 0 | 0 | 0 | Om |
| *Procyon pygmaeus* | 0 | 0 | 15 | 0 | 0 | 0 | 0 | 0 | 0 | 0 | 0 | 0 | 70 | 0 | 0 | 15 | 0 | 0 | 0 | IF |
| *Ailuropoda melanoleuca* | 0 | 80 | 5 | 0 | 0 | 0 | 0 | 0 | 0 | 0 | 0 | 0 | 5 | 5 | 0 | 5 | 0 | 0 | 0 | Fl |
| *Helarctos malayanus* | 0 | 5 | 20 | 0 | 0 | 0 | 0 | 0 | 0 | 0 | 0 | 0 | 60 | 0 | 5 | 10 | 0 | 0 | 0 | IF |
| *Melursus ursinus* | 0 | 10 | 40 | 10 | 0 | 0 | 0 | 0 | 0 | 0 | 0 | 0 | 40 | 0 | 0 | 0 | 0 | 0 | 0 | Om |
| *Tremarctos ornatus* | 0 | 40 | 35 | 5 | 0 | 0 | 5 | 0 | 0 | 0 | 5 | 0 | 5 | 0 | 0 | 5 | 0 | 0 | 0 | SF |
| *Ursus americanus* | 20 | 20 | 20 | 20 | 0 | 0 | 0 | 0 | 0 | 0 | 0 | 0 | 10 | 0 | 10 | 0 | 0 | 0 | 0 | SF |
| *Ursus arctos* | 6 | 3 | 10 | 0 | 0 | 3 | 3 | 18 | 13 | 3 | 0 | 2 | 12 | 10 | 12 | 0 | 0 | 3 | 2 | LC |
| *Ursus thibetanus* | 0 | 0 | 11 | 0 | 0 | 0 | 0 | 21 | 15 | 0 | 8 | 0 | 10 | 0 | 10 | 10 | 5 | 10 | 0 | Om |

| | | | | | | | | | | | | | | | | | | | |
|---|---|---|---|---|---|---|---|---|---|---|---|---|---|---|---|---|---|---|---|
| *Arctictis binturong* | 0 | 13 | 35 | 0 | 0 | 0 | 0 | 0 | 0 | 0 | 0 | 0 | 13 | 13 | 13 | 13 | 0 | 0 | 0 | Om |
| *Chrotogale owstoni* | 0 | 0 | 30 | 0 | 0 | 0 | 0 | 0 | 0 | 0 | 0 | 0 | 40 | 0 | 0 | 30 | 0 | 0 | 0 | Om |
| *Civettictis civetta* | 0 | 0 | 25 | 0 | 0 | 0 | 0 | 0 | 0 | 0 | 0 | 0 | 25 | 0 | 25 | 25 | 0 | 0 | 0 | Om |
| *Cynogale bennettii* | 0 | 0 | 0 | 0 | 0 | 0 | 0 | 0 | 0 | 0 | 0 | 0 | 50 | 50 | 0 | 0 | 0 | 0 | 0 | Ps |
| *Macrogalidia musschenbroekii* | 30 | 0 | 35 | 0 | 0 | 0 | 0 | 0 | 0 | 0 | 0 | 0 | 0 | 0 | 0 | 35 | 0 | 0 | 0 | Om |
| *Paguma larvata* | 0 | 0 | 50 | 0 | 0 | 0 | 0 | 0 | 0 | 0 | 0 | 0 | 25 | 0 | 0 | 25 | 0 | 0 | 0 | Fr |
| *Paradoxurus hermaphroditus* | 0 | 0 | 40 | 20 | 0 | 0 | 0 | 0 | 0 | 0 | 0 | 0 | 20 | 0 | 0 | 20 | 0 | 0 | 0 | Om |
| *Paradoxurus jerdoni* | 0 | 0 | 97 | 0 | 0 | 0 | 0 | 0 | 0 | 0 | 0 | 0 | 2 | 0 | 0 | 1 | 0 | 0 | 0 | Fr |
| *Viverra civettina* | 0 | 20 | 20 | 0 | 0 | 0 | 0 | 0 | 0 | 0 | 0 | 0 | 0 | 20 | 0 | 40 | 0 | 0 | 0 | Om |
| *Viverra megaspila* | 0 | 0 | 30 | 0 | 0 | 0 | 0 | 10 | 0 | 0 | 0 | 0 | 20 | 0 | 0 | 40 | 0 | 0 | 0 | Om |
| *Viverra tangalunga* | 0 | 0 | 30 | 0 | 0 | 0 | 0 | 10 | 0 | 0 | 0 | 0 | 30 | 0 | 0 | 30 | 0 | 0 | 0 | Om |
| *Viverra zibetha* | 0 | 0 | 25 | 0 | 0 | 0 | 0 | 25 | 0 | 0 | 0 | 0 | 5 | 5 | 0 | 40 | 0 | 0 | 0 | Om |
| *Cabassous centralis* | 0 | 0 | 0 | 0 | 0 | 0 | 0 | 0 | 0 | 0 | 0 | 0 | 95 | 0 | 0 | 5 | 0 | 0 | 0 | IF |
| *Cabassous tatouay* | 0 | 0 | 0 | 0 | 0 | 0 | 0 | 0 | 0 | 0 | 0 | 0 | 95 | 0 | 0 | 5 | 0 | 0 | 0 | IF |
| *Cabassous unicinctus* | 0 | 0 | 0 | 0 | 0 | 0 | 0 | 0 | 0 | 0 | 0 | 0 | 100 | 0 | 0 | 0 | 0 | 0 | 0 | IF |
| *Chaetophractus villosus* | 0 | 20 | 20 | 0 | 0 | 0 | 0 | 0 | 0 | 0 | 0 | 0 | 20 | 0 | 20 | 20 | 0 | 0 | 0 | Om |
| *Dasypus kappleri* | 0 | 0 | 0 | 0 | 0 | 0 | 0 | 0 | 0 | 0 | 0 | 0 | 100 | 0 | 0 | 0 | 0 | 0 | 0 | IF |
| *Dasypus novemcinctus* | 0 | 0 | 3 | 0 | 0 | 0 | 0 | 0 | 6 | 0 | 0 | 3 | 83 | 0 | 0 | 5 | 0 | 0 | 0 | IF |
| *Dasypus pilosus* | 0 | 0 | 0 | 0 | 0 | 0 | 0 | 0 | 0 | 0 | 0 | 0 | 100 | 0 | 0 | 0 | 0 | 0 | 0 | IF |
| *Euphractus sexcinctus* | 0 | 0 | 30 | 0 | 0 | 0 | 0 | 30 | 30 | 0 | 0 | 0 | 4 | 0 | 3 | 3 | 0 | 0 | 0 | Om |
| *Priodontes maximus* | 0 | 0 | 0 | 0 | 0 | 0 | 0 | 0 | 0 | 0 | 0 | 0 | 90 | 0 | 5 | 5 | 0 | 0 | 0 | IF |
| *Dasyurus maculatus* | 0 | 0 | 0 | 0 | 0 | 0 | 0 | 0 | 0 | 0 | 0 | 0 | 35 | 0 | 5 | 20 | 20 | 20 | 0 | Om |
| *Sarcophilus harrisii* | 0 | 5 | 10 | 0 | 0 | 0 | 0 | 0 | 0 | 0 | 0 | 0 | 10 | 0 | 30 | 15 | 15 | 15 | 0 | Om |
| *Dendrolagus bennettianus* | 0 | 85 | 15 | 0 | 0 | 0 | 0 | 0 | 0 | 0 | 0 | 0 | 0 | 0 | 0 | 0 | 0 | 0 | 0 | Fl |
| *Dendrolagus dorianus* | 0 | 40 | 30 | 30 | 0 | 0 | 0 | 0 | 0 | 0 | 0 | 0 | 0 | 0 | 0 | 0 | 0 | 0 | 0 | SF |
| *Dendrolagus goodfellowi* | 15 | 45 | 15 | 15 | 0 | 0 | 0 | 0 | 10 | 0 | 0 | 0 | 0 | 0 | 0 | 0 | 0 | 0 | 0 | SF |
| *Dendrolagus inustus* | 0 | 40 | 30 | 0 | 0 | 0 | 0 | 0 | 0 | 0 | 30 | 0 | 0 | 0 | 0 | 0 | 0 | 0 | 0 | SF |
| *Dendrolagus lumholtzi* | 0 | 80 | 0 | 10 | 0 | 10 | 0 | 0 | 0 | 0 | 0 | 0 | 0 | 0 | 0 | 0 | 0 | 0 | 0 | Fl |
| *Dendrolagus matschiei* | 0 | 80 | 3 | 0 | 0 | 0 | 0 | 6 | 6 | 0 | 5 | 0 | 0 | 0 | 0 | 0 | 0 | 0 | 0 | Fl |
| *Dendrolagus mbaiso* | 0 | 70 | 30 | 0 | 0 | 0 | 0 | 0 | 0 | 0 | 0 | 0 | 0 | 0 | 0 | 0 | 0 | 0 | 0 | Fl |
| *Dendrolagus pulcherrimus* | 15 | 45 | 15 | 15 | 0 | 0 | 0 | 0 | 10 | 0 | 0 | 0 | 0 | 0 | 0 | 0 | 0 | 0 | 0 | SF |

| | | | | | | | | | | | | | | | | | | | | |
|---|---|---|---|---|---|---|---|---|---|---|---|---|---|---|---|---|---|---|---|---|
| *Dendrolagus scottae* | 0 | 100 | 0 | 0 | 0 | 0 | 0 | 0 | 0 | 0 | 0 | 0 | 0 | 0 | 0 | 0 | 0 | 0 | 0 | Fl |
| *Dendrolagus spadix* | 5 | 55 | 25 | 15 | 0 | 0 | 0 | 0 | 0 | 0 | 0 | 0 | 0 | 0 | 0 | 0 | 0 | 0 | 0 | Fl |
| *Dendrolagus stellarum* | 15 | 45 | 15 | 15 | 0 | 0 | 0 | 0 | 10 | 0 | 0 | 0 | 0 | 0 | 0 | 0 | 0 | 0 | 0 | SF |
| *Dendrolagus ursinus* | 0 | 65 | 0 | 0 | 0 | 0 | 0 | 0 | 0 | 0 | 35 | 0 | 0 | 0 | 0 | 0 | 0 | 0 | 0 | Fl |
| *Dorcopsis atrata* | 25 | 25 | 25 | 0 | 0 | 0 | 0 | 25 | 0 | 0 | 0 | 0 | 0 | 0 | 0 | 0 | 0 | 0 | 0 | GH |
| *Dorcopsis hageni* | 0 | 0 | 0 | 0 | 0 | 0 | 0 | 0 | 0 | 0 | 0 | 0 | 100 | 0 | 0 | 0 | 0 | 0 | 0 | IF |
| *Dorcopsis luctuosa* | 25 | 25 | 25 | 0 | 0 | 0 | 0 | 25 | 0 | 0 | 0 | 0 | 0 | 0 | 0 | 0 | 0 | 0 | 0 | GH |
| *Dorcopsis muelleri* | 5 | 55 | 25 | 15 | 0 | 0 | 0 | 0 | 0 | 0 | 0 | 0 | 0 | 0 | 0 | 0 | 0 | 0 | 0 | Fl |
| *Macropus agilis* | 20 | 20 | 20 | 10 | 0 | 10 | 0 | 10 | 10 | 0 | 0 | 0 | 0 | 0 | 0 | 0 | 0 | 0 | 0 | GH |
| *Macropus antilopinus* | 100 | 0 | 0 | 0 | 0 | 0 | 0 | 0 | 0 | 0 | 0 | 0 | 0 | 0 | 0 | 0 | 0 | 0 | 0 | Gr |
| *Macropus bernardus* | 17 | 32 | 26 | 9 | 0 | 0 | 0 | 6 | 10 | 0 | 0 | 0 | 0 | 0 | 0 | 0 | 0 | 0 | 0 | SF |
| *Macropus dorsalis* | 80 | 10 | 0 | 0 | 0 | 10 | 0 | 0 | 0 | 0 | 0 | 0 | 0 | 0 | 0 | 0 | 0 | 0 | 0 | Gr |
| *Macropus eugenii* | 100 | 0 | 0 | 0 | 0 | 0 | 0 | 0 | 0 | 0 | 0 | 0 | 0 | 0 | 0 | 0 | 0 | 0 | 0 | Gr |
| *Macropus fuliginosus* | 25 | 25 | 0 | 0 | 0 | 25 | 0 | 0 | 0 | 0 | 25 | 0 | 0 | 0 | 0 | 0 | 0 | 0 | 0 | MF |
| *Macropus giganteus* | 80 | 0 | 0 | 0 | 0 | 20 | 0 | 0 | 0 | 0 | 0 | 0 | 0 | 0 | 0 | 0 | 0 | 0 | 0 | Gr |
| *Macropus irma* | 10 | 80 | 0 | 0 | 0 | 10 | 0 | 0 | 0 | 0 | 0 | 0 | 0 | 0 | 0 | 0 | 0 | 0 | 0 | Fl |
| *Macropus parma* | 50 | 0 | 0 | 0 | 0 | 50 | 0 | 0 | 0 | 0 | 0 | 0 | 0 | 0 | 0 | 0 | 0 | 0 | 0 | MF |
| *Macropus parryi* | 34 | 33 | 0 | 0 | 0 | 33 | 0 | 0 | 0 | 0 | 0 | 0 | 0 | 0 | 0 | 0 | 0 | 0 | 0 | MF |
| *Macropus robustus* | 26 | 36 | 22 | 4 | 0 | 0 | 0 | 4 | 8 | 0 | 0 | 0 | 0 | 0 | 0 | 0 | 0 | 0 | 0 | MF |
| *Macropus rufogriseus* | 50 | 0 | 0 | 0 | 0 | 50 | 0 | 0 | 0 | 0 | 0 | 0 | 0 | 0 | 0 | 0 | 0 | 0 | 0 | MF |
| *Macropus rufus* | 75 | 5 | 0 | 0 | 0 | 20 | 0 | 0 | 0 | 0 | 0 | 0 | 0 | 0 | 0 | 0 | 0 | 0 | 0 | Gr |
| *Onychogalea fraenata* | 34 | 33 | 0 | 0 | 0 | 33 | 0 | 0 | 0 | 0 | 0 | 0 | 0 | 0 | 0 | 0 | 0 | 0 | 0 | MF |
| *Onychogalea unguifera* | 34 | 0 | 33 | 0 | 0 | 33 | 0 | 0 | 0 | 0 | 0 | 0 | 0 | 0 | 0 | 0 | 0 | 0 | 0 | GH |
| *Petrogale assimilis* | 5 | 25 | 0 | 0 | 0 | 70 | 0 | 0 | 0 | 0 | 0 | 0 | 0 | 0 | 0 | 0 | 0 | 0 | 0 | GH |
| *Petrogale brachyotis* | 14 | 45 | 30 | 0 | 0 | 0 | 0 | 6 | 5 | 0 | 0 | 0 | 0 | 0 | 0 | 0 | 0 | 0 | 0 | SF |
| *Petrogale concinna* | 12 | 28 | 30 | 0 | 0 | 0 | 0 | 10 | 20 | 0 | 0 | 0 | 0 | 0 | 0 | 0 | 0 | 0 | 0 | SF |
| *Petrogale coenensis* | 20 | 40 | 0 | 0 | 0 | 20 | 0 | 0 | 0 | 20 | 0 | 0 | 0 | 0 | 0 | 0 | 0 | 0 | 0 | MF |
| *Petrogale godmani* | 0 | 100 | 0 | 0 | 0 | 0 | 0 | 0 | 0 | 0 | 0 | 0 | 0 | 0 | 0 | 0 | 0 | 0 | 0 | Fl |
| *Petrogale herberti* | 43 | 22 | 0 | 0 | 0 | 35 | 0 | 0 | 0 | 0 | 0 | 0 | 0 | 0 | 0 | 0 | 0 | 0 | 0 | MF |
| *Petrogale inornata* | 50 | 0 | 0 | 0 | 0 | 50 | 0 | 0 | 0 | 0 | 0 | 0 | 0 | 0 | 0 | 0 | 0 | 0 | 0 | MF |
| *Petrogale lateralis* | 25 | 25 | 25 | 0 | 0 | 25 | 0 | 0 | 0 | 0 | 0 | 0 | 0 | 0 | 0 | 0 | 0 | 0 | 0 | GH |

| | | | | | | | | | | | | | | | | | | | | |
|---|---|---|---|---|---|---|---|---|---|---|---|---|---|---|---|---|---|---|---|---|
| *Petrogale mareeba* | 50 | 0 | 0 | 0 | 0 | 50 | 0 | 0 | 0 | 0 | 0 | 0 | 0 | 0 | 0 | 0 | 0 | 0 | 0 | MF |
| *Petrogale penicillata* | 43 | 22 | 0 | 0 | 0 | 35 | 0 | 0 | 0 | 0 | 0 | 0 | 0 | 0 | 0 | 0 | 0 | 0 | 0 | MF |
| *Petrogale persephone* | 50 | 38 | 0 | 6 | 0 | 0 | 0 | 0 | 5 | 0 | 0 | 1 | 0 | 0 | 0 | 0 | 0 | 0 | 0 | MF |
| *Petrogale purpureicollis* | 60 | 0 | 0 | 0 | 0 | 40 | 0 | 0 | 0 | 0 | 0 | 0 | 0 | 0 | 0 | 0 | 0 | 0 | 0 | Gr |
| *Petrogale rothschildi* | 50 | 0 | 0 | 0 | 0 | 50 | 0 | 0 | 0 | 0 | 0 | 0 | 0 | 0 | 0 | 0 | 0 | 0 | 0 | MF |
| *Petrogale sharmani* | 30 | 15 | 15 | 0 | 0 | 30 | 0 | 0 | 10 | 0 | 0 | 0 | 0 | 0 | 0 | 0 | 0 | 0 | 0 | GH |
| *Petrogale xanthopus* | 50 | 50 | 0 | 0 | 0 | 0 | 0 | 0 | 0 | 0 | 0 | 0 | 0 | 0 | 0 | 0 | 0 | 0 | 0 | MF |
| *Setonix brachyurus* | 34 | 33 | 0 | 0 | 0 | 33 | 0 | 0 | 0 | 0 | 0 | 0 | 0 | 0 | 0 | 0 | 0 | 0 | 0 | MF |
| *Thylogale billardierii* | 47 | 6 | 0 | 0 | 0 | 47 | 0 | 0 | 0 | 0 | 0 | 0 | 0 | 0 | 0 | 0 | 0 | 0 | 0 | MF |
| *Thylogale browni* | 50 | 50 | 0 | 0 | 0 | 0 | 0 | 0 | 0 | 0 | 0 | 0 | 0 | 0 | 0 | 0 | 0 | 0 | 0 | MF |
| *Thylogale brunii* | 50 | 50 | 0 | 0 | 0 | 0 | 0 | 0 | 0 | 0 | 0 | 0 | 0 | 0 | 0 | 0 | 0 | 0 | 0 | MF |
| *Thylogale calabyi* | 34 | 33 | 0 | 0 | 0 | 33 | 0 | 0 | 0 | 0 | 0 | 0 | 0 | 0 | 0 | 0 | 0 | 0 | 0 | MF |
| *Thylogale lanatus* | 34 | 33 | 0 | 0 | 0 | 33 | 0 | 0 | 0 | 0 | 0 | 0 | 0 | 0 | 0 | 0 | 0 | 0 | 0 | MF |
| *Thylogale stigmatica* | 8 | 30 | 30 | 8 | 0 | 0 | 0 | 0 | 16 | 0 | 8 | 8 | 0 | 0 | 0 | 0 | 0 | 0 | 0 | SF |
| *Thylogale thetis* | 28 | 27 | 0 | 0 | 0 | 0 | 0 | 15 | 15 | 0 | 15 | 0 | 0 | 0 | 0 | 0 | 0 | 0 | 0 | MF |
| *Wallabia bicolor* | 15 | 60 | 0 | 0 | 0 | 10 | 0 | 0 | 0 | 0 | 15 | 0 | 0 | 0 | 0 | 0 | 0 | 0 | 0 | Fl |
| *Ailurops ursinus* | 80 | 0 | 10 | 10 | 0 | 0 | 0 | 0 | 0 | 0 | 0 | 0 | 0 | 0 | 0 | 0 | 0 | 0 | 0 | Gr |
| *Spilocuscus maculatus* | 0 | 40 | 40 | 10 | 0 | 0 | 0 | 0 | 0 | 0 | 0 | 0 | 0 | 0 | 0 | 10 | 0 | 0 | 0 | SF |
| *Spilocuscus rufoniger* | 0 | 25 | 14 | 13 | 0 | 0 | 0 | 0 | 38 | 0 | 0 | 0 | 5 | 0 | 0 | 5 | 0 | 0 | 0 | SF |
| *Trichosurus caninus* | 0 | 50 | 0 | 0 | 0 | 0 | 0 | 0 | 0 | 0 | 40 | 0 | 0 | 0 | 0 | 10 | 0 | 0 | 0 | Fl |
| *Trichosurus cunninghami* | 0 | 24 | 24 | 0 | 24 | 0 | 0 | 0 | 0 | 0 | 5 | 23 | 0 | 0 | 0 | 0 | 0 | 0 | 0 | SF |
| *Trichosurus vulpecula* | 0 | 50 | 14 | 13 | 0 | 0 | 0 | 0 | 13 | 0 | 0 | 0 | 5 | 0 | 0 | 5 | 0 | 0 | 0 | Fl |
| *Phascolarctos cinereus* | 0 | 70 | 15 | 0 | 0 | 0 | 0 | 0 | 0 | 0 | 15 | 0 | 0 | 0 | 0 | 0 | 0 | 0 | 0 | Fl |
| *Lasiorhinus krefftii* | 100 | 0 | 0 | 0 | 0 | 0 | 0 | 0 | 0 | 0 | 0 | 0 | 0 | 0 | 0 | 0 | 0 | 0 | 0 | Gr |
| *Lasiorhinus latifrons* | 50 | 0 | 0 | 0 | 0 | 50 | 0 | 0 | 0 | 0 | 0 | 0 | 0 | 0 | 0 | 0 | 0 | 0 | 0 | MF |
| *Vombatus ursinus* | 23 | 7 | 0 | 0 | 0 | 0 | 23 | 17 | 0 | 23 | 7 | 0 | 0 | 0 | 0 | 0 | 0 | 0 | 0 | GH |
| *Tachyglossus aculeatus* | 0 | 0 | 0 | 0 | 0 | 0 | 0 | 0 | 0 | 0 | 0 | 0 | 100 | 0 | 0 | 0 | 0 | 0 | 0 | IF |
| *Zaglossus attenboroughi* | 0 | 0 | 0 | 0 | 0 | 0 | 0 | 0 | 0 | 0 | 0 | 0 | 100 | 0 | 0 | 0 | 0 | 0 | 0 | IF |
| *Zaglossus bartoni* | 0 | 0 | 0 | 0 | 0 | 0 | 0 | 0 | 0 | 0 | 0 | 0 | 100 | 0 | 0 | 0 | 0 | 0 | 0 | IF |
| *Zaglossus bruijni* | 0 | 0 | 0 | 0 | 0 | 0 | 0 | 0 | 0 | 0 | 0 | 0 | 100 | 0 | 0 | 0 | 0 | 0 | 0 | IF |
| *Equus asinus* | 80 | 10 | 0 | 0 | 0 | 10 | 0 | 0 | 0 | 0 | 0 | 0 | 0 | 0 | 0 | 0 | 0 | 0 | 0 | Gr |

| | | | | | | | | | | | | | | | | | | | |
|---|---|---|---|---|---|---|---|---|---|---|---|---|---|---|---|---|---|---|---|
| *Equus burchellii* | 80 | 10 | 0 | 0 | 0 | 10 | 0 | 0 | 0 | 0 | 0 | 0 | 0 | 0 | 0 | 0 | 0 | 0 | 0 | Gr |
| *Equus caballus* | 100 | 0 | 0 | 0 | 0 | 0 | 0 | 0 | 0 | 0 | 0 | 0 | 0 | 0 | 0 | 0 | 0 | 0 | 0 | Gr |
| *Equus grevyi* | 80 | 10 | 0 | 0 | 0 | 10 | 0 | 0 | 0 | 0 | 0 | 0 | 0 | 0 | 0 | 0 | 0 | 0 | 0 | Gr |
| *Equus hemionus* | 80 | 0 | 0 | 0 | 0 | 10 | 0 | 0 | 0 | 0 | 10 | 0 | 0 | 0 | 0 | 0 | 0 | 0 | 0 | Gr |
| *Equus kiang* | 80 | 10 | 0 | 0 | 0 | 10 | 0 | 0 | 0 | 0 | 0 | 0 | 0 | 0 | 0 | 0 | 0 | 0 | 0 | Gr |
| *Equus zebra* | 80 | 10 | 0 | 0 | 0 | 10 | 0 | 0 | 0 | 0 | 0 | 0 | 0 | 0 | 0 | 0 | 0 | 0 | 0 | Gr |
| *Ceratotherium simum* | 100 | 0 | 0 | 0 | 0 | 0 | 0 | 0 | 0 | 0 | 0 | 0 | 0 | 0 | 0 | 0 | 0 | 0 | 0 | Gr |
| *Dicerorhinus sumatrensis* | 0 | 40 | 20 | 0 | 0 | 0 | 0 | 0 | 20 | 0 | 20 | 0 | 0 | 0 | 0 | 0 | 0 | 0 | 0 | SF |
| *Diceros bicornis* | 20 | 40 | 0 | 0 | 0 | 20 | 0 | 0 | 0 | 0 | 20 | 0 | 0 | 0 | 0 | 0 | 0 | 0 | 0 | MF |
| *Rhinoceros sondaicus* | 0 | 60 | 20 | 0 | 0 | 20 | 0 | 0 | 0 | 0 | 0 | 0 | 0 | 0 | 0 | 0 | 0 | 0 | 0 | Fl |
| *Rhinoceros unicornis* | 50 | 20 | 20 | 0 | 0 | 0 | 0 | 0 | 0 | 10 | 0 | 0 | 0 | 0 | 0 | 0 | 0 | 0 | 0 | MF |
| *Tapirus bairdii* | 10 | 60 | 10 | 10 | 0 | 0 | 0 | 0 | 0 | 10 | 0 | 0 | 0 | 0 | 0 | 0 | 0 | 0 | 0 | Fl |
| *Tapirus indicus* | 0 | 20 | 20 | 0 | 0 | 8 | 8 | 8 | 0 | 8 | 28 | 0 | 0 | 0 | 0 | 0 | 0 | 0 | 0 | GH |
| *Tapirus pinchaque* | 40 | 40 | 20 | 0 | 0 | 0 | 0 | 0 | 0 | 0 | 0 | 0 | 0 | 0 | 0 | 0 | 0 | 0 | 0 | MF |
| *Tapirus terrestris* | 20 | 40 | 20 | 0 | 0 | 0 | 0 | 0 | 0 | 20 | 0 | 0 | 0 | 0 | 0 | 0 | 0 | 0 | 0 | GH |
| *Manis crassicaudata* | 0 | 0 | 0 | 0 | 0 | 0 | 0 | 0 | 0 | 0 | 0 | 0 | 100 | 0 | 0 | 0 | 0 | 0 | 0 | IF |
| *Manis culionensis* | 0 | 0 | 0 | 0 | 0 | 0 | 0 | 0 | 0 | 0 | 0 | 0 | 100 | 0 | 0 | 0 | 0 | 0 | 0 | IF |
| *Manis gigantea* | 0 | 0 | 0 | 0 | 0 | 0 | 0 | 0 | 0 | 0 | 0 | 0 | 100 | 0 | 0 | 0 | 0 | 0 | 0 | IF |
| *Manis javanica* | 0 | 0 | 0 | 0 | 0 | 0 | 0 | 0 | 0 | 0 | 0 | 0 | 100 | 0 | 0 | 0 | 0 | 0 | 0 | IF |
| *Manis pentadactyla* | 0 | 0 | 0 | 0 | 0 | 0 | 0 | 0 | 0 | 0 | 0 | 0 | 100 | 0 | 0 | 0 | 0 | 0 | 0 | IF |
| *Manis temminckii* | 0 | 0 | 0 | 0 | 0 | 0 | 0 | 0 | 0 | 0 | 0 | 0 | 100 | 0 | 0 | 0 | 0 | 0 | 0 | IF |
| *Manis tricuspis* | 0 | 0 | 0 | 0 | 0 | 0 | 0 | 0 | 0 | 0 | 0 | 0 | 100 | 0 | 0 | 0 | 0 | 0 | 0 | IF |
| *Bradypus pygmaeus* | 0 | 100 | 0 | 0 | 0 | 0 | 0 | 0 | 0 | 0 | 0 | 0 | 0 | 0 | 0 | 0 | 0 | 0 | 0 | Fl |
| *Bradypus torquatus* | 0 | 100 | 0 | 0 | 0 | 0 | 0 | 0 | 0 | 0 | 0 | 0 | 0 | 0 | 0 | 0 | 0 | 0 | 0 | Fl |
| *Bradypus tridactylus* | 0 | 100 | 0 | 0 | 0 | 0 | 0 | 0 | 0 | 0 | 0 | 0 | 0 | 0 | 0 | 0 | 0 | 0 | 0 | Fl |
| *Bradypus variegatus* | 0 | 40 | 30 | 30 | 0 | 0 | 0 | 0 | 0 | 0 | 0 | 0 | 0 | 0 | 0 | 0 | 0 | 0 | 0 | SF |
| *Choloepus didactylus* | 0 | 44 | 44 | 0 | 0 | 0 | 0 | 10 | 0 | 0 | 0 | 0 | 2 | 0 | 0 | 0 | 0 | 0 | 0 | SF |
| *Choloepus hoffmanni* | 0 | 55 | 15 | 15 | 0 | 0 | 0 | 0 | 0 | 0 | 0 | 0 | 5 | 0 | 5 | 5 | 0 | 0 | 0 | Fl |
| *Myrmecophaga tridactyla* | 0 | 0 | 0 | 0 | 0 | 0 | 0 | 0 | 0 | 0 | 0 | 0 | 100 | 0 | 0 | 0 | 0 | 0 | 0 | IF |
| *Tamandua mexicana* | 0 | 0 | 0 | 0 | 0 | 0 | 0 | 0 | 0 | 0 | 0 | 0 | 100 | 0 | 0 | 0 | 0 | 0 | 0 | IF |
| *Tamandua tetradactyla* | 0 | 0 | 0 | 0 | 0 | 0 | 0 | 0 | 0 | 0 | 0 | 0 | 100 | 0 | 0 | 0 | 0 | 0 | 0 | IF |

| | | | | | | | | | | | | | | | | | | | | |
|---|---|---|---|---|---|---|---|---|---|---|---|---|---|---|---|---|---|---|---|---|
| *Alouatta belzebul* | 0 | 85 | 0 | 5 | 0 | 0 | 0 | 0 | 0 | 0 | 10 | 0 | 0 | 0 | 0 | 0 | 0 | 0 | 0 | Fl |
| *Alouatta caraya* | 0 | 80 | 10 | 10 | 0 | 0 | 0 | 0 | 0 | 0 | 0 | 0 | 0 | 0 | 0 | 0 | 0 | 0 | 0 | Fl |
| *Alouatta coibensis* | 0 | 70 | 15 | 15 | 0 | 0 | 0 | 0 | 0 | 0 | 0 | 0 | 0 | 0 | 0 | 0 | 0 | 0 | 0 | Fl |
| *Alouatta guariba* | 0 | 80 | 10 | 10 | 0 | 0 | 0 | 0 | 0 | 0 | 0 | 0 | 0 | 0 | 0 | 0 | 0 | 0 | 0 | Fl |
| *Alouatta macconnelli* | 0 | 55 | 15 | 15 | 0 | 0 | 0 | 0 | 15 | 0 | 0 | 0 | 0 | 0 | 0 | 0 | 0 | 0 | 0 | Fl |
| *Alouatta nigerrima* | 0 | 55 | 15 | 15 | 0 | 0 | 0 | 0 | 15 | 0 | 0 | 0 | 0 | 0 | 0 | 0 | 0 | 0 | 0 | Fl |
| *Alouatta palliata* | 0 | 45 | 39 | 16 | 0 | 0 | 0 | 0 | 0 | 0 | 0 | 0 | 0 | 0 | 0 | 0 | 0 | 0 | 0 | SF |
| *Alouatta pigra* | 0 | 43 | 43 | 14 | 0 | 0 | 0 | 0 | 0 | 0 | 0 | 0 | 0 | 0 | 0 | 0 | 0 | 0 | 0 | SF |
| *Alouatta sara* | 0 | 55 | 15 | 15 | 0 | 0 | 0 | 0 | 15 | 0 | 0 | 0 | 0 | 0 | 0 | 0 | 0 | 0 | 0 | Fl |
| *Alouatta seniculus* | 0 | 43 | 43 | 14 | 0 | 0 | 0 | 0 | 0 | 0 | 0 | 0 | 0 | 0 | 0 | 0 | 0 | 0 | 0 | SF |
| *Ateles belzebuth* | 0 | 10 | 84 | 0 | 0 | 0 | 0 | 0 | 2 | 0 | 2 | 0 | 2 | 0 | 0 | 0 | 0 | 0 | 0 | Fr |
| *Ateles chamek* | 0 | 20 | 60 | 4 | 0 | 0 | 0 | 0 | 0 | 0 | 0 | 0 | 8 | 0 | 0 | 8 | 0 | 0 | 0 | Fr |
| *Ateles fusciceps* | 0 | 40 | 40 | 0 | 0 | 0 | 0 | 0 | 12 | 0 | 0 | 0 | 6 | 0 | 0 | 2 | 0 | 0 | 0 | SF |
| *Ateles geoffroyi* | 0 | 4 | 82 | 4 | 0 | 0 | 0 | 0 | 4 | 0 | 4 | 0 | 2 | 0 | 0 | 0 | 0 | 0 | 0 | Fr |
| *Ateles hybridus* | 0 | 5 | 85 | 5 | 0 | 0 | 0 | 0 | 5 | 0 | 0 | 0 | 0 | 0 | 0 | 0 | 0 | 0 | 0 | Fr |
| *Ateles marginatus* | 0 | 10 | 60 | 10 | 0 | 0 | 0 | 5 | 0 | 0 | 10 | 0 | 5 | 0 | 0 | 0 | 0 | 0 | 0 | Fr |
| *Ateles paniscus* | 0 | 10 | 83 | 3 | 0 | 0 | 0 | 1 | 2 | 0 | 0 | 1 | 0 | 0 | 0 | 0 | 0 | 0 | 0 | Fr |
| *Brachyteles arachnoides* | 0 | 50 | 30 | 10 | 0 | 0 | 0 | 0 | 10 | 0 | 0 | 0 | 0 | 0 | 0 | 0 | 0 | 0 | 0 | Fl |
| *Brachyteles hypoxanthus* | 0 | 50 | 30 | 10 | 0 | 0 | 0 | 0 | 10 | 0 | 0 | 0 | 0 | 0 | 0 | 0 | 0 | 0 | 0 | Fl |
| *Lagothrix cana* | 0 | 15 | 80 | 0 | 0 | 0 | 0 | 0 | 5 | 0 | 0 | 0 | 0 | 0 | 0 | 0 | 0 | 0 | 0 | Fr |
| *Lagothrix lagotricha* | 0 | 10 | 75 | 0 | 0 | 0 | 0 | 0 | 10 | 0 | 0 | 0 | 5 | 0 | 0 | 0 | 0 | 0 | 0 | Fr |
| *Lagothrix lugens* | 0 | 14 | 83 | 2 | 0 | 0 | 0 | 0 | 0 | 0 | 1 | 0 | 0 | 0 | 0 | 0 | 0 | 0 | 0 | Fr |
| *Lagothrix poeppigii* | 0 | 10 | 73 | 5 | 0 | 0 | 0 | 0 | 0 | 0 | 0 | 2 | 10 | 0 | 0 | 0 | 0 | 0 | 0 | Fr |
| *Oreonax flavicauda* | 0 | 10 | 73 | 7 | 0 | 0 | 0 | 0 | 0 | 0 | 0 | 0 | 10 | 0 | 0 | 0 | 0 | 0 | 0 | Fr |
| *Allenopithecus nigroviridis* | 0 | 40 | 40 | 0 | 0 | 0 | 0 | 0 | 0 | 0 | 0 | 0 | 20 | 0 | 0 | 0 | 0 | 0 | 0 | SF |
| *Cercocebus agilis* | 0 | 10 | 30 | 0 | 0 | 0 | 0 | 0 | 40 | 0 | 0 | 10 | 5 | 0 | 0 | 5 | 0 | 0 | 0 | Om |
| *Cercocebus atys* | 0 | 5 | 30 | 5 | 0 | 0 | 0 | 0 | 50 | 0 | 0 | 0 | 5 | 0 | 0 | 5 | 0 | 0 | 0 | Om |
| *Cercocebus chrysogaster* | 0 | 10 | 30 | 0 | 0 | 0 | 0 | 0 | 40 | 0 | 0 | 10 | 5 | 0 | 0 | 5 | 0 | 0 | 0 | Om |
| *Cercocebus galeritus* | 0 | 10 | 35 | 0 | 0 | 0 | 0 | 0 | 35 | 0 | 0 | 0 | 10 | 0 | 0 | 10 | 0 | 0 | 0 | Om |
| *Cercocebus sanjei* | 0 | 9 | 65 | 0 | 0 | 0 | 0 | 0 | 16 | 0 | 0 | 0 | 5 | 0 | 0 | 5 | 0 | 0 | 0 | Fr |
| *Cercocebus torquatus* | 0 | 10 | 40 | 0 | 0 | 0 | 0 | 0 | 40 | 0 | 0 | 0 | 5 | 0 | 0 | 5 | 0 | 0 | 0 | Om |

| | | | | | | | | | | | | | | | | | | | | |
|---|---|---|---|---|---|---|---|---|---|---|---|---|---|---|---|---|---|---|---|---|
| *Cercopithecus albogularis* | 0 | 10 | 60 | 0 | 0 | 0 | 0 | 0 | 10 | 0 | 0 | 0 | 10 | 0 | 0 | 10 | 0 | 0 | 0 | Fr |
| *Cercopithecus ascanius* | 0 | 15 | 60 | 15 | 0 | 0 | 0 | 0 | 0 | 0 | 0 | 0 | 10 | 0 | 0 | 0 | 0 | 0 | 0 | Fr |
| *Cercopithecus campbelli* | 0 | 10 | 60 | 0 | 0 | 0 | 0 | 0 | 10 | 0 | 0 | 0 | 10 | 0 | 0 | 10 | 0 | 0 | 0 | Fr |
| *Cercopithecus cephus* | 0 | 10 | 60 | 0 | 0 | 0 | 0 | 0 | 10 | 0 | 0 | 0 | 10 | 0 | 0 | 10 | 0 | 0 | 0 | Fr |
| *Cercopithecus denti* | 0 | 15 | 65 | 0 | 0 | 0 | 0 | 0 | 15 | 0 | 0 | 0 | 5 | 0 | 0 | 0 | 0 | 0 | 0 | Fr |
| *Cercopithecus diana* | 0 | 10 | 40 | 10 | 0 | 0 | 0 | 0 | 0 | 0 | 0 | 0 | 40 | 0 | 0 | 0 | 0 | 0 | 0 | Om |
| *Cercopithecus doggetti* | 0 | 30 | 65 | 0 | 0 | 0 | 0 | 0 | 0 | 0 | 0 | 0 | 5 | 0 | 0 | 0 | 0 | 0 | 0 | Fr |
| *Cercopithecus dryas* | 0 | 15 | 60 | 15 | 0 | 0 | 0 | 0 | 0 | 0 | 0 | 0 | 10 | 0 | 0 | 0 | 0 | 0 | 0 | Fr |
| *Cercopithecus erythrogaster* | 0 | 15 | 70 | 0 | 0 | 0 | 0 | 0 | 0 | 0 | 0 | 0 | 15 | 0 | 0 | 0 | 0 | 0 | 0 | Fr |
| *Cercopithecus erythrotis* | 0 | 15 | 60 | 15 | 0 | 0 | 0 | 0 | 0 | 0 | 0 | 0 | 10 | 0 | 0 | 0 | 0 | 0 | 0 | Fr |
| *Cercopithecus hamlyni* | 0 | 90 | 10 | 0 | 0 | 0 | 0 | 0 | 0 | 0 | 0 | 0 | 0 | 0 | 0 | 0 | 0 | 0 | 0 | Fl |
| *Cercopithecus kandti* | 0 | 40 | 30 | 20 | 0 | 0 | 0 | 0 | 0 | 0 | 0 | 0 | 10 | 0 | 0 | 0 | 0 | 0 | 0 | SF |
| *Cercopithecus lhoesti* | 0 | 19 | 19 | 0 | 0 | 19 | 0 | 19 | 0 | 0 | 0 | 18 | 3 | 0 | 0 | 3 | 0 | 0 | 0 | GH |
| *Cercopithecus lowei* | 0 | 20 | 70 | 0 | 0 | 0 | 0 | 0 | 0 | 0 | 0 | 0 | 10 | 0 | 0 | 0 | 0 | 0 | 0 | Fr |
| *Cercopithecus mitis* | 0 | 30 | 45 | 5 | 0 | 0 | 0 | 0 | 10 | 0 | 0 | 0 | 10 | 0 | 0 | 0 | 0 | 0 | 0 | SF |
| *Cercopithecus mona* | 0 | 15 | 60 | 0 | 0 | 0 | 0 | 0 | 0 | 0 | 0 | 0 | 25 | 0 | 0 | 0 | 0 | 0 | 0 | Fr |
| *Cercopithecus neglectus* | 0 | 15 | 60 | 15 | 0 | 0 | 0 | 0 | 0 | 0 | 0 | 0 | 10 | 0 | 0 | 0 | 0 | 0 | 0 | Fr |
| *Cercopithecus nictitans* | 0 | 8 | 48 | 8 | 0 | 0 | 0 | 0 | 28 | 0 | 0 | 0 | 8 | 0 | 0 | 0 | 0 | 0 | 0 | Fr |
| *Cercopithecus petaurista* | 0 | 50 | 40 | 0 | 0 | 0 | 0 | 0 | 0 | 0 | 0 | 0 | 10 | 0 | 0 | 0 | 0 | 0 | 0 | SF |
| *Cercopithecus pogonias* | 0 | 10 | 60 | 10 | 0 | 0 | 0 | 0 | 10 | 0 | 0 | 0 | 10 | 0 | 0 | 0 | 0 | 0 | 0 | Fr |
| *Cercopithecus preussi* | 0 | 30 | 40 | 0 | 0 | 0 | 0 | 0 | 0 | 0 | 0 | 0 | 30 | 0 | 0 | 0 | 0 | 0 | 0 | SF |
| *Cercopithecus roloway* | 0 | 15 | 40 | 15 | 0 | 0 | 0 | 0 | 15 | 0 | 0 | 0 | 15 | 0 | 0 | 0 | 0 | 0 | 0 | Om |
| *Cercopithecus sclateri* | 0 | 20 | 45 | 0 | 0 | 0 | 0 | 0 | 20 | 0 | 0 | 0 | 15 | 0 | 0 | 0 | 0 | 0 | 0 | Om |
| *Cercopithecus solatus* | 0 | 20 | 40 | 0 | 0 | 0 | 0 | 0 | 20 | 0 | 0 | 0 | 10 | 0 | 0 | 10 | 0 | 0 | 0 | Om |
| *Cercopithecus wolfi* | 0 | 10 | 60 | 10 | 0 | 0 | 0 | 0 | 10 | 0 | 0 | 0 | 10 | 0 | 0 | 0 | 0 | 0 | 0 | Fr |
| *Chlorocebus aethiops* | 0 | 20 | 40 | 0 | 0 | 0 | 0 | 0 | 20 | 0 | 0 | 0 | 10 | 0 | 0 | 10 | 0 | 0 | 0 | Om |
| *Chlorocebus cynosuros* | 0 | 15 | 35 | 15 | 0 | 0 | 0 | 0 | 15 | 0 | 0 | 0 | 10 | 0 | 0 | 10 | 0 | 0 | 0 | Om |
| *Chlorocebus djamdjamensis* | 0 | 20 | 35 | 20 | 0 | 0 | 0 | 0 | 15 | 0 | 0 | 0 | 10 | 0 | 0 | 0 | 0 | 0 | 0 | SF |
| *Chlorocebus pygerythrus* | 0 | 20 | 35 | 20 | 0 | 0 | 0 | 0 | 15 | 0 | 0 | 0 | 5 | 0 | 0 | 5 | 0 | 0 | 0 | SF |
| *Chlorocebus sabaeus* | 0 | 25 | 35 | 20 | 0 | 0 | 0 | 0 | 15 | 0 | 0 | 0 | 5 | 0 | 0 | 0 | 0 | 0 | 0 | SF |
| *Chlorocebus tantalus* | 0 | 20 | 35 | 20 | 0 | 0 | 0 | 0 | 15 | 0 | 0 | 0 | 5 | 0 | 0 | 5 | 0 | 0 | 0 | SF |

| | | | | | | | | | | | | | | | | | | | | |
|---|---|---|---|---|---|---|---|---|---|---|---|---|---|---|---|---|---|---|---|---|
| *Colobus angolensis* | 0 | 13 | 35 | 13 | 0 | 0 | 0 | 0 | 0 | 13 | 13 | 0 | 13 | 0 | 0 | 0 | 0 | 0 | 0 | Om |
| *Colobus guereza* | 0 | 85 | 10 | 0 | 0 | 0 | 0 | 0 | 0 | 5 | 0 | 0 | 0 | 0 | 0 | 0 | 0 | 0 | 0 | Fl |
| *Colobus polykomos* | 0 | 80 | 10 | 10 | 0 | 0 | 0 | 0 | 0 | 0 | 0 | 0 | 0 | 0 | 0 | 0 | 0 | 0 | 0 | Fl |
| *Colobus satanas* | 0 | 10 | 10 | 10 | 0 | 0 | 0 | 0 | 70 | 0 | 0 | 0 | 0 | 0 | 0 | 0 | 0 | 0 | 0 | Om |
| *Colobus vellerosus* | 0 | 45 | 10 | 0 | 0 | 0 | 0 | 0 | 40 | 0 | 0 | 0 | 5 | 0 | 0 | 0 | 0 | 0 | 0 | SF |
| *Erythrocebus patas* | 0 | 10 | 40 | 0 | 0 | 0 | 0 | 10 | 0 | 0 | 0 | 0 | 30 | 0 | 0 | 10 | 0 | 0 | 0 | Om |
| *Lophocebus albigena* | 0 | 8 | 25 | 8 | 0 | 0 | 0 | 0 | 43 | 0 | 0 | 0 | 8 | 0 | 0 | 8 | 0 | 0 | 0 | Om |
| *Lophocebus aterrimus* | 0 | 0 | 40 | 0 | 0 | 0 | 0 | 0 | 30 | 0 | 0 | 0 | 15 | 0 | 0 | 15 | 0 | 0 | 0 | Om |
| *Lophocebus opdenboschi* | 0 | 0 | 40 | 0 | 0 | 0 | 0 | 0 | 30 | 0 | 0 | 0 | 15 | 0 | 0 | 15 | 0 | 0 | 0 | Om |
| *Macaca arctoides* | 0 | 10 | 40 | 10 | 0 | 0 | 0 | 10 | 10 | 0 | 0 | 0 | 10 | 0 | 0 | 10 | 0 | 0 | 0 | Om |
| *Macaca assamensis* | 0 | 15 | 35 | 15 | 0 | 0 | 0 | 5 | 15 | 0 | 0 | 0 | 10 | 0 | 0 | 5 | 0 | 0 | 0 | Om |
| *Macaca cyclopis* | 0 | 15 | 40 | 0 | 0 | 0 | 0 | 0 | 15 | 0 | 0 | 0 | 15 | 0 | 0 | 15 | 0 | 0 | 0 | Om |
| *Macaca fascicularis* | 0 | 5 | 73 | 5 | 0 | 0 | 0 | 5 | 0 | 0 | 5 | 0 | 0 | 3 | 0 | 4 | 0 | 0 | 0 | Fr |
| *Macaca fuscata* | 0 | 5 | 50 | 0 | 0 | 5 | 0 | 6 | 16 | 0 | 3 | 5 | 5 | 0 | 0 | 5 | 0 | 0 | 0 | Fr |
| *Macaca hecki* | 0 | 5 | 75 | 5 | 0 | 0 | 0 | 5 | 0 | 0 | 5 | 0 | 0 | 0 | 0 | 5 | 0 | 0 | 0 | Fr |
| *Macaca leonina* | 0 | 8 | 55 | 0 | 0 | 0 | 0 | 5 | 8 | 0 | 0 | 8 | 8 | 0 | 0 | 8 | 0 | 0 | 0 | Fr |
| *Macaca maura* | 0 | 18 | 28 | 0 | 0 | 0 | 0 | 0 | 18 | 0 | 0 | 0 | 18 | 0 | 0 | 18 | 0 | 0 | 0 | Om |
| *Macaca mulatta* | 0 | 10 | 25 | 9 | 0 | 9 | 0 | 6 | 12 | 0 | 8 | 5 | 8 | 3 | 0 | 5 | 0 | 0 | 0 | Om |
| *Macaca nemestrina* | 0 | 6 | 50 | 3 | 0 | 5 | 0 | 6 | 12 | 0 | 0 | 6 | 6 | 0 | 0 | 6 | 0 | 0 | 0 | Fr |
| *Macaca nigra* | 0 | 10 | 60 | 10 | 0 | 0 | 0 | 0 | 0 | 0 | 0 | 0 | 10 | 0 | 0 | 10 | 0 | 0 | 0 | Fr |
| *Macaca nigrescens* | 0 | 10 | 70 | 10 | 0 | 0 | 0 | 0 | 0 | 0 | 0 | 0 | 10 | 0 | 0 | 0 | 0 | 0 | 0 | Fr |
| *Macaca ochreata* | 0 | 25 | 55 | 0 | 0 | 0 | 0 | 0 | 5 | 0 | 0 | 0 | 15 | 0 | 0 | 0 | 0 | 0 | 0 | Fr |
| *Macaca pagensis* | 0 | 10 | 50 | 5 | 0 | 2 | 0 | 4 | 10 | 0 | 2 | 2 | 8 | 0 | 0 | 7 | 0 | 0 | 0 | Fr |
| *Macaca radiata* | 0 | 15 | 40 | 0 | 0 | 0 | 0 | 0 | 20 | 0 | 0 | 0 | 20 | 0 | 0 | 5 | 0 | 0 | 0 | Om |
| *Macaca siberu* | 0 | 5 | 85 | 5 | 0 | 0 | 0 | 0 | 5 | 0 | 0 | 0 | 0 | 0 | 0 | 0 | 0 | 0 | 0 | Fr |
| *Macaca silenus* | 0 | 10 | 60 | 10 | 0 | 0 | 0 | 0 | 0 | 0 | 0 | 10 | 5 | 0 | 0 | 5 | 0 | 0 | 0 | Fr |
| *Macaca sinica* | 0 | 15 | 60 | 15 | 0 | 0 | 0 | 0 | 0 | 0 | 0 | 0 | 5 | 0 | 0 | 5 | 0 | 0 | 0 | Fr |
| *Macaca sylvanus* | 0 | 10 | 15 | 0 | 0 | 10 | 0 | 10 | 22 | 0 | 7 | 10 | 10 | 0 | 0 | 6 | 0 | 0 | 0 | Om |
| *Macaca thibetana* | 24 | 24 | 24 | 7 | 0 | 0 | 0 | 7 | 7 | 0 | 0 | 0 | 7 | 0 | 0 | 0 | 0 | 0 | 0 | GH |
| *Macaca tonkeana* | 0 | 15 | 55 | 0 | 0 | 0 | 0 | 0 | 15 | 0 | 0 | 0 | 15 | 0 | 0 | 0 | 0 | 0 | 0 | Fr |
| *Mandrillus leucophaeus* | 0 | 10 | 50 | 0 | 0 | 10 | 0 | 10 | 0 | 0 | 0 | 0 | 10 | 0 | 0 | 10 | 0 | 0 | 0 | Fr |

| | | | | | | | | | | | | | | | | | | | |
|---|---|---|---|---|---|---|---|---|---|---|---|---|---|---|---|---|---|---|---|
| *Mandrillus sphinx* | 0 | 8 | 45 | 3 | 0 | 0 | 0 | 0 | 24 | 0 | 2 | 2 | 5 | 0 | 0 | 5 | 3 | 3 | 0 | Fr |
| *Miopithecus ogouensis* | 0 | 5 | 40 | 5 | 0 | 0 | 0 | 0 | 5 | 0 | 0 | 0 | 35 | 0 | 0 | 10 | 0 | 0 | 0 | Om |
| *Nasalis larvatus* | 0 | 35 | 35 | 12 | 0 | 0 | 0 | 0 | 13 | 0 | 0 | 0 | 5 | 0 | 0 | 0 | 0 | 0 | 0 | SF |
| *Papio anubis* | 10 | 6 | 6 | 6 | 6 | 0 | 0 | 24 | 6 | 0 | 6 | 6 | 6 | 0 | 0 | 6 | 6 | 6 | 0 | GH |
| *Papio cynocephalus* | 7 | 7 | 20 | 7 | 0 | 0 | 0 | 14 | 7 | 7 | 7 | 0 | 8 | 0 | 0 | 8 | 8 | 0 | 0 | Om |
| *Papio hamadryas* | 20 | 0 | 15 | 9 | 0 | 9 | 0 | 18 | 9 | 0 | 0 | 0 | 10 | 0 | 0 | 10 | 0 | 0 | 0 | GH |
| *Papio papio* | 14 | 16 | 16 | 0 | 0 | 0 | 0 | 30 | 0 | 0 | 0 | 0 | 14 | 0 | 0 | 10 | 0 | 0 | 0 | GH |
| *Papio ursinus* | 7 | 7 | 20 | 7 | 0 | 0 | 0 | 14 | 7 | 7 | 7 | 0 | 8 | 0 | 8 | 8 | 0 | 0 | 0 | Om |
| *Piliocolobus badius* | 0 | 60 | 25 | 5 | 0 | 0 | 0 | 0 | 10 | 0 | 0 | 0 | 0 | 0 | 0 | 0 | 0 | 0 | 0 | Fl |
| *Piliocolobus foai* | 0 | 80 | 20 | 0 | 0 | 0 | 0 | 0 | 0 | 0 | 0 | 0 | 0 | 0 | 0 | 0 | 0 | 0 | 0 | Fl |
| *Piliocolobus gordonorum* | 0 | 80 | 20 | 0 | 0 | 0 | 0 | 0 | 0 | 0 | 0 | 0 | 0 | 0 | 0 | 0 | 0 | 0 | 0 | Fl |
| *Piliocolobus kirkii* | 0 | 60 | 25 | 15 | 0 | 0 | 0 | 0 | 0 | 0 | 0 | 0 | 0 | 0 | 0 | 0 | 0 | 0 | 0 | Fl |
| *Piliocolobus pennantii* | 0 | 70 | 15 | 0 | 0 | 0 | 0 | 0 | 15 | 0 | 0 | 0 | 0 | 0 | 0 | 0 | 0 | 0 | 0 | Fl |
| *Piliocolobus preussi* | 0 | 80 | 20 | 0 | 0 | 0 | 0 | 0 | 0 | 0 | 0 | 0 | 0 | 0 | 0 | 0 | 0 | 0 | 0 | Fl |
| *Piliocolobus rufomitratus* | 0 | 65 | 15 | 10 | 0 | 0 | 0 | 0 | 10 | 0 | 0 | 0 | 0 | 0 | 0 | 0 | 0 | 0 | 0 | Fl |
| *Piliocolobus tephrosceles* | 0 | 85 | 5 | 0 | 0 | 0 | 0 | 0 | 5 | 0 | 5 | 0 | 0 | 0 | 0 | 0 | 0 | 0 | 0 | Fl |
| *Piliocolobus tholloni* | 0 | 80 | 20 | 0 | 0 | 0 | 0 | 0 | 0 | 0 | 0 | 0 | 0 | 0 | 0 | 0 | 0 | 0 | 0 | Fl |
| *Presbytis chrysomelas* | 0 | 40 | 30 | 15 | 0 | 0 | 0 | 0 | 15 | 0 | 0 | 0 | 0 | 0 | 0 | 0 | 0 | 0 | 0 | SF |
| *Presbytis comata* | 0 | 80 | 5 | 5 | 0 | 0 | 0 | 0 | 5 | 0 | 0 | 5 | 0 | 0 | 0 | 0 | 0 | 0 | 0 | Fl |
| *Presbytis femoralis* | 0 | 40 | 40 | 10 | 0 | 0 | 0 | 0 | 10 | 0 | 0 | 0 | 0 | 0 | 0 | 0 | 0 | 0 | 0 | SF |
| *Presbytis frontata* | 0 | 60 | 20 | 10 | 0 | 0 | 0 | 0 | 10 | 0 | 0 | 0 | 0 | 0 | 0 | 0 | 0 | 0 | 0 | Fl |
| *Presbytis hosei* | 0 | 60 | 20 | 10 | 0 | 0 | 0 | 0 | 10 | 0 | 0 | 0 | 0 | 0 | 0 | 0 | 0 | 0 | 0 | Fl |
| *Presbytis melalophos* | 0 | 60 | 20 | 10 | 0 | 0 | 0 | 0 | 10 | 0 | 0 | 0 | 0 | 0 | 0 | 0 | 0 | 0 | 0 | Fl |
| *Presbytis natunae* | 0 | 60 | 20 | 10 | 0 | 0 | 0 | 0 | 10 | 0 | 0 | 0 | 0 | 0 | 0 | 0 | 0 | 0 | 0 | Fl |
| *Presbytis potenziani* | 0 | 40 | 20 | 20 | 0 | 0 | 0 | 0 | 20 | 0 | 0 | 0 | 0 | 0 | 0 | 0 | 0 | 0 | 0 | SF |
| *Presbytis rubicunda* | 0 | 40 | 30 | 0 | 0 | 0 | 0 | 0 | 30 | 0 | 0 | 0 | 0 | 0 | 0 | 0 | 0 | 0 | 0 | SF |
| *Presbytis siamensis* | 0 | 40 | 40 | 10 | 0 | 0 | 0 | 0 | 10 | 0 | 0 | 0 | 0 | 0 | 0 | 0 | 0 | 0 | 0 | SF |
| *Presbytis thomasi* | 0 | 30 | 30 | 10 | 0 | 0 | 0 | 0 | 10 | 0 | 5 | 5 | 5 | 0 | 0 | 5 | 0 | 0 | 0 | SF |
| *Procolobus verus* | 0 | 70 | 10 | 10 | 0 | 0 | 0 | 0 | 10 | 0 | 0 | 0 | 0 | 0 | 0 | 0 | 0 | 0 | 0 | Fl |
| *Pygathrix cinerea* | 0 | 40 | 40 | 20 | 0 | 0 | 0 | 0 | 0 | 0 | 0 | 0 | 0 | 0 | 0 | 0 | 0 | 0 | 0 | SF |
| *Pygathrix nemaeus* | 0 | 70 | 10 | 10 | 0 | 0 | 0 | 0 | 10 | 0 | 0 | 0 | 0 | 0 | 0 | 0 | 0 | 0 | 0 | Fl |

| | | | | | | | | | | | | | | | | | | | | |
|---|---|---|---|---|---|---|---|---|---|---|---|---|---|---|---|---|---|---|---|---|
| *Pygathrix nigripes* | 0 | 70 | 10 | 10 | 0 | 0 | 0 | 0 | 10 | 0 | 0 | 0 | 0 | 0 | 0 | 0 | 0 | 0 | 0 | Fl |
| *Rhinopithecus avunculus* | 0 | 25 | 25 | 25 | 0 | 0 | 0 | 0 | 25 | 0 | 0 | 0 | 0 | 0 | 0 | 0 | 0 | 0 | 0 | SF |
| *Rhinopithecus bieti* | 0 | 50 | 5 | 0 | 35 | 0 | 0 | 0 | 5 | 0 | 5 | 0 | 0 | 0 | 0 | 0 | 0 | 0 | 0 | Fl |
| *Rhinopithecus brelichi* | 0 | 25 | 25 | 25 | 0 | 0 | 0 | 0 | 25 | 0 | 0 | 0 | 0 | 0 | 0 | 0 | 0 | 0 | 0 | SF |
| *Rhinopithecus roxellana* | 12 | 56 | 0 | 0 | 8 | 0 | 0 | 8 | 8 | 0 | 8 | 0 | 0 | 0 | 0 | 0 | 0 | 0 | 0 | Fl |
| *Semnopithecus ajax* | 0 | 80 | 0 | 0 | 0 | 0 | 0 | 0 | 10 | 0 | 10 | 0 | 0 | 0 | 0 | 0 | 0 | 0 | 0 | Fl |
| *Semnopithecus dussumieri* | 0 | 73 | 0 | 0 | 0 | 8 | 0 | 0 | 8 | 0 | 8 | 0 | 3 | 0 | 0 | 0 | 0 | 0 | 0 | Fl |
| *Semnopithecus entellus* | 0 | 60 | 22 | 10 | 0 | 0 | 0 | 0 | 0 | 0 | 5 | 0 | 3 | 0 | 0 | 0 | 0 | 0 | 0 | Fl |
| *Semnopithecus hector* | 0 | 70 | 20 | 5 | 0 | 0 | 0 | 0 | 5 | 0 | 0 | 0 | 0 | 0 | 0 | 0 | 0 | 0 | 0 | Fl |
| *Semnopithecus hypoleucos* | 0 | 80 | 10 | 0 | 0 | 0 | 0 | 0 | 10 | 0 | 0 | 0 | 0 | 0 | 0 | 0 | 0 | 0 | 0 | Fl |
| *Semnopithecus priam* | 0 | 80 | 10 | 0 | 0 | 0 | 0 | 0 | 10 | 0 | 0 | 0 | 0 | 0 | 0 | 0 | 0 | 0 | 0 | Fl |
| *Semnopithecus schistaceus* | 0 | 80 | 10 | 0 | 0 | 0 | 0 | 0 | 10 | 0 | 0 | 0 | 0 | 0 | 0 | 0 | 0 | 0 | 0 | Fl |
| *Simias concolor* | 0 | 80 | 10 | 10 | 0 | 0 | 0 | 0 | 0 | 0 | 0 | 0 | 0 | 0 | 0 | 0 | 0 | 0 | 0 | Fl |
| *Theropithecus gelada* | 45 | 5 | 5 | 5 | 0 | 0 | 0 | 20 | 10 | 0 | 10 | 0 | 0 | 0 | 0 | 0 | 0 | 0 | 0 | MF |
| *Trachypithecus auratus* | 0 | 25 | 25 | 25 | 0 | 0 | 0 | 0 | 17 | 0 | 0 | 0 | 8 | 0 | 0 | 0 | 0 | 0 | 0 | SF |
| *Trachypithecus barbei* | 0 | 70 | 10 | 10 | 0 | 0 | 0 | 0 | 10 | 0 | 0 | 0 | 0 | 0 | 0 | 0 | 0 | 0 | 0 | Fl |
| *Trachypithecus cristatus* | 0 | 70 | 10 | 10 | 0 | 0 | 0 | 0 | 10 | 0 | 0 | 0 | 0 | 0 | 0 | 0 | 0 | 0 | 0 | Fl |
| *Trachypithecus delacouri* | 0 | 78 | 8 | 7 | 0 | 0 | 0 | 0 | 7 | 0 | 0 | 0 | 0 | 0 | 0 | 0 | 0 | 0 | 0 | Fl |
| *Trachypithecus francoisi* | 0 | 50 | 17 | 7 | 0 | 0 | 0 | 0 | 14 | 0 | 7 | 0 | 5 | 0 | 0 | 0 | 0 | 0 | 0 | Fl |
| *Trachypithecus geei* | 0 | 24 | 24 | 24 | 0 | 0 | 0 | 0 | 24 | 0 | 0 | 0 | 4 | 0 | 0 | 0 | 0 | 0 | 0 | SF |
| *Trachypithecus germaini* | 0 | 25 | 25 | 25 | 0 | 0 | 0 | 0 | 25 | 0 | 0 | 0 | 0 | 0 | 0 | 0 | 0 | 0 | 0 | SF |
| *Trachypithecus hatinhensis* | 0 | 66 | 10 | 10 | 0 | 0 | 0 | 0 | 10 | 0 | 0 | 0 | 4 | 0 | 0 | 0 | 0 | 0 | 0 | Fl |
| *Trachypithecus johnii* | 0 | 70 | 10 | 10 | 0 | 0 | 0 | 0 | 10 | 0 | 0 | 0 | 0 | 0 | 0 | 0 | 0 | 0 | 0 | Fl |
| *Trachypithecus laotum* | 0 | 66 | 10 | 10 | 0 | 0 | 0 | 0 | 10 | 0 | 0 | 0 | 4 | 0 | 0 | 0 | 0 | 0 | 0 | Fl |
| *Trachypithecus obscurus* | 0 | 66 | 10 | 10 | 0 | 0 | 0 | 0 | 10 | 0 | 0 | 0 | 4 | 0 | 0 | 0 | 0 | 0 | 0 | Fl |
| *Trachypithecus phayrei* | 0 | 74 | 13 | 13 | 0 | 0 | 0 | 0 | 0 | 0 | 0 | 0 | 0 | 0 | 0 | 0 | 0 | 0 | 0 | Fl |
| *Trachypithecus pileatus* | 0 | 60 | 20 | 20 | 0 | 0 | 0 | 0 | 0 | 0 | 0 | 0 | 0 | 0 | 0 | 0 | 0 | 0 | 0 | Fl |
| *Trachypithecus poliocephalus* | 0 | 66 | 10 | 10 | 0 | 0 | 0 | 0 | 10 | 0 | 0 | 0 | 4 | 0 | 0 | 0 | 0 | 0 | 0 | Fl |
| *Trachypithecus shortridgei* | 0 | 70 | 10 | 10 | 0 | 0 | 0 | 0 | 10 | 0 | 0 | 0 | 0 | 0 | 0 | 0 | 0 | 0 | 0 | Fl |
| *Trachypithecus vetulus* | 0 | 70 | 10 | 10 | 0 | 0 | 0 | 0 | 10 | 0 | 0 | 0 | 0 | 0 | 0 | 0 | 0 | 0 | 0 | Fl |
| *Gorilla beringei* | 0 | 65 | 5 | 5 | 0 | 0 | 0 | 5 | 0 | 0 | 10 | 5 | 5 | 0 | 0 | 0 | 0 | 0 | 0 | Fl |

| | | | | | | | | | | | | | | | | | | | | |
|---|---|---|---|---|---|---|---|---|---|---|---|---|---|---|---|---|---|---|---|---|
| *Gorilla gorilla* | 0 | 15 | 80 | 0 | 0 | 0 | 0 | 0 | 0 | 0 | 0 | 0 | 5 | 0 | 0 | 0 | 0 | 0 | 0 | Fr |
| *Pan paniscus* | 0 | 10 | 50 | 9 | 0 | 0 | 0 | 19 | 9 | 0 | 0 | 3 | 0 | 0 | 0 | 0 | 0 | 0 | 0 | Fr |
| *Pan troglodytes* | 0 | 18 | 30 | 5 | 0 | 0 | 0 | 0 | 25 | 0 | 2 | 0 | 7 | 0 | 0 | 5 | 5 | 3 | 0 | Om |
| *Pongo abelii* | 0 | 24 | 59 | 6 | 0 | 0 | 0 | 0 | 0 | 0 | 4 | 0 | 5 | 0 | 0 | 1 | 1 | 0 | 0 | Fr |
| *Pongo pygmaeus* | 0 | 10 | 65 | 6 | 0 | 0 | 0 | 0 | 0 | 0 | 6 | 0 | 6 | 0 | 0 | 6 | 1 | 0 | 0 | Fr |
| *Bunopithecus hoolock* | 0 | 10 | 75 | 5 | 0 | 0 | 0 | 0 | 0 | 0 | 0 | 0 | 10 | 0 | 0 | 0 | 0 | 0 | 0 | Fr |
| *Hylobates agilis* | 0 | 10 | 74 | 10 | 0 | 0 | 0 | 0 | 0 | 0 | 0 | 0 | 3 | 0 | 0 | 3 | 0 | 0 | 0 | Fr |
| *Hylobates albibarbis* | 0 | 10 | 74 | 10 | 0 | 0 | 0 | 0 | 0 | 0 | 0 | 0 | 3 | 0 | 0 | 3 | 0 | 0 | 0 | Fr |
| *Hylobates klossii* | 0 | 10 | 74 | 10 | 0 | 0 | 0 | 0 | 0 | 0 | 0 | 0 | 3 | 0 | 0 | 3 | 0 | 0 | 0 | Fr |
| *Hylobates lar* | 0 | 30 | 50 | 9 | 0 | 0 | 0 | 0 | 0 | 0 | 0 | 0 | 11 | 0 | 0 | 0 | 0 | 0 | 0 | Fr |
| *Hylobates moloch* | 0 | 10 | 74 | 10 | 0 | 0 | 0 | 0 | 0 | 0 | 0 | 0 | 3 | 0 | 0 | 3 | 0 | 0 | 0 | Fr |
| *Hylobates muelleri* | 0 | 10 | 80 | 10 | 0 | 0 | 0 | 0 | 0 | 0 | 0 | 0 | 0 | 0 | 0 | 0 | 0 | 0 | 0 | Fr |
| *Hylobates pileatus* | 0 | 14 | 69 | 0 | 0 | 0 | 0 | 0 | 0 | 0 | 0 | 0 | 14 | 0 | 0 | 3 | 0 | 0 | 0 | Fr |
| *Nomascus concolor* | 0 | 20 | 72 | 5 | 0 | 0 | 0 | 0 | 0 | 0 | 0 | 0 | 0 | 0 | 0 | 3 | 0 | 0 | 0 | Fr |
| *Nomascus gabriellae* | 0 | 11 | 75 | 11 | 0 | 0 | 0 | 0 | 0 | 0 | 0 | 0 | 3 | 0 | 0 | 0 | 0 | 0 | 0 | Fr |
| *Nomascus hainanus* | 0 | 5 | 90 | 5 | 0 | 0 | 0 | 0 | 0 | 0 | 0 | 0 | 0 | 0 | 0 | 0 | 0 | 0 | 0 | Fr |
| *Nomascus leucogenys* | 0 | 11 | 75 | 11 | 0 | 0 | 0 | 0 | 0 | 0 | 0 | 0 | 3 | 0 | 0 | 0 | 0 | 0 | 0 | Fr |
| *Nomascus siki* | 0 | 5 | 90 | 5 | 0 | 0 | 0 | 0 | 0 | 0 | 0 | 0 | 0 | 0 | 0 | 0 | 0 | 0 | 0 | Fr |
| *Symphalangus syndactylus* | 0 | 20 | 72 | 5 | 0 | 0 | 0 | 0 | 0 | 0 | 0 | 0 | 0 | 0 | 0 | 3 | 0 | 0 | 0 | Fr |
| *Avahi unicolor* | 0 | 100 | 0 | 0 | 0 | 0 | 0 | 0 | 0 | 0 | 0 | 0 | 0 | 0 | 0 | 0 | 0 | 0 | 0 | Fl |
| *Indri indri* | 0 | 74 | 13 | 13 | 0 | 0 | 0 | 0 | 0 | 0 | 0 | 0 | 0 | 0 | 0 | 0 | 0 | 0 | 0 | Fl |
| *Propithecus coquereli* | 0 | 70 | 10 | 10 | 0 | 0 | 0 | 0 | 5 | 0 | 5 | 0 | 0 | 0 | 0 | 0 | 0 | 0 | 0 | Fl |
| *Propithecus deckenii* | 0 | 74 | 13 | 13 | 0 | 0 | 0 | 0 | 0 | 0 | 0 | 0 | 0 | 0 | 0 | 0 | 0 | 0 | 0 | Fl |
| *Propithecus diadema* | 0 | 74 | 13 | 13 | 0 | 0 | 0 | 0 | 0 | 0 | 0 | 0 | 0 | 0 | 0 | 0 | 0 | 0 | 0 | Fl |
| *Propithecus edwardsi* | 0 | 40 | 8 | 8 | 0 | 0 | 0 | 0 | 40 | 0 | 0 | 4 | 0 | 0 | 0 | 0 | 0 | 0 | 0 | SF |
| *Propithecus perrieri* | 0 | 50 | 17 | 16 | 0 | 0 | 0 | 0 | 17 | 0 | 0 | 0 | 0 | 0 | 0 | 0 | 0 | 0 | 0 | Fl |
| *Propithecus tattersalli* | 0 | 35 | 37 | 9 | 0 | 0 | 0 | 0 | 17 | 0 | 2 | 0 | 0 | 0 | 0 | 0 | 0 | 0 | 0 | SF |
| *Propithecus verreauxi* | 0 | 70 | 9 | 8 | 0 | 0 | 0 | 0 | 5 | 0 | 8 | 0 | 0 | 0 | 0 | 0 | 0 | 0 | 0 | Fl |
| *Eulemur albifrons* | 0 | 12 | 64 | 12 | 0 | 0 | 0 | 0 | 0 | 0 | 0 | 0 | 12 | 0 | 0 | 0 | 0 | 0 | 0 | Fr |
| *Eulemur collaris* | 0 | 12 | 60 | 12 | 0 | 0 | 0 | 0 | 5 | 0 | 0 | 0 | 11 | 0 | 0 | 0 | 0 | 0 | 0 | Fr |
| *Eulemur coronatus* | 0 | 20 | 70 | 5 | 0 | 0 | 0 | 0 | 0 | 0 | 0 | 0 | 5 | 0 | 0 | 0 | 0 | 0 | 0 | Fr |

| | | | | | | | | | | | | | | | | | | | | |
|---|---|---|---|---|---|---|---|---|---|---|---|---|---|---|---|---|---|---|---|---|
| *Eulemur fulvus* | 0 | 58 | 8 | 7 | 0 | 0 | 0 | 0 | 0 | 0 | 7 | 10 | 10 | 0 | 0 | 0 | 0 | 0 | 0 | Fl |
| *Eulemur macaco* | 0 | 5 | 48 | 37 | 0 | 0 | 0 | 0 | 0 | 0 | 5 | 5 | 0 | 0 | 0 | 0 | 0 | 0 | 0 | Fr |
| *Eulemur rubriventer* | 0 | 12 | 61 | 12 | 0 | 0 | 0 | 0 | 0 | 0 | 0 | 0 | 15 | 0 | 0 | 0 | 0 | 0 | 0 | Fr |
| *Eulemur rufus* | 0 | 12 | 61 | 12 | 0 | 0 | 0 | 0 | 0 | 0 | 0 | 0 | 15 | 0 | 0 | 0 | 0 | 0 | 0 | Fr |
| *Eulemur sanfordi* | 0 | 15 | 65 | 15 | 0 | 0 | 0 | 0 | 0 | 0 | 0 | 0 | 5 | 0 | 0 | 0 | 0 | 0 | 0 | Fr |
| *Varecia rubra* | 0 | 5 | 95 | 0 | 0 | 0 | 0 | 0 | 0 | 0 | 0 | 0 | 0 | 0 | 0 | 0 | 0 | 0 | 0 | Fr |
| *Varecia variegata* | 0 | 10 | 80 | 10 | 0 | 0 | 0 | 0 | 0 | 0 | 0 | 0 | 0 | 0 | 0 | 0 | 0 | 0 | 0 | Fr |
| *Elephas maximus* | 60 | 15 | 0 | 0 | 0 | 5 | 0 | 5 | 0 | 5 | 10 | 0 | 0 | 0 | 0 | 0 | 0 | 0 | 0 | Gr |
| *Loxodonta africana* | 16 | 17 | 16 | 0 | 0 | 0 | 0 | 17 | 0 | 2 | 32 | 0 | 0 | 0 | 0 | 0 | 0 | 0 | 0 | GH |
| *Loxodonta cyclotis* | 0 | 34 | 34 | 0 | 0 | 0 | 0 | 0 | 0 | 0 | 32 | 0 | 0 | 0 | 0 | 0 | 0 | 0 | 0 | SF |
| *Castor canadensis* | 0 | 15 | 0 | 0 | 0 | 0 | 0 | 15 | 0 | 5 | 65 | 0 | 0 | 0 | 0 | 0 | 0 | 0 | 0 | GH |
| *Castor fiber* | 0 | 20 | 5 | 5 | 0 | 0 | 0 | 10 | 0 | 20 | 40 | 0 | 0 | 0 | 0 | 0 | 0 | 0 | 0 | GH |
| *Dolichotis patagonum* | 70 | 15 | 4 | 3 | 0 | 0 | 0 | 0 | 8 | 0 | 0 | 0 | 0 | 0 | 0 | 0 | 0 | 0 | 0 | Gr |
| *Dolichotis salinicola* | 8 | 38 | 8 | 0 | 0 | 30 | 0 | 0 | 8 | 0 | 8 | 0 | 0 | 0 | 0 | 0 | 0 | 0 | 0 | GH |
| *Hydrochoerus hydrochaeris* | 45 | 0 | 10 | 0 | 0 | 0 | 0 | 0 | 0 | 35 | 10 | 0 | 0 | 0 | 0 | 0 | 0 | 0 | 0 | MF |
| *Hydrochoerus isthmius* | 45 | 0 | 10 | 0 | 0 | 0 | 0 | 0 | 0 | 35 | 10 | 0 | 0 | 0 | 0 | 0 | 0 | 0 | 0 | MF |
| *Cuniculus paca* | 0 | 10 | 60 | 0 | 0 | 0 | 0 | 20 | 10 | 0 | 0 | 0 | 0 | 0 | 0 | 0 | 0 | 0 | 0 | Fr |
| *Cuniculus taczanowskii* | 0 | 0 | 50 | 0 | 0 | 0 | 0 | 0 | 50 | 0 | 0 | 0 | 0 | 0 | 0 | 0 | 0 | 0 | 0 | Fr |
| *Dinomys branickii* | 0 | 45 | 45 | 0 | 0 | 0 | 0 | 0 | 10 | 0 | 0 | 0 | 0 | 0 | 0 | 0 | 0 | 0 | 0 | SF |
| *Hystrix africaeaustralis* | 0 | 0 | 15 | 0 | 0 | 0 | 0 | 60 | 0 | 0 | 15 | 0 | 5 | 0 | 5 | 0 | 0 | 0 | 0 | Om |
| *Hystrix brachyura* | 0 | 0 | 15 | 0 | 0 | 0 | 0 | 60 | 0 | 0 | 15 | 0 | 5 | 0 | 5 | 0 | 0 | 0 | 0 | Om |
| *Hystrix crassispinis* | 0 | 0 | 33 | 0 | 0 | 0 | 0 | 67 | 0 | 0 | 0 | 0 | 0 | 0 | 0 | 0 | 0 | 0 | 0 | Om |
| *Hystrix cristata* | 0 | 0 | 15 | 0 | 0 | 10 | 0 | 50 | 0 | 0 | 10 | 0 | 5 | 0 | 5 | 5 | 0 | 0 | 0 | Om |
| *Hystrix indica* | 0 | 0 | 20 | 0 | 0 | 0 | 0 | 40 | 20 | 0 | 20 | 0 | 0 | 0 | 0 | 0 | 0 | 0 | 0 | Om |
| *Hystrix javanica* | 20 | 20 | 20 | 0 | 0 | 20 | 0 | 20 | 0 | 0 | 0 | 0 | 0 | 0 | 0 | 0 | 0 | 0 | 0 | GH |
| *Hystrix pumila* | 0 | 0 | 28 | 0 | 0 | 0 | 0 | 57 | 0 | 0 | 0 | 0 | 5 | 0 | 5 | 5 | 0 | 0 | 0 | Om |
| *Hystrix sumatrae* | 0 | 0 | 24 | 0 | 0 | 0 | 0 | 47 | 0 | 0 | 24 | 0 | 0 | 0 | 5 | 0 | 0 | 0 | 0 | Om |
| *Myocastor coypus* | 0 | 10 | 0 | 0 | 0 | 0 | 0 | 10 | 0 | 70 | 10 | 0 | 0 | 0 | 0 | 0 | 0 | 0 | 0 | GH |
| *Marmota baibacina* | 48 | 0 | 0 | 0 | 0 | 48 | 0 | 0 | 0 | 0 | 0 | 0 | 4 | 0 | 0 | 0 | 0 | 0 | 0 | MF |
| *Marmota bobak* | 50 | 0 | 0 | 0 | 0 | 30 | 0 | 10 | 10 | 0 | 0 | 0 | 0 | 0 | 0 | 0 | 0 | 0 | 0 | MF |
| *Marmota broweri* | 20 | 0 | 20 | 0 | 0 | 38 | 0 | 0 | 18 | 0 | 0 | 0 | 4 | 0 | 0 | 0 | 0 | 0 | 0 | GH |

| | | | | | | | | | | | | | | | | | | | | |
|---|---|---|---|---|---|---|---|---|---|---|---|---|---|---|---|---|---|---|---|---|
| *Marmota caligata* | 25 | 25 | 0 | 25 | 0 | 0 | 0 | 0 | 0 | 25 | 0 | 0 | 0 | 0 | 0 | 0 | 0 | 0 | 0 | MF |
| *Marmota camtschatica* | 14 | 10 | 14 | 0 | 0 | 10 | 0 | 20 | 20 | 0 | 8 | 0 | 4 | 0 | 0 | 0 | 0 | 0 | 0 | GH |
| *Marmota caudata* | 33 | 33 | 0 | 0 | 0 | 34 | 0 | 0 | 0 | 0 | 0 | 0 | 0 | 0 | 0 | 0 | 0 | 0 | 0 | MF |
| *Marmota himalayana* | 14 | 14 | 14 | 0 | 0 | 14 | 0 | 20 | 24 | 0 | 0 | 0 | 0 | 0 | 0 | 0 | 0 | 0 | 0 | GH |
| *Marmota marmota* | 14 | 14 | 0 | 14 | 0 | 15 | 0 | 14 | 14 | 0 | 0 | 0 | 15 | 0 | 0 | 0 | 0 | 0 | 0 | GH |
| *Marmota menzbieri* | 14 | 14 | 14 | 0 | 0 | 14 | 0 | 20 | 24 | 0 | 0 | 0 | 0 | 0 | 0 | 0 | 0 | 0 | 0 | GH |
| *Marmota monax* | 42 | 7 | 10 | 0 | 0 | 0 | 0 | 0 | 14 | 0 | 10 | 0 | 10 | 0 | 0 | 7 | 0 | 0 | 0 | MF |
| *Marmota sibirica* | 80 | 0 | 0 | 0 | 0 | 20 | 0 | 0 | 0 | 0 | 0 | 0 | 0 | 0 | 0 | 0 | 0 | 0 | 0 | Gr |
| *Marmota vancouverensis* | 30 | 5 | 15 | 20 | 0 | 30 | 0 | 0 | 0 | 0 | 0 | 0 | 0 | 0 | 0 | 0 | 0 | 0 | 0 | GH |
| *Orycteropus afer* | 0 | 0 | 0 | 0 | 0 | 0 | 0 | 0 | 0 | 0 | 0 | 0 | 100 | 0 | 0 | 0 | 0 | 0 | 0 | IF |
